# Leveraging Administrative Data to Better Understand and Address Child Maltreatment: A Scoping Review of Data Linkage Studies

**DOI:** 10.1177/10775595221079308

**Published:** 2022-03-03

**Authors:** Emma Soneson, Shruti Das, Anne-Marie Burn, Marije van Melle, Joanna K. Anderson, Mina Fazel, Peter Fonagy, Tamsin Ford, Ruth Gilbert, Katie Harron, Emma Howarth, Ayla Humphrey, Peter B. Jones, Anna Moore

**Affiliations:** 1 Department of Psychiatry, 2152 University of Cambridge, Cambridge, UK; 2 12204 University of Cambridge School of Clinical Medicine, Cambridge, UK; 3 Department of Psychiatry, Warneford Hospital, 6396 University of Oxford, Headington, Oxford, UK; 4 Research Department of Clinical, Educational and Health Psychology, 4919 University College London, London, UK; 5 Great Ormond Street Institute of Child Health, 4919 University College London, London, UK; 6 School of Psychology, 4917 University of East London, London, UK

**Keywords:** child maltreatment, abuse, neglect, data linkage, administrative data, data analytics, policy, public health, population health

## Abstract

**Background:**

This scoping review aimed to overview studies that used administrative data linkage in the context of child maltreatment to improve our understanding of the value that data linkage may confer for policy, practice, and research.

**Methods:**

We searched MEDLINE, Embase, PsycINFO, CINAHL, and ERIC electronic databases in June 2019 and May 2020 for studies that linked two or more datasets (at least one of which was administrative in nature) to study child maltreatment. We report findings with numerical and narrative summary.

**Results:**

We included 121 studies, mainly from the United States or Australia and published in the past decade. Data came primarily from social services and health sectors, and linkage processes and data quality were often not described in sufficient detail to align with current reporting guidelines. Most studies were descriptive in nature and research questions addressed fell under eight themes: descriptive epidemiology, risk factors, outcomes, intergenerational transmission, predictive modelling, intervention/service evaluation, multi-sector involvement, and methodological considerations/advancements.

**Conclusions:**

Included studies demonstrated the wide variety of ways in which data linkage can contribute to the public health response to child maltreatment. However, how research using linked data can be translated into effective service development and monitoring, or targeting of interventions, is underexplored in terms of privacy protection, ethics and governance, data quality, and evidence of effectiveness.

## Introduction

Child maltreatment is an important public health problem that has received significant attention in terms of national and international policy and intervention efforts (Domestic Violence Act, 2021; HM Government, 2018; World Health Organization, 2019, 2020). Whilst the exact prevalence of child maltreatment is difficult to measure, estimates from self-report surveys conducted in high-income countries indicate that a sizeable minority of children experience maltreatment each year (4–16% experience physical abuse, 10% experience psychological abuse, 1–15% experience neglect, and 10–20% are exposed to intimate-partner violence), and many experience more than one type of maltreatment (Gilbert, Widom, et al., 2009). The effects of maltreatment are far-reaching, ranging from increased risk for abnormal development and poor mental health outcomes (Gilbert et al., 2009; Maguire et al., 2015; Norman et al., 2012; Scott et al., 2010) to learning problems and peer rejection (Gilbert, Widom, et al., 2009; Maguire et al., 2015), all of which can contribute to significant social and economic consequences across the lifespan.

Uncertainties around the prevalence, aetiology, and trajectories of child maltreatment complicate the design and implementation of an effective public health response (Herrenkohl et al., 2015). For example, the true prevalence and incidence of child maltreatment are largely uncertain, as it is often under-reported to services and under-recorded in official agency data (Fallon et al., 2010; Gilbert, Kemp, et al., 2009; Gilbert, Widom, et al., 2009; N. V. Lewis et al., 2018; McTavish et al., 2017). Furthermore, whilst a consensus now exists that child maltreatment cannot be understood without adopting a whole system perspective (DePasquale et al., 2019; Wathen et al., 2012), the complex, multi-level structure of (Belsky, 1993) and interaction between (Baldwin et al., 2020) risk and protective factors makes it difficult to accurately predict and therefore identify which children may be at risk of experiencing maltreatment.

Administrative data (i.e. information collected as part of day-to-day operations, for example within child protection agencies) have long been recognised as a valuable resource for addressing questions relating to child maltreatment (Drake & Jonson-Reid, 1999; Hurren et al., 2017b). Administrative data are often collected at a population level (Hurren et al., 2017b), which confers many benefits. Analysing data collected ‘at scale’ can reduce biases (e.g. selection or recall biases) (Hurren et al., 2017b; Penner & Dodge, 2019), contextualise individuals within their wider environments (Penner & Dodge, 2019), facilitate the study of small sub-groups (Hurren et al., 2017b), and highlight and describe inequalities (Penner & Dodge, 2019). These data facilitate ‘quasi-prospective’ analyses, allowing researchers to follow time trends on individual and population levels using data collected in ‘real-time’ (Brownell & Jutte, 2013; Roos et al., 2008). On a practical level, using administrative data for research purposes is often time- and cost-efficient for researchers and policymakers if the data exist within a strong infrastructure (Penner & Dodge, 2019). Furthermore, by using these data, researchers can reduce the burden on individual participants, gather information on individuals who are not likely to take part in primary research, and encourage honest and accurate responses about difficult topics such as maltreatment (Connelly et al., 2016).

Administrative data also have several benefits that may make them particularly useful in terms of designing, implementing, and evaluating policies and interventions (Connelly et al., 2016; Hurren et al., 2017b; Penner & Dodge, 2019), which is essential for providing an effective public health response. These data offer a long-term perspective that might otherwise be difficult to examine using other methodologies (e.g. self-report surveys) (Connelly et al., 2016; Penner & Dodge, 2019), and the society-level perspective facilitates the study of feedback loops and ‘spill-over’ effects that may occur when policies and interventions are implemented at scale (Penner & Dodge, 2019). Furthermore, administrative data may be a particularly relevant and useful information source for policymakers, who are often judged by these very metrics and outcomes, and as such may be more inclined to act on the results of research making use of these data (Penner & Dodge, 2019).

However, administrative data are not without disadvantages. As described above, it is widely acknowledged that administrative data under-report the true prevalence of maltreatment, particularly for less overt types of maltreatment (Fallon et al., 2010; Gilbert, Kemp, et al., 2009; Gilbert, Widom, et al., 2009; N. V. Lewis et al., 2018; McTavish et al., 2017), and there is wide variation in data quality and completeness (Hurren et al., 2017b; N. V. Lewis et al., 2018; McTavish et al., 2017; Putnam-Hornstein, Needell, & Rhodes, 2013; Syed et al., 2021). Issues with data quality may lead to negative consequences including underestimation of need (Schnitzer et al., 2011), biased results, and exacerbated inequalities (Knight et al., 2021). Another limitation of administrative data is that researchers have no control over which variables are collected (Hurren et al., 2017b; Roos et al., 2008), which may limit the breadth or depth of possible analyses. Furthermore, in the absence of strong infrastructure, these data can be difficult or time-consuming to access (Cavallaro et al., 2020; Connelly et al., 2016; Hurren et al., 2017b; Penner & Dodge, 2019; Taylor et al., 2021). Finally, many have raised concerns about ethical and legal issues related to the use of administrative data (Brownell & Jutte, 2013; Connelly et al., 2016; Jonson-Reid & Drake, 2008; Penner & Dodge, 2019). However, in many cases these can be minimised by use of de-identified data and strict controls to prevent re-identification. These processes enable use of whole-population data without individual consent, provided privacy is protected and individuals are not re-identifiable.

The value of administrative data can be enhanced through data linkage (Jonson-Reid & Drake, 2008), an approach in which information from multiple sources is combined to create more comprehensive databases (Gilbert et al., 2018; Russ et al., 2019; Spiranovic et al., 2016). In addition to sharing the general benefits of administrative data described above, triangulation of administrative data from a range of sources, as well as linkage to data collected for research purposes, has unique advantages in terms of addressing some of the difficulties related to studying maltreatment (Brownell & Jutte, 2013; Penner & Dodge, 2019; Prinz, 2017). Critically, linked administrative data from a wide range of settings offer the opportunity to study risk and protective factors and outcomes across multiple and overlapping domains (Jutte et al., 2011), an approach that is well-aligned with our understanding that maltreatment exerts a broad impact across multiple domains of biological, psychological, and social development (Belsky, 1993; Toth & Cicchetti, 2013). Linkage between children and their parents/siblings can also help contextualise the child within the family (Howard et al., 2019; Jutte et al., 2011; Roos et al., 2008) and enable the study of risk factors and outcomes throughout multiple generations, which is difficult to achieve using more traditional approaches (Brownell & Jutte, 2013; Putnam-Hornstein et al., 2015).

Data linkage also has potential to aid in the design, implementation, and evaluation of interventions, services, and policies to prevent or respond to child maltreatment (Brownell & Jutte, 2013; Howard et al., 2019; Jonson-Reid & Drake, 2008). These functions can be achieved using identifiable or de-identified data. For example, linking de-identified data from multiple agencies can be a useful tool for mapping service use (Howard et al., 2019; Jutte et al., 2011; Penner & Dodge, 2019; Putnam-Hornstein & Needell, 2011) and can provide insight into important questions including who is (or is not) accessing which services, whereas using identifiable linked data (with consent) can help researchers track individual outcomes over time and across a broad range of domains.

Whilst linked data share many of the limitations related to the source data (discussed above), there are also unique limitations. For example, issues of interoperability are common, given variations in data structure, content, and format (Harron et al., 2017). Furthermore, there is often uncertainty as to the legality of sharing and linking data across organisations (Mourby et al., 2019), which may limit the potential contribution of linked data to research and policy (Harron et al., 2017; Penner & Dodge, 2019). Implementing and maintaining successful data linkage systems requires strong supporting infrastructure and information governance systems, the development of which requires significant cost and time investment (Mourby et al., 2019).

Another important consideration relates to data quality. The quality of linked administrative data can be understood in terms of the quality of the source data, the accuracy of the linkage, and the presence of biases particular to this methodology (Gilbert et al., 2018). Linkage quality is a key consideration, as linkage errors, comprised of false matches and missed matches, can lead to information bias and selection bias (Doidge & Harron, 2019; Harron et al., 2014). Furthermore, errors are more likely to occur for minority and vulnerable groups, which can potentially lead to an underestimation of need for these individuals (Bohensky et al., 2010; Doidge & Harron, 2019; McGrath-Lone et al., 2021). Error rates also vary by the linkage technique and specific decisions made by researchers during this process. Depending on the availability and quality of unique identifiers across records, researchers may opt to use a deterministic or probabilistic linkage technique (or a combination of the two). Deterministic linkage, wherein a set of pre-determined rules is used to decide whether records belong to the same individual, can be more vulnerable to missed matches, but typically results in low rates of false matches. Probabilistic linkage, which can be an effective and accurate technique when there is an absence of (reliable) unique identifiers (Campbell et al., 2008; Gill et al., 1993; Putnam-Hornstein et al., 2011), links records using match weights derived from probabilities related to (dis)agreement on a set of identifiers. During this process, researchers set a threshold in order to balance missed and false matches, though choosing an ‘optimal’ threshold is often not straightforward (Harron et al., 2017). Whilst there is established guidance for how to report on studies using (linked) administrative data, including the RECORD Statement (Benchimol et al., 2015) and GUILD guidance (Gilbert et al., 2018), a recent review of studies using administrative data linked with longitudinal data from child protection settings found that only three of the thirty included studies reported data linkage processes in enough detail to adequately conform with the recommendations of these guidelines (Chikwava et al., 2021).

These limitations notwithstanding, data linkage has potential to aid in the public health response to child maltreatment. Whilst there are many theoretical uses for data linkage in this field (e.g. understanding the aetiology and consequences of maltreatment, informing intervention and policy design, facilitating recruitment into trials, and enabling systematic evaluation of interventions and policy initiatives in real-life settings), a broad overview of how this approach is currently being used could promote a better understanding of its real-world uses, advantages, and limitations. Therefore, the aim of this scoping review was to identify and describe studies that used data linkage in the context of child maltreatment in order to improve our understanding of the value that data linkage may confer for policy, practice, and research. Our objectives were to (1) describe which data (from which sectors) have been linked, (2) overview linkage processes, (3) identify the main purposes/uses of data linkage in the context of child maltreatment, and (4) overview the types of questions being addressed using data linkage.

## Methods

We conducted a scoping review due to its usefulness for both for ‘mapping’ the evidence by examining the extent, range, and nature of research in the area as well as for identifying gaps in the literature (Anderson et al., 2020; Arksey & O’Malley, 2005). We followed Anderson and colleagues’ (2020) recommendations for using systematic procedures for our literature searches, study selection, data extraction, and data analysis. Given the broad nature of our objectives, we did not conduct quality assessment of included articles, which is in line with Arksey and O’Malley’s (2005) recommendations for scoping reviews. Whilst PROSPERO does not currently accept pre-registrations of scoping reviews, we followed a written protocol and report findings in accordance with the Preferred Reporting Items for Systematic reviews and Meta-Analyses extension for Scoping Reviews (PRISMA-ScR) (Tricco et al., 2018).

### Identifying relevant studies

We searched MEDLINE, Embase, PsycINFO, CINAHL, and ERIC electronic databases in June 2019, updated in May 2020, for potentially relevant studies and gathered additional references through backward citation searching of key studies and supplemental searches in PubMed and Google Scholar. Our search strategy (Supplementary Table 1), developed in partnership with a medical librarian and data linkage expert, combined terms for data linkage with terms for children and young people (we did not include specific terms for child maltreatment because this review was part of a larger review with a wider scope).

When we found fewer studies than expected from the Nordic countries, we consulted a Swedish researcher in the field about our search terms and subsequently completed supplemental PubMed and Google Scholar searches including terms for ‘register/registry' data (these yielded only six additional papers that met inclusion criteria).

### Inclusion/exclusion criteria

Our population of interest was individuals who experienced child maltreatment before age 18, as ascertained by indicators in administrative records. We followed Gilbert and colleagues’ definition of maltreatment: ‘Any act of commission or omission by a parent or other caregiver that results in harm, potential for harm or threat of harm to a child. Harm does not need to be intended’ (Gilbert, Widom, et al., 2009, p. 69). We included studies that examined presentations of physical abuse, sexual abuse, emotional abuse, neglect, or exposure to intimate-partner violence and presentations could be a single event or a persistent/chronic condition. Maltreatment could be ascertained via child or parent administrative records, and we placed no restriction on the indicator used to ascertain maltreatment (e.g. allegations, investigations, substantiations, medical codes, or criminal justice records). However, studies focused on out-of-home (foster) care placement were eligible for inclusion only if authors specified that placement *was a direct result of maltreatment.* This is because out-of-home care placement is a *downstream intervention* rather than a *presentation of maltreatment,* and furthermore there are reasons other than maltreatment for which a child may enter out-of-home care.

We included studies linking at least two datasets for the same individual from different data-holding organisations but excluded studies of data linkage strictly within a single dataset (i.e. linkage of data for the same individual over time). Studies linking parental and child data were also eligible for inclusion. For a study to be included, at least one of the linked datasets had to be administrative and longitudinal (i.e. comprised of repeated observations over time). Furthermore, studies had to link at least two datasets comprised of *individual-level data*; studies focused *only* on linking one individual-level dataset to census-tract, area-level, or aggregate-level data were not eligible for inclusion. The linkage itself could occur at any time in the lifespan and with any length of follow-up, and we placed no restriction on processes or methods relating to linkage.

We also placed no restriction on study design (both peer-reviewed and grey literature were eligible for inclusion), setting, date, or language of publication (provided an abstract or summary was available in English).

### Study selection

Six reviewers (ES, SD, A-MB, MvM, JKA, and AM) independently double-screened all titles and abstracts against the inclusion/exclusion criteria and removed irrelevant records. If one or both reviewers judged that a record may meet inclusion criteria, it was included in the full-text screening round. Two reviewers (ES and SD) independently double-screened the full texts of all relevant records and resolved disagreements through discussion with a third reviewer (AM).

### Charting the data

The research team collaboratively developed a data-charting form, which two reviewers (ES and SD) piloted. The two reviewers independently extracted data from ten studies to ensure the data-charting form aligned with the research question and aims of the review (Levac et al., 2010). Extraction fields included first author, year of publication, country/ies of publication; study aims and stated outcomes of interest; study population/cohort characteristics; types of maltreatment studied; and summary of key study findings. In terms of the linkage, we extracted the following fields: purpose of linkage, description of source data, and description of the linkage. We determined the purposes of linkage inductively, and the final categories included descriptive, predictive modelling, service evaluation, and methodological advancement. Information about the source data included the names of the data sources, whether the datasets were administrative or study-specific (i.e. collected as part of a study/specific piece of research) in nature, whether studies were population-based, whether studies linked parent and child data, and which age range was covered in the dataset. Information about the linkage process included descriptions of consent requirements/processes, whether the data were used in identifiable or de-identified format, linkage technique (probabilistic/deterministic), frequency of linkage (retrospective one-time, repeated one-time, or near real time/‘living’), and quality assessment procedures (e.g. rates of linkage error, assessment of biases).

After the piloting phase, one reviewer (SD) completed all extractions and one of three others (ES, MvM, and AM) double-checked all fields for quality assurance. Where information was not included in a study (which was especially common for information on linkage processes), we consulted related papers and study websites, if available.

### Collating, summarising, and reporting the results

We collated and summarised results from included studies using descriptive numerical summary and narrative synthesis (Arksey & O’Malley, 2005; Levac et al., 2010). The numerical summary provided an overall description of the characteristics of included studies, which was particularly useful for addressing Objectives 1, 2, and 3. To address Objective 4, four reviewers (ES, SD, A-MB, and AM) separately examined the completed data-charting form, focusing on each study’s aims, stated objective(s), and summary of findings. Based on these extractions, the reviewers independently grouped research questions into themes and then met to discuss these themes, identify agreement and disagreement across reviewers, and finalise which would be included in the review. We categorised the types of research questions addressed into the following types: (1) descriptive epidemiology, (2) risk factors, (3) outcomes, (4) intergenerational transmission, (5) predictive modelling, (6) intervention/service evaluation, (7) multi-sector involvement, and (8) methodological considerations and advancements (where there was overlap, we categorised based on the authors’ main aims). For each category, we also provide examples of what the authors of the included studies perceived as the benefits of using a data linkage approach. We report all data in a narrative format and, as recommended by Levac et al. (2010), highlight key implications for research, policy, and practice in our discussion.

## Results

### Overview of study characteristics

**Figure 1.**
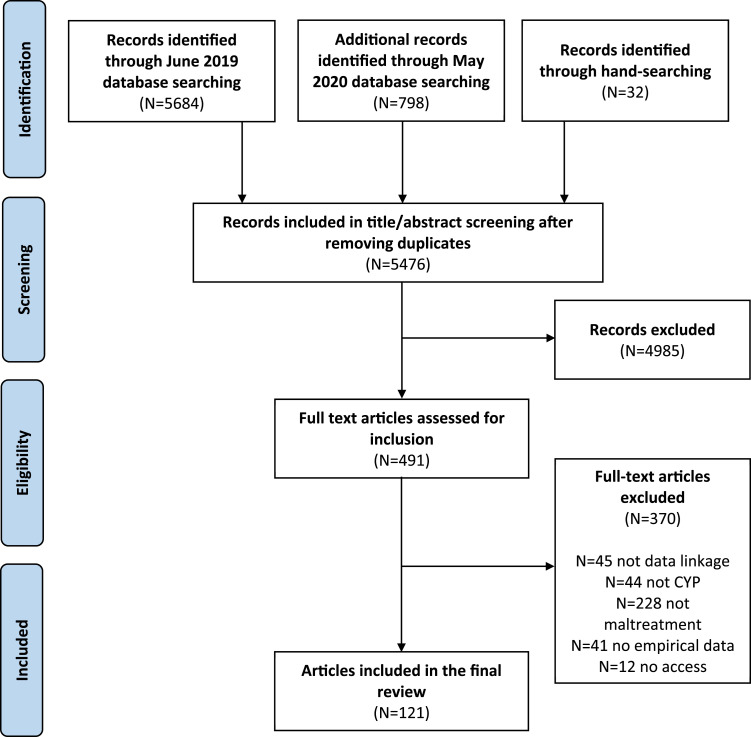
PRISMA flow diagram.

A total of 121 studies met criteria for inclusion in the review. Figure 1 provides a PRISMA flow diagram for the review and Table 1 provides an account of included studies. Supplementary Table 2 provides the completed data-charting form with key study characteristics and findings, organised by theme.

**Table 1.** Account of included studies.

Characteristic	Number of Studies
Year	
*Pre-2000*	3
*2000 to 2005*	8
*2006 to 2010*	11
*2011 to 2015*	33
*2016 to 2020*	66
Country^a^	
*Australia*	38
*Canada*	3
*Denmark*	2
*Finland*	1
*New Zealand*	6
*Norway*	3
*Sweden*	5
*USA*	64
*Purpose of linkage*^[Table-fn table-fn2-10775595221079308]^	
*Descriptive*	110
*Intervention or service evaluation*	9
*Methodological considerations/advancement*	6
*Predictive modelling*	4
Population-based	
*Yes*	103
*No*	18
Included study-specific (research) data	
*No*	102
*Yes*	19
Data sources	
*Health & health*	6
*Health & justice*	1
*Health & justice & other (aggregate) data*	2
*Health & study-specific*	1
*Health & other (aggregate) data*	1
*Social services & education*	4
*Social services & education & other (aggregate) data*	1
*Social services & health*	40
*Social services & health & education*	10
*Social services & health & education & other (aggregate) data*	2
*Social services & health & justice*	10
*Social services & health & justice & education*	8
*Social services & health & justice & education & other (aggregate) data*	1
*Social services & health & justice & other (aggregate) data*	1
*Social services & health & study specific*	2
*Social services & health & other (aggregate) data*	7
*Social services & justice*	4
*Social services & justice & other (aggregate) data*	1
*Social services & social services*	4
*Social services & study-specific*	12
*Social services & other (aggregate) data*	3
Ethical basis for linkage	
*Consent obtained*	14
*Exempt/implied exempt from consent requirements*	37
*Not described*	70
Linkage technique	
*Deterministic*	16
*Probabilistic*	67
*Probabilistic and deterministic*	9
*Not described*	29
Type of linkage	
*Near real-time (‘living’ linkage) (prospective)*	15
*Repeated one-time or near real-time*	15
*Retrospective one-time*	81
*Insufficient information to categorise*	10
Linkage validation & quality assessment	
*Described*	70
*Not described*	51

^a^*N*>121 because some studies examined data linkage in multiple countries.

^b^*N*>121 because some studies had more than one purpose.

The results show an increasing attention over time to studying child maltreatment using data linkage, with most studies (*N*=99) published after 2010. Studies clustered within a small number of countries, with most taking place in the United States (*N*=64) or Australia (*N*=38). None of the studies were conducted in a low- or middle-income country.

There was wide variation in the included studies both in terms of types of maltreatment studied and operational definitions used. Most studies (*N*=83) took a broad view of child maltreatment, defining it as abuse (physical, sexual, and emotional) and neglect. Only three studies explicitly included witnessing intimate-partner violence within these broad definitions of maltreatment, and several (*N*=12) excluded emotional abuse and/or neglect from their definition. Eleven studies focused on only one or a subset of child maltreatment types (e.g. just sexual abuse). An additional eight studies focused on children placed in out-of-home care specifically due to maltreatment. Studies varied in the indicators they used to ascertain maltreatment, which included notifications, investigations, and substantiations (or a combination thereof) as well as maltreatment-related injuries and deaths.

### Objective 1. To describe which data (from which sectors) have been linked

Most studies (*N*=103) used population-based data and linked administrative data only (*N*=102), with the single most common linkage being between health and social services datasets (*N*=81). A smaller number of studies (*N*=19) included linkage to study-specific datasets (i.e. data collected for research purposes). Data from social services was included in 110 studies, health in 92 studies, justice in 28 studies and education in 26 studies. Child protective services records were the most common type of dataset, included in 96 studies. Among studies that stated them, samples sizes ranged from 345 to 4,317,321 (*N.B.* several studies using whole-of-population data did not explicitly report sample size).

### Objective 2. To overview linkage processes

In the context of established guidelines for reporting on studies using administrative data and data linkage (Benchimol et al., 2015; Gilbert et al., 2018), data linkage processes in the included studies were often lacking in detail. Most studies (*N*=81) used a retrospective, one-time linkage to create their dataset, with many fewer (*N*=30) using repeated one-time or near-real-time (‘living’) linkages. Around one-third of studies (*N*=40) reported using existing research databases, most commonly the Western Australia Data Linkage System (*N*=15) and the New South Wales Child Development Study (*N*=7). The ethical and legal bases for linkage were often not described, with only 51 studies stating whether consent was required/obtained (however, it is important to note that most studies used de-identified data, which will not have required consent, even if not explicitly stated in the study). Most studies (*N*=92) described the broad linkage technique (*N*=67 studies used probabilistic linkage, *N*=16 used deterministic linkage, and *N*=9 used both), but most did not provide any further technical detail (e.g. variables used to link datasets). Slightly more than half (*N*=70) of studies included any information on linkage validation or quality assessment (e.g. by providing rates of false links or unmatched records), but of these, most descriptions were inadequate for properly assessing data and linkage quality.

### Objective 3. To identify the main purposes/uses of data linkage in the context of child maltreatment

Nearly all studies in our review used data linkage for descriptive purposes (*N*=110), for example to generate prevalence/incidence estimates or examine risk factors or outcomes associated with child maltreatment. Very few studies used data linkage for other purposes, such as service/intervention evaluation (*N*=9) or predictive modelling (*N*=4). Six studies focused on methodological considerations of data linkage in this area, both in terms of linkage techniques themselves as well as their potential contributions to improving our understanding of child maltreatment.

### Objective 4. To overview the types of questions being addressed using data linkage

Below we summarise the types of questions addressed in the included studies, which we have grouped into eight themes; specific results pertaining to each included study can be found in the final column of Supplementary Table 2.

*1. Descriptive epidemiology:* Several studies investigated the prevalence or incidence of child maltreatment across whole populations within a specified region (Gessner et al., 2004; Gilbert et al., 2012; Högberg, Andersson, et al., 2018; Högberg, Lampa, et al., 2018; Parrish et al., 2020; Putnam-Hornstein et al., 2011; Rouland & Vaithianathan, 2018; Ryan et al., 2018; Schnitzer et al., 2008; Segal et al., 2019). Other studies reported on the prevalence of child maltreatment in specific groups, including adolescent mothers (Putnam-Hornstein, Cederbaum, King, Cleveland, et al., 2013), young adults accessing homelessness services (Putnam-Hornstein et al., 2017), children with disabilities (Maclean et al., 2017), and children with an Autism Spectrum Disorder (ASD) diagnosis (Fisher et al., 2019).

Perhaps the most widely acknowledged benefit of using data linkage to estimate the prevalence and incidence of child maltreatment was that the population-level data used allowed for the most complete estimates possible and included high-risk groups that might traditionally be under-represented when using other methodologies (e.g. Fisher et al., 2019; Högberg, Andersson, et al., 2018; Högberg, Lampa, et al., 2018; Maclean et al., 2017; Putnam-Hornstein et al., 2011, 2017). Whilst this represents a more general strength relating to the source data, there was also general agreement among studies that combining multiple data sources improved prevalence estimates by identifying additional cases over the use of a single dataset alone (e.g. Gilbert et al., 2012; Parrish et al., 2020; Schnitzer et al., 2008). However, authors noted that some datasets were more useful than others in ascertaining unique cases above and beyond what was available in, for example, standard CPS records.

*2. Risk factors for child maltreatment:* A large proportion of studies examined risk factors for child maltreatment. Several studies examined associations between maltreatment and maternal and perinatal risk factors, such as maternal marital status, maternal ethnicity, maternal age, maternal education level, engagement with prenatal care, smoking or substance use during pregnancy, alcohol or substance use after pregnancy, maternal experience of domestic violence or assault in the perinatal period, and maternal mental health disorders (Austin et al., 2018; Cant et al., 2019; Cram et al., 2015; Eastman & Putnam-Hornstein, 2019; Ekéus et al., 2004; Finno-Velasquez et al., 2017; Gessner et al., 2004; M. J. Green et al., 2018; Hafekost, Lawrence, O’Leary, Bower, O’Donnell, et al., 2017; Hafekost, Lawrence, O’Leary, Bower, Semmens, et al., 2017; Högberg et al., 2019; Johnson-Motoyama et al., 2015; Melissa O’Donnell et al., 2015; Orr et al., 2019; Parrish et al., 2011; 2016; Parrish & Gessner, 2010; Putnam-Hornstein, Needell, King, et al., 2013; Putnam-Hornstein & Needell, 2011).

On an individual level, child characteristics examined included birth weight/small for gestational age (Boyd et al., 2019; Hafekost, Lawrence, O’Leary, Bower, O’Donnell, et al., 2017; Högberg et al., 2019; Kalland et al., 2006; Putnam-Hornstein & Needell, 2011; Van Horne et al., 2018), birth defects (Van Horne et al., 2015, 2018), and diagnosis of ASD (McDonnell et al., 2019), Down Syndrome (Van Horne et al., 2018), or disabilities (e.g. intellectual disability) (Maclean et al., 2017). Family-level factors examined included family socioeconomic status (Austin et al., 2018; Cant et al., 2019; Coulton et al., 2016; Segal et al., 2019), birth order/number of children in the family (Austin et al., 2018; Högberg et al., 2019; Parrish et al., 2011; Parrish & Gessner, 2010; Putnam-Hornstein et al., 2011; Putnam-Hornstein & Needell, 2011; Van Horne et al., 2018), and previous experience/reports of maltreatment for the focal child (Eastman et al., 2016; Papalia et al., 2017; Putnam-Hornstein, 2011; Putnam-Hornstein, Cleves, Licht, & Needell, 2013) or their siblings (Eastman et al., 2016; Wilson et al., 2015). Finally, several studies examined societal and environmental risk factors, including housing conditions (e.g. poor quality housing and overcrowding) (Cant et al., 2019; Coulton et al., 2016) and neighbourhood economic impoverishment (Van Horne et al., 2015, 2018).

Authors of included studies perceived many advantages of data linkage as an approach to study risk factors for child maltreatment. As with the studies in the previous section, many studies concerning risk factors referenced the benefits of large, population-based samples (e.g. Ekéus et al., 2004; Hafekost, Lawrence, O’Leary, Bower, O’Donnell, et al., 2017; Högberg, Andersson, et al., 2018; Högberg, Lampa, et al., 2018). In terms of the benefits of the source data, one of the most-cited advantages was that it reduced many of the biases common to other methods (e.g. recall and sampling biases) (e.g. Ekéus et al., 2004; M. J. Green et al., 2018; Hafekost, Lawrence, O’Leary, Bower, O’Donnell, et al., 2017; Hafekost, Lawrence, O’Leary, Bower, Semmens, et al., 2017). Regarding the specific benefits of data *linkage*, authors noted that linking multiple administrative datasets facilitated study of a wider variety of risk factors than would have been possible using data from a single source (e.g. CPS records) (e.g. Putnam-Hornstein et al., 2011). Data linkage between children and their parents was seen as particularly advantageous, as it allowed for the study of family-level risk factors (e.g. Cram et al., 2015). Authors also noted advantages of data linkage over other methods; for example, linkage of population-level data facilitated large and representative samples that allowed authors to address some of the issues related to studying rare events, particularly when exposures and outcomes were found in different datasets (e.g. Putnam-Hornstein, 2011). Finally, authors saw data linkage as a valuable tool for tracking variations in the prevalence of risk factors over time (e.g. Putnam-Hornstein et al., 2011), which they viewed as critical for supporting the public health response to maltreatment.

*3. Outcomes for those who have experienced child maltreatment:* Studies examined three domains of outcomes associated with experience of child maltreatment: mental health, physical health, and education/employment (described in detail below). Again, common themes in terms of the perceived benefits of a data linkage approach were that it was population-based (e.g. Hu et al., 2017; Jackisch et al., 2019; Matheson et al., 2017; M. O’Donnell et al., 2010); included objective measures of relevant variables (e.g. Leslie et al., 2000; Matheson et al., 2017; Patton et al., 2019); and avoided common biases such as recall, social desirability, and sampling biases (e.g. Abajobir et al., 2017; Cutajar et al., 2010b; M. J. Green, Tzoumakis, et al., 2019; Hu et al., 2017; Jackisch et al., 2019). Additional benefits that were primarily related to the source data included the ability to reduce participant burden (e.g. Cutajar et al., 2010a; Jackisch et al., 2019; M. O’Donnell et al., 2010), establish temporality using prospectively-collected data (e.g. M. J. Green, Tzoumakis, et al., 2019; Hu et al., 2017), and study rare outcomes with sufficient statistical power (e.g. Cutajar et al., 2010b; Spataro et al., 2004). In terms of the perceived advantages of data linkage specifically, authors described that linking data across sectors reduced the possibility of confounding by offering a wide range of possible covariates for which to adjust (e.g. Hu et al., 2017; Kisely et al., 2018; Lanier et al., 2010; Rossen et al., 2019). As with the study of risk factors, authors also appreciated the ability to link children’s data to that of their parents, as it allowed for the child to be contextualised within their family and wider social environment (e.g. Patton et al., 2019).

#### Mental health and related outcomes:

Several studies examined mental health outcomes associated with child maltreatment. For children and adolescents (up to 18 years), outcomes studied included any mental health diagnosis during childhood and adolescence as well as specific diagnosis of self-harm, conduct disorders, and post-traumatic stress disorder (Cutajar et al., 2010a; M. J. Green, Tzoumakis, et al., 2019). A number of studies also examined the relationship between maltreatment and childhood outpatient mental health treatment (M. J. Green, Tzoumakis, et al., 2019; Leslie et al., 2000) and specifically presentations and admissions for self-harm and suicide-related behaviour during childhood and adolescence (Hu et al., 2017; Rhodes et al., 2012, 2013). For young adults (average age approx. 21 years), outcomes studied included depression and anxiety disorders (Dahl et al., 2017; Scott et al., 2012), attentional problems (Boyd et al., 2019), internalising and externalising behaviours (Kisely et al., 2018; Scott et al., 2010), post-traumatic stress disorder (Scott et al., 2010), alcohol or substance use disorders (Kisely, Mills, et al., 2020; Scott et al., 2010, 2012), and low quality of life (Abajobir, Kisely, Williams, Strathearn, Clavarino, & Najman, 2017). In later adulthood, outcomes studied included schizophrenia and psychotic disorders (Cutajar et al., 2010a, 2010b; Morgan et al., 2019; Spataro et al., 2004).

#### Physical health outcomes:

In terms of physical health, included studies examined associations between child maltreatment and adolescent smoking (Kisely, Abajobir, et al., 2020; T. L. Lewis et al., 2011), cardio-respiratory disease (Lanier et al., 2010), asthma (Lanier et al., 2010), high dietary fat intake (Abajobir, Kisely, Williams, Strathearn, & Najman, 2017), non-sexually transmitted infectious disease (Lanier et al., 2010), and premature mortality (Jackisch et al., 2019). Two studies examined physical health more generally, focusing on the association of child maltreatment and hospital admissions (Melissa O’Donnell et al., 2010) and healthcare costs (Patton et al., 2019). One study examined risk of teen parenthood for children who had experienced maltreatment (Font & Maguire-Jack, 2020).

#### Educational and employment outcomes:

Several studies focused on educational outcomes for those who had experienced child maltreatment. Childhood outcomes included developmental vulnerability (including five domains: poor social competency, poor prosocial/helping behaviour, anxious/fearful behaviour, aggressive behaviour, hyperactivity/inattention) (M. J. Green et al., 2018; Matheson et al., 2017; Rossen et al., 2019) and primary school experiences of school adjustment, school readiness, academic achievement, behaviour, retention, attendance, and special education status (Coulton et al., 2016; Galos, 2018; Laurens et al., 2020; Maclean et al., 2016; Ryan et al., 2018; Weiss & Fantuzzo, 2001). Two studies examined outcomes for secondary school students, one of which focused on absenteeism during exam days (Wong et al., 2017) and the other on school completion (Font & Maguire-Jack, 2020). Only one study examined the impact of maltreatment on employment and earnings in young adulthood (Font & Maguire-Jack, 2020).

*4. Intergenerational transmission of child maltreatment:* A small number of studies explored the likelihood of maltreatment among children whose parent(s) was/were maltreated. Two studies sought to quantify the overall probability of intergenerational transmission (measured as the proportion of parents who were listed as the victim in a child protection report who were later listed as the perpetrator in a child’s report) (Font et al., 2020; Galos, 2018). Of these, one study (Font et al., 2020) examined four distinct types of perpetration, comparing transmission rates across three groups with differential CPS involvement. Two additional studies had a more specific focus of quantifying the risk of intergenerational transmission amongst young mothers (Eastman & Putnam-Hornstein, 2019; Putnam-Hornstein et al., 2015).

Again, these studies referred to some of the common advantages listed in other subsections, such as large, population-based samples (e.g. Galos, 2018; Putnam-Hornstein et al., 2015) and inclusion of multiple maltreatment-related variables (e.g. Galos, 2018). In terms of the unique advantages of data linkage for studying intergenerational maltreatment, authors of included studies noted the ability to link data between children and parents, allowing for follow-up over multiple generations without disadvantages of recall bias (e.g. Eastman & Putnam-Hornstein, 2019; Font et al., 2020; Galos, 2018; Putnam-Hornstein et al., 2015). Furthermore, in terms of the study of young mothers, authors also noted that linked data allowed for the study of a rare event (teenage pregnancy) in a vulnerable population that may not typically participate in research (e.g. Eastman & Putnam-Hornstein, 2019). Finally, the longitudinal nature of administrative data was seen as a benefit as it reduced the time burden that would otherwise be required to follow up multiple generations (e.g. Galos, 2018).

*5. Predictive modelling:* Four studies used linked data to explore the feasibility of using predictive modelling to identify cases of child maltreatment (Vaithianathan et al., 2013; Wilson et al., 2015), maltreatment-related mortality (Parrish & Gessner, 2010), and placement in out-of-home care due to maltreatment (M. J. Green, Kariuki, et al., 2019). Studies varied in their approach to modelling. The number of variables included in the final models ranged from 6 (M. J. Green, Kariuki, et al., 2019; Parrish & Gessner, 2010) to 132 (Vaithianathan et al., 2013), the most common of which pertained to maternal characteristics (e.g. age at birth, marital status, mental health disorder, smoking/substance use in pregnancy). Where reported, Area Under the ROC Curve (AUC) values were lower for general maltreatment (range 0.76–0.88 (Vaithianathan et al., 2013; Wilson et al., 2015)) than for placement in out-of-home care (0.95 (M. J. Green, Kariuki, et al., 2019)). Positive predictive values were reported in only two studies, but ranged from 30% (child maltreatment (Wilson et al., 2015)) to 74% (out-of-home care placement (M. J. Green, Kariuki, et al., 2019)). All studies used de-identified data (i.e. did not identify specific individuals), and we did not find evidence that any of the four models had been evaluated for real-world use.

The main rationale the authors provided for using linked data concerned its potential to more accurately predict maltreatment-related outcomes (e.g. M. J. Green, Kariuki, et al., 2019; Parrish & Gessner, 2010). Specifically, by linking data from multiple agencies, they were able to consider a more comprehensive set of potential risk and protective factors than if they had relied on a single dataset (e.g. M. J. Green, Kariuki, et al., 2019). Furthermore, all studies highlighted potential applications of predictive modelling using data linkage, such as informing clinical protocols for decision-making/triage systems and strategies for targeting early intervention efforts. However, it is important to note that these statements were largely aspirational: there are several criteria to consider in the practical application of prediction models, and as mentioned above, none of the included studies had yet attempted to use their model in practice.

*6. Intervention and service evaluation:* Very few studies investigated services or interventions designed to prevent or respond to child maltreatment, of which only three were experimental or quasi-experimental in nature (including one randomised controlled trial) (Bruns et al., 2012; B. L. Green et al., 2017; Hong & Piescher, 2012). Compared with the other categories in this review, a greater proportion of studies in this category obtained active consent for linkage; however, some used de-identified records with implied exemption from consent requirements (e.g. Hong & Piescher, 2012; Maguire-Jack et al., 2019).

Included studies evaluated a wide variety of interventions and services. One study examined decision-making in CPS agencies, particularly in terms of how individual and county-level characteristics influenced whether investigations resulted in substantiations or out-of-home care placements (Maguire-Jack et al., 2019). The rest of the included studies examined outcomes associated with particular interventions. Preventative interventions studied included home visiting programmes (B. L. Green et al., 2017; Lanier & Jonson-Reid, 2014Lanier & Jonson‐Reid, 2014; Murphey & Braner, 2000), Parent-Child Interaction Therapy (Lanier et al., 2014), and family supportive housing (Hong & Piescher, 2012). Other more ‘down-stream’ interventions and services included family drug treatment court (Bruns et al., 2012), cash benefits for families whose children were removed due to maltreatment (Lee et al., 2017), and treatment foster care (Larson, 2010).

Two studies included a specific rationale for using data linkage as an evaluation tool, the first of which used linkage in order to improve case ascertainment (Maguire-Jack et al., 2019) and the second to better understand the needs of children across multiple agencies (Larson, 2010). Other studies listed advantages of administrative data more generally, for example that it provides objective outcome measures (e.g. Lanier et al., 2014) and enables the study of policy-relevant outcomes (e.g. B. L. Green et al., 2017; Murphey & Braner, 2000).

*7. Multi-sector involvement in those who have experienced child maltreatment:* Linkage of multi-agency data also generated a picture of involvement across services, which can help to understand the diverse needs of children who experience maltreatment and the effectiveness of the multi-agency care pathways that support them. Several studies focused on the experiences of ‘dual system youth’, that is, those involved with both child protection and justice services (Eastman et al., 2019; Herz et al., 2019; Hurren et al., 2017a). Other studies described the experiences of youth involved in child protection and homelessness services (Putnam-Hornstein et al., 2017; Rodriguez & Shinn, 2016). One study examined involvement across all three of these sectors (child protection, justice, and housing support) and quantified the likelihood of involvement in multiple sectors (Aalders, 2012).

These studies relied on linked data to produce an accurate picture of multi-sector involvement without having to rely on self-report (e.g. Eastman et al., 2019; Putnam-Hornstein et al., 2017) and to do so on a population level (e.g. Eastman et al., 2019; Putnam-Hornstein et al., 2017). Two studies also referred to the fact that data linkage is a relatively feasible and time- and cost-efficient way of studying multi-sector involvement (Eastman et al., 2019; Hurren et al., 2017a). Finally, studies commonly indicated practical applications of the methodology, for example to better understand service ‘touch points’ and identify strategic points for intervention (e.g. Eastman et al., 2019; Herz et al., 2019; Putnam-Hornstein et al., 2017; Rodriguez & Shinn, 2016).

*8. Methodological considerations and advancements:* Another theme across studies was methodological considerations and advancements related to using data linkage to study child maltreatment. Some studies focused more broadly on how data linkage can improve our understanding of and response to maltreatment, for example, how the combination of multiple administrative data sources improved detection in comparison with one dataset alone (Putnam-Hornstein et al., 2011; Schnitzer et al., 2008). Others provided more technical considerations, for example, regarding the accuracy and utility of ICD codes in identifying maltreatment (Raghavan et al., 2015) or the differences in prospectively-versus retrospectively-ascertained maltreatment and their effects on outcomes of interest (Galos, 2018; Scott et al., 2012). Two studies aimed to quantify the effects of misclassification and other types of bias within data linkage (Galos, 2018; Parrish et al., 2017), demonstrating the importance of linkage validation and quality assessment.

## Discussion

A total of 121 studies met our inclusion criteria of studying child maltreatment by linking data from at least two different data-holding organisations (of which at least one was longitudinal and administrative in nature) for the same individual. The vast majority of included studies were published in the past decade and conducted in the United States or Australia. Below we discuss findings in relation to each of our objectives.

### Objectives 1 and 2. Describe which data (from which sectors) have been linked and overview linkage processes

Most datasets came from social services or health, with fewer from justice, education, or other sources. Linking data across diverse sectors facilitates a more complete picture of child maltreatment, which provides many benefits in terms of mapping risk factors and outcomes across domains (Belsky, 1993) and understanding when and where individuals who have experienced maltreatment access services. As may be expected, different agencies used different operationalisations and indicators of maltreatment, the standardisation of which merits future consideration.

Overall, many studies did not report sufficient detail relating to data linkage processes (including consent procedures, quality of linkage, risk of bias, and technical considerations) to align with current guidance (e.g. Benchimol et al., 2015; Gilbert et al., 2018), which is consistent with the findings from Chikwava and colleagues’ recent review (2021). Understanding data quality is particularly important in assessing the value of data linkage as a method of studying child maltreatment, and the inadequate reporting of linkage processes make it difficult to assess the quality and potential biases of linkages and therefore the robustness of study conclusions. These issues were the focus of a number of the studies included in the ‘methodological advancements’ category. For example, Raghavan and colleagues’ (2015) study on the quality of administrative codes used to indicate maltreatment provided interesting insight into the utility and limitations of using medical codes to ascertain child maltreatment and of the biases related to these codes. Other studies in the review examined the impact of different technical or analytical decisions (Parrish et al., 2017), which may be helpful in improving the accuracy of estimates derived from linkages.

### Objectives 3 and 4. Identify the main purposes/uses of data linkage in the context of child maltreatment and overview the types of questions being addressed using data linkage

Below we discuss included studies’ main purposes and findings in relation to Putnam-Hornstein and colleagues’ (2011) public health framework, which conceptualises the potential usages of data linkage within the field of child maltreatment. The framework consists of four ‘steps’ that build upon each other with the overall aim of reducing the prevalence, incidence, and impacts of maltreatment. These are (1) defining the problem through data collection/surveillance, (2) identifying risk and protective factors, (3) developing and testing interventions through efficacy/effectiveness research, and (4) implementing and monitoring interventions. The long-term and ‘quasi-prospective’ nature of administrative data and the holistic, multi-sectoral view made possible through data linkage are ideal for addressing these four areas.

*Surveillance:* The studies in this review demonstrated the benefits of data linkage for providing estimates of overall population prevalence and incidence of child maltreatment as well as enabling estimation for specific vulnerable groups (e.g. young mothers and homeless youth). Derived from whole-of-population data, these estimates can serve as the foundation for designing policy and intervention strategies by indicating the scope of maltreatment in the general population and highlighting groups that may benefit from more targeted intervention (Putnam-Hornstein et al., 2011). However, what remains to be determined is the accuracy of these estimates in relation to those derived from studies not using data linkage methods (e.g. those relying on case note review, self-report surveys, etc.). Whilst this review demonstrated that data linkage improves case ascertainment over single datasets and is a feasible way to obtain population-level estimates (e.g. Schnitzer et al., 2008), the extent to which it captures the *true prevalence* of maltreatment is still unclear, especially given the lack of consideration of the linkage quality and the possible biases this may introduce. This is an important consideration that should be explored in the future, especially given the known risk of non-inclusion exacerbating inequality (Ibrahim et al., 2021; Knight et al., 2021). Furthermore, the parameters remain to be clarified by which linked administrative data might be provided back to the services that provide individual datasets, and the ways data might be used to inform decisions about individuals who may be at particular risk.

*Risk and protective factors: *The studies included in our review also highlighted the ability of data linkage to further our understanding of risk factors and outcomes related to child maltreatment. Included studies examined risk factors and outcomes spanning all levels of Belsky’s (1993) developmental-ecological model and across many sectors (including child protection, social and housing services, health, education, and justice), which reflects the multi-faceted nature of maltreatment and its consequences. Perceived advantages of using a data linkage approach included large sample sizes, population-based data, ability to study a wide range of cross-sector risk factors, reduced biases (e.g. recall and selection bias), and the ability to track risk factors over time at a population-level. Studies also noted the power of data linkage for contextualising the child within their family. Many of the included studies examined risk factors through familial linkages (e.g. children to parents and children to siblings), which allow for exploration of risk factors that might be otherwise difficult to study. In addition to facilitating study of intergenerational transmission of maltreatment, linkage between family members adds important information about harmful events or actions available only in the parental or sibling records and provides information about the family that can inform service support and preventative interventions at a family level.

However, there were also gaps in the literature in terms of risk and protective factors. For example, whilst there is significant potential for using linkage of administrative data and research data (e.g. from cohort studies) to unravel the complex aetiology of maltreatment, only a handful of studies in the review included such linkage. Furthermore, none of the included studies described using unstructured (free-text) administrative data, even though these are recognised as a key source of information on risk and protective factors. In the United Kingdom, for example, it is estimated that approximately 70% of the information relating to adverse events is recorded in free text fields as unstructured data (Downs, 2017), and analysis requires complex natural language capability – a facility that is resource- and time-intensive, and not available to most research teams. Improving access to these resources could help ensure we are maximising the utility of administrative data.

*Intervention development, testing, and implementation:* Relatively few studies focused on evaluating prevention strategies or interventions for children who have already experienced maltreatment. Interventions for child maltreatment – particularly when they are preventative – are often difficult to evaluate in general. Trials in the field often suffer from poor methodological reporting, short-term follow-ups, inappropriate analyses, and lack of replication, so there is still much to be learnt about the effectiveness of such interventions (MacMillan et al., 2009). Whilst some of the included studies mentioned advantages of using data linkage in this way (e.g. the ability to study objectively-recorded, policy-relevant outcomes), the potential of data linkage to help address this gap remains largely unclear and merits further exploration. 

No studies in this review used data linkage as a ‘real world' means of identifying individuals who may benefit from particular interventions/services or to randomise individuals to a particular intervention. In theory, data linkage may make it possible to examine a large known proportion of children who are maltreated, detect patterns in risk factors, and follow-up those identified as being at high-risk of maltreatment. Critically, any programme aimed at identifying children at-risk of or experiencing maltreatment would need to meet strict requirements related to effectiveness (including potential harms), feasibility, and acceptability, such as those set out by the UK National Screening Committee (UK National Screening Committee, 2015). Specific criteria include model accuracy and predictive ability of included variables (Leventhal, 1988), bias within the original data and bias due to the linkage, explicability of the system and design of the model, and understandability and acceptability to stakeholders (Joshi & Morley, 2019). Although one study in the review (Wilson et al., 2015) met established criteria for accuracy, none appeared to meet all relevant criteria for real-world case identification. However, given that study authors were hopeful that their models could eventually be used in practice, it is clear that this is an important area for careful study and consideration.

### Limitations

We acknowledge several limitations for this review. First, as we included only studies using data linkage, we were unable to directly compare findings from data linkage studies with findings from studies using other approaches. Thus, we are unable to systematically determine what *unique* information data linkage studies have contributed to our understanding of maltreatment. Second, in terms of our definition of maltreatment, we excluded studies that focused *only* on children in out-of-home care placements*.* Although there is a large overlap in these populations (i.e. many children who are placed in out-of-home care will have experienced maltreatment), there is also a significant proportion of children in out-of-home care placements for other reasons, and thus including all children in out-of-home care would not provide information specific to *presentations* of maltreatment. Furthermore, we did not include prenatal neglect (including neonatal abstinence syndrome or foetal alcohol syndrome) in our definition of maltreatment. Third, whilst we were broad in our search strategy, it is possible that we may have missed important studies. For example, if studies did not mention data linkage within their title/abstract, they would not have come up in our searches (although we note that this is an issue for all reviews relating to data linkage). Additionally, certain study types, such as service evaluations, are often not systematically searchable. We attempted to address both of these issues through extensive manual searching, but it is likely that there are additional studies we have not found. We were additionally concerned we may have missed studies from the Nordic countries (given their high-quality register data), which we attempted to address through consultation with a Swedish researcher in the field; however, as this was a *post-hoc* conversation, we ran only targeted searches in two databases, rather than re-running our entire search strategy a third time. Finally, whilst not a limitation per se, we would like to emphasise that the conclusions of this review relate to the findings of *data linkage studies,* and as such, one should not draw overall conclusions about the epidemiology of child maltreatment or effectiveness of related services or interventions without considering findings from the broader child maltreatment literature.

### Recommendations for policy and future research

Accurate, accessible information on the prevalence, aetiology, and consequences of child maltreatment is the foundation for designing effective policy and interventions to address it. This review has begun to demonstrate the range of ways in which data linkage can contribute to building these strong foundations. However, there is still much research that is needed to determine how data linkage can best contribute to the public health response to maltreatment.

First, as described in the previous section, the review highlighted a significant gap in terms of methodological reporting. Despite the existence of established guidance for reporting on linkage techniques and methods (including assessment of linkage quality) (Benchimol et al., 2015; Gilbert et al., 2018), the vast majority of studies included no or insufficient information in these areas. This may be due in part to unavailability of information, a common consequence of the fragmentation of data processing (Gilbert et al., 2018; Harron et al., 2017). Data providers, linkers, and analysts can begin to address this issue through clear communication about each step of the linkage process (Gilbert et al., 2018). Furthermore, even in the absence of access to identifiable data, there are established methods for evaluating linkage quality, including through post-linkage validation, sensitivity analysis, and comparison of linked and unlinked data (Harron et al., 2017). More detailed description of linkage processes is important for improving transparency and reproducibility, and until we can properly assess data and linkage quality for studies in this area, it will be difficult to assess the robustness of their findings and conclusions.

Second, as stated in the *Limitations* section, this review was not designed to systematically determine what *unique* information data linkage adds beyond what is already known. To build on this review, we suggest that future systematic reviews directly compare findings from studies using data linkage with the best available evidence to understand the unique contribution of data linkage studies. Such comparison could help determine whether data linkage produces knowledge not already known from other studies, or, if not, whether the practical benefits relative to other methods (e.g. cost savings and convenience) still support its use in certain circumstances. These comparisons should further aim to evaluate the sensitivity and specificity linked data for ascertaining cases of child maltreatment, as this will directly influence its usefulness as a tool for practice and research.

Third, the review highlighted a clear gap in terms of the possible uses for linked data suggested in Putnam-Hornstein and colleagues’ (2011) public health framework. Studies clustered primarily in the ‘discovery’ end of the framework (defining the problem and identifying risk and protective factors), with very few focused on evaluating or implementing interventions. Only two studies (Gilbert et al., 2012; Högberg, Lampa, et al., 2018) measured maltreatment in relation to macro-level policy initiatives or changes in medical guidelines, despite this being an area where population-level data on time trends and geographical patterning could be particularly useful (Prinz, 2017). That data linkage has not often been used for evaluation indicates a significant missed opportunity. This is particularly true for the United Kingdom, where there has been emphasis moving towards delivering care as an integrated system, including through the creation of linked records between health and social services that can be de-identified to use for research. In fact, legislation expected in 2022 will make it a requirement to collaborate between health and social care to provide services (Department of Health and Social Care, 2021). Methods of evaluating these novel multi-agency interventions and care pathways will be critical, and data linkage could be an important method for supporting these endeavours.

Fourth, very few of the included studies linked administrative data with study-specific (research) data. This represents another missed opportunity, as research data (e.g. on genetics, biomarkers, and deep phenotyping) could complement what is available in administrative data and enable complex analyses that would not otherwise be possible. Whilst some studies (e.g. the UK Biobank and the Avon Longitudinal Study of Parents and Children) are starting to link cohort and administrative data, none of the studies using these data met inclusion criteria for this review, highlighting an area for additional development.

Finally, further consideration is needed regarding how to address the range of practical hurdles associated with establishing data linkage systems. As more and more countries attempt to introduce data linkage to enhance understanding and evaluation of risk and health, common challenges are emerging. For example, in the UK, there are many difficulties associated with access, linkage and use of social services data, one of the main sources of information pertaining to child maltreatment. These include uncertainty around the legalities and governance of sharing and linking data, data protection and privacy issues (Mourby et al., 2019), lack of technical infrastructure (Blackwell et al., 2015; Copeland, 2015), problems with interoperability (Copeland, 2015; Harron et al., 2017), and lack of human resources with relevant skills and knowledge (Ainsworth & Buchan, 2015). To benefit from the potential of linked data, concerted action is required to address these challenges. This is likely to include targeted capital investment aimed towards the access and use of social services data, linked to health and research data.

## Conclusions

There is increasing interest around data linkage as a tool for understanding, preventing, and mitigating the effects of child maltreatment. The studies included in this review demonstrated the wide variety of ways in which data linkage can be used to generate research evidence to contribute to public health policies for maltreatment, especially in terms of better understanding its aetiology and consequences. However, how research using linked data can be translated into effective service development and monitoring, or targeting of interventions, is underexplored in terms of privacy protection, ethics and governance, data quality, and evidence of effectiveness.

## Supplemental Material

sj-pdf-1-cmx-10.1177_10775595221079308 - Supplemental Material for Leveraging Administrative Data to Better Understand and Address Child Maltreatment: A Scoping Review of Data Linkage Studies Click here for additional data file. Supplemental Material, sj-pdf-1-cmx-10.1177_10775595221079308 for Leveraging Administrative Data to Better Understand and Address Child Maltreatment: A Scoping Review of Data Linkage Studies by Emma Soneson, Shruti Das, Anne-Marie Burn, Marije van Melle, Joanna K. Anderson, Mina Fazel, Peter Fonagy, Tamsin Ford, Ruth Gilbert, Katie Harron, Emma Howarth, Ayla Humphrey, Peter B. Jones, and Anna Moore in Child Maltreatment

sj-pdf-2-cmx-10.1177_10775595221079308 - Supplemental Material for Leveraging Administrative Data to Better Understand and Address Child Maltreatment: A Scoping Review of Data Linkage Studies Click here for additional data file. Supplemental Material, sj-pdf-2-cmx-10.1177_10775595221079308 for Leveraging Administrative Data to Better Understand and Address Child Maltreatment: A Scoping Review of Data Linkage Studies by Emma Soneson, Shruti Das, Anne-Marie Burn, Marije van Melle, Joanna K. Anderson, Mina Fazel, Peter Fonagy, Tamsin Ford, Ruth Gilbert, Katie Harron, Emma Howarth, Ayla Humphrey, Peter B. Jones, and Anna Moore in Child Maltreatment
