# Supplementary material for: Leveraging Administrative Data to Better Understand and Address Child Maltreatment: A Scoping Review of Data Linkage Studies
Source: Child Maltreat. 2022 Mar 3;28(1):176–95. doi: 10.1177/10775595221079308 (PMC9806482; doi:10.1177/10775595221079308)
Supplement: sj-pdf-1-cmx-10.1177_10775595221079308 - Supplemental Material for Leveraging Administrative Data to Better Understand and Address Child Maltreatment: A Scoping Review of Data Linkage Studies [file sj-pdf-1-cmx-10.1177_10775595221079308.pdf]

Supplementary Table 1. Search strategies by database

MEDLINE

Data linkage	Children and young people
data adj1 link* OR record* adj1 link* OR data adj1 match* OR “administrative data cohort” OR electronic adj2 cohort OR “longitudinal link*”<br><br>OR Medical Record Linkage/	Child* OR “young person” OR “young people” OR youth OR adolescen* OR teenage* OR juvenile* OR toddler* OR infant OR infants OR infancy OR student* OR pupil*<br><br>OR adolescent/ OR child/ OR child, preschool/ OR infant/ OR students/

Embase

Data linkage	Children and young people
data adj1 link* OR record* adj1 link* OR data adj1 match* OR “administrative data cohort” OR electronic adj2 cohort OR “longitudinal link*”<br><br>OR Medical Record Linkage/	Child* OR “young person” OR “young people” OR youth OR adolescen* OR teenage* OR juvenile* OR toddler* OR infant OR infants OR infancy OR student* OR pupil*<br><br>OR adolescent/ OR child/ OR preschool child OR infant/ OR student/

PsycINFO

Data linkage	Children and young people
data N1 link* OR record* N1 link* OR data N1 match* OR “administrative data cohort” OR electronic N2 cohort OR “longitudinal link*”	Child* OR “young person” OR “young people” OR youth OR adolescen* OR teenage* OR juvenile* OR toddler* OR infant OR infants OR infancy OR student* OR pupil*

CINAHL

Data linkage	Children and young people
data N1 link* OR record* N1 link* OR data N1 match* OR “administrative data cohort” OR electronic N2 cohort OR “longitudinal link*”	Child* OR “young person” OR “young people” OR youth OR adolescen* OR teenage* OR juvenile* OR toddler* OR infant OR infants OR infancy OR student* OR pupil*<br><br>OR (MH "Child, Preschool") OR (MH "Child") OR (MH "Infant") OR (MH "Adolescence") OR (MH “Students”)

ERIC

Data linkage	Children and young people
data N1 link* OR record* N1 link* OR data N1 match* OR “administrative data cohort” OR electronic N2 cohort OR “longitudinal link*”	Child* OR “young person” OR “young people” OR youth OR adolescen* OR teenage* OR juvenile* OR toddler* OR infant OR infants OR infancy OR student*

	OR pupil*<br><br>OR DE "Adolescents" OR DE "Children"<br>OR DE "Preadolescents" OR DE "Youth"<br>OR DE "Infants" OR DE "Young Children"<br>OR DE "Toddlers" OR DE “Students”
