# Supplementary material for: Leveraging Administrative Data to Better Understand and Address Child Maltreatment: A Scoping Review of Data Linkage Studies
Source: Child Maltreat. 2022 Mar 3;28(1):176–95. doi: 10.1177/10775595221079308 (PMC9806482; doi:10.1177/10775595221079308)
Supplement: sj-pdf-2-cmx-10.1177_10775595221079308 - Supplemental Material for Leveraging Administrative Data to Better Understand and Address Child Maltreatment: A Scoping Review of Data Linkage Studies [file sj-pdf-2-cmx-10.1177_10775595221079308.pdf]

Supplementary Table 2. Characteristics of included studies

| Citation (Year)<br><br>Country/ies | Purpose of linkage<br><br>Study aims<br><br>Stated outcome(s) of interest | Description of datasets<br><br>1. Data sources<br>2. Includes study-specific (research) data?<br>3. Population-based?<br>4. Data linked across generations? (e.g. mother-infant records linked)<br>5. Years of age included in the dataset | Description of linkage process<br><br>e.g. deterministic vs. probabilistic linkage & linkage variables; retrospective linkage vs. prospective (or 'living') linkage; linkage validation/ quality assessment; consent | Population/cohort characteristics | Types of maltreatment studied or study definition of maltreatment [study exposure or outcome] | Summary of study findings (related to child maltreatment) |
|---|---|---|---|---|---|---|
| **Descriptive epidemiology** | | | | | | |
| Fisher (2019)<br><br>USA | **Purpose:** Descriptive<br><br>**Aims:** To examine rates of maltreatment referrals, screening for further action, and substantiated maltreatment for children with versus without autism spectrum disorder<br><br>**Outcomes:** Maltreatment referrals, screening for further action, substantiated maltreatment | 1. **Health** (birth vital records, Autism and Developmental Disability Monitoring network records); **social services** (Tennessee Department of Children's Services (CPS records))<br>2. No<br>3. Yes<br>4. No<br>5. 0-10 years | Retrospective, one-time linkage using deterministic linkage technique. Linked on common identifiers (e.g. name, address, birth date). Linkage validation/quality assessment ND. | N = 24,306 children from within the Tennessee Autism and Developmental Disability Monitoring network surveillance area (N = 387 children with autism spectrum disorder and N = 23,919 without). 17.3% of children with autism spectrum disorder and 7.4% without had a maltreatment referral; 10.6% and 6.8%, respectively, had a screened in referral; 3.9% and 3.4%, respectively, had a substantiated referral. | All maltreatment referrals, referrals 'screened in' for further action, and substantiated referrals via the Tennessee Child Abuse Hotline [Outcome] | Relative to the entire control population, children with autism spectrum disorder were significantly more likely than those without to have any maltreatment referral (OR = 2.63, 95% CI 2.00, 3.42) and have a screened in referral (OR = 1.68, 95% CI 1.20, 2.30), but not significantly more likely to have a substantiated referral. Relative to all referrals to the Tennessee Child Abuse Hotline, referrals for children with autism spectrum disorder were more likely to be screened out for further action (OR = 0.15, 95% CI 0.09, 0.26). Relative to screened in referrals, children with autism spectrum disorder were equally as likely as children without to have the referral substantiated (p=0.055). Substantiation was less common for boys with autism spectrum disorder than girls with autism spectrum disorder (1.9% vs. 13.6%; OR = 0.12, 95% CI 0.04, 0.36). There were no significant differences in substantiations for children with autism spectrum |

| | | | | | | |
|---|---|---|---|---|---|---|
| | | | | | | disorder in terms of race/ethnicity or IQ. |
| Gessner (2004)<br><br>USA | **Purpose:** Descriptive<br><br>**Aims:** To determine the incidence of and risk factors associated with infant physical abuse in Alaska<br><br>**Outcomes:** (1) Physical abuse and (2) physical abuse resulting in hospitalisation or death | 1. **Health** (Alaska Maternal and Infant Mortality Review, Alaska Trauma Registry (hospital-based trauma registry), Hospital discharge database, vital statistics); **social services** (Alaska Division of Family and Youth Services (CPS) records)<br>2. No<br>3. Yes<br>4. Yes<br>5. 0-1 year | Retrospective, one-time linkage using deterministic linkage technique (exact matches). Matched on date of birth, name, village of residence, gender, CPS date of investigation, date of death/hospital admission. Implied exemption from consent requirements. Manual review to identify transcription | All births in Alaska from 1994-2000 (N =70,842). Maternal ethnicity: 65.7% White, 23.8% Alaska Native, 5.0% Asian/Pacific Islander, 4.4% African American. | Substantiated physical abuse before 1 year old (identified through CPS database) or infant death (identified through death certificates) [Outcome] | Incidence of all physical abuse was 4.6 per 1000 live births; incidence of physical abuse resulting in hospitalisation or death was 1.0 per 1000 live births (0.96 per 1000 for hospitalisation with/without death; 0.20 per 1000 for death with/without hospitalisation). Factors associated with the greatest population attributable risks were maternal or paternal education ≤12 years (31% and 24%, respectively), unmarried mother (29%), and maternal prenatal substance use (15%). The first four months were associated with the highest risk (under 4 months accounted for 41% of all physical abuse and 49% of |

| | | | | | | |
|---|---|---|---|---|---|---|
| | | | errors; 90% of abuse records matched to birth certificates (unmatched data did not differ from matched data on race, gender, age, hospitalisation, or death status). | | | hospitalisation/deaths). |

| | | | | | | |
|---|---|---|---|---|---|---|
| Gilbert (2012)<br><br>Sweden, USA, Canada, Australia, England, New Zealand (linkage in Canada and Australia only) | **Purpose:** Descriptive<br><br>**Aims:** To explore trends in six developed countries in three types of indicators of child maltreatment for children younger than 11 years, since the inception of modern child protection systems in the 1970s<br><br>**Outcomes:** Child maltreatment (physical abuse and neglect) | 1. **Health** (hospital admissions, mortality data), **social services** (CPS records)<br>2. No<br>Implied exemption from active consent.<br>3. Yes<br>4. ND<br>5. 0-11 years | Could not categorise due to insufficient description of linkage techniques (each country individually responsible for linkage). Linkage validation/quality assessment ND. | Whole of population data for children <11 years old living in Manitoba, Canada, or Western Australia. | Physical abuse and neglect before 11 years old, including indicators for deaths, maltreatment-related injury admissions, and CPS notifications, investigations, officially recognised maltreatment, and placements into out-of-home care [Outcome] | Western Australia: stable rates of violent deaths for infants and older children (> 1 year old). Stable rates of injury admissions due to maltreatment/assault in older children but significant increase in infants. For infants, most maltreatment indicators significantly increased since 1990s with significant increase in out-of-home care placements in early 1990s (smaller and not significant following this time period). For older children, no significant change in officially recognised maltreatment, but significant increase in notifications from early 1990s to mid 1990s, then decrease (accompanied by significant increase in out-of-home care placements).<br><br>Manitoba, Canada: significant decreases in violent deaths for infants and older children and significant decreases in injury admissions due to maltreatment/assault for infants (not significant in older group). Rates of out-of-home placements stable across ages.<br><br>Both: a large proportion of children who were admitted for a maltreatment-related injury also had CPS records (66% of children admitted in Western Australia and 44% in Manitoba had a CPS notification). Few children with CPS records had been admitted for an injury (3% of children with CPS notifications in Western Australia had a maltreatment-related injury admission and 21% had an admission for any injury; 1% of children in care in Manitoba had a maltreatment-related injury and 7% had an admission for any injury). Of the children who died from violence in |

| | | | | | | |
|---|---|---|---|---|---|---|
| | | | | | | Western Australia, 4% had a previous maltreatment-related injury admission and 19% had a CPS notification. |

| | | | | | | |
|---|---|---|---|---|---|---|
| Högberg (2018a)<br><br>Sweden | **Purpose:** Descriptive<br><br>**Aims:** To examine the incidence of diagnosis of infant abuse over three decades in Sweden<br><br>**Outcomes:** Infant abuse diagnosis or other diagnosis indicating possible maltreatment (subdural haemorrhage, skull fracture, cerebral contusion, convulsions, retinal haemorrhage, rib fracture, long bone fracture) before 1 year of age | 1. **Health** (Swedish Medical Birth Register, Swedish Patient Register (inpatient and outpatient), Swedish Cause of Death Register)<br>2. No<br>3. Yes<br>4. Yes<br>5. 0-1 year | Retrospective, one-time linkage using a deterministic linkage technique (within a system using national registries). Linked on personal identity number. Exempt from consent requirements. Linkage validation/quality assessment ND. | N = 2,984,813 live births in Sweden between 1987 and 2014, of which N = 182,974 had a diagnosis of abuse or diagnosis indicating possible maltreatment (comparison group N = 731,901 infants without a diagnosis) | ICD diagnosis of infant abuse (including observation for suspected abuse, battered baby syndrome, maltreatment syndrome, neglect and abandonment, or other maltreatment) as well as other diagnoses indicating possible maltreatment (subdural haemorrhage, skull fracture, cerebral contusion, convulsions, retinal haemorrhage, rib fracture, long bone fracture)<br>[Outcome] | N = 368 infants were diagnosed with maltreatment between 1987 and 2014 (12.3 per 100,000) with a fatality rate of 1.9%. Diagnosis increased tenfold during the period, slowly from 1996 and peaking from 2008-2014. Incidence doubled between the periods from 1997-2007 and 2008-2014 (from 12.0 to 26.5 per 100,000 infants). Subdural haemorrhage, rib fracture, retinal haemorrhage, and skull fracture were the primary drivers for diagnosis. During 2008-2014, cases diagnosed with infant abuse had significantly increased rate ratios for subdural haemorrhage, skull fracture, retinal haemorrhage, and long bone fractures. |
| Högberg (2018b)<br><br>Sweden | **Purpose:** Descriptive<br><br>**Aims:** To analyse subdural haemorrhage during infancy in Sweden by incidence, subdural haemorrhage category, diagnostic distribution, age, co-morbidity, mortality, and maternal and perinatal risk factors; and its association with accidents and diagnosis of abuse<br><br>**Outcomes:** Subdural | 1. **Health** (Swedish Medical Birth Register, Swedish Patient Register (inpatient and outpatient), Swedish Cause of Death Register)<br>2. No<br>3. Yes<br>4. Yes<br>5. 0-1 year | Retrospective, one-time linkage using a deterministic linkage technique (within a system using national registries). Linked on personal identity number. Exempt from consent requirements. Linkage validation/quality assessment ND. | N = 395,812 Swedish live births between 1997 and 2014 with any entry in the National Patient Register before age 1, N = 182,974 had one of 119 select diagnoses of interest (with a comparison group of N = 731,901 infants without a diagnosis) | ICD diagnoses of subdural haemorrhage and abuse (observation for suspected abuse, battered baby syndrome, maltreatment syndrome)<br>[Outcome] | N = 251 infants were diagnosed subdural haemorrhage *not related to birth injury* before age 1 (incidence was 7.5 per 100,000 for traumatic subdural haemorrhage, 5.2 per 100,000 for acute nontraumatic subdural haemorrhage, and 0.8 per 100,000 for both). N = 43 infants (14%) had a combination of abuse and subdural haemorrhage diagnoses (2.3 per 100,000). N = 5 infants died from accidents or abuse/homicide, two of whom did not have any prior diagnosis. 64.7% of infants with a diagnosis of subdural haemorrhage were boys ($p<0.001$). Several perinatal factors were related to subdural haemorrhage |

| | | | | | | |
|---|---|---|---|---|---|---|
| | haemorrhage; accidents and diagnosis of abuse | | | | | diagnosis. |
| Horn (2017)<br><br>USA | **Purpose:** Descriptive<br><br>**Aims:** To identify refugees in CPS data systems and explore the experiences of Somali and Oromo youth in Minnesota's child protection system<br><br>**Outcomes:** CPS involvement and experiences for Somali/Oromo youth | 1. Minn-LInK project: **education** (Minnesota Automated Reporting Student System (incl. refugee status data)); **social services** (Minnesota Department of Human Services Social Service Information System (incl. CPS records))<br>2. No<br>3. Yes<br>4. No<br>5. Early childhood - 12th grade (approx. 17-18 years) | Retrospective, one-time linkage using probabilistic linkage technique (from a larger research database performing multiple/updated linkages). Data were anonymised post-linkage. Linkage validation/quality assessment ND. | N = 691 Somali and Oromo youth refugees in Minnesota involved in CPS (3.7% of total study population). 46.6% female; 14.0% in early childhood and preschool, 29.4% in kindergarden-2nd grade, 24.9% in 3rd-5th grade, 15.8% in 6th-8th grade, 15.9% in 9th-12th grade; 91.0% Somali, 9.0% Oromo. 3.4% of Somali youth and 3.9% or Oromo youth had experience of CPS involvement; N = 634 accepted cases. N = 1187 total allegations (61.0% neglect, 32.8% physical abuse, 3.7% sexual abuse, 0.1% mental injury and emotional harm, 2.4% medical neglect). | CPS involvement defined as those with 1+ accepted case of neglect, physical abuse, sexual abuse, mental injury and emotional harm, and medical neglect. Additional variables collected included 1) involvement in an accepted case of child maltreatment, 2) CPS response, 3) maltreatment substantiation, 4) allegation type, 5) out-of-home placement, 6) out-of-home placement length, 7) out-of-home placement setting, and 8) caregiver strengths and needs [Outcome] | 3.7% of Somali and Oromo youth had CPS involvement. N = 1187 allegations of maltreatment and N = 634 accepted cases (most common allegations were neglect, and physical and sexual abuse). 64.8% of cases receiving a Family Investigation response resulted in at least one substantiation. 17% of youth with CPS involvement had at least one out-of-home placement: the most common placements for Somali youth were residential treatment centres (41.4%) and non-related foster families (33.1%) while the most common placements for Oromo youth were non-related foster families (36.8%) and juvenile correctional facilities (31.6%). Family strengths included low rates of substance use/other health issues; needs included social support, mental health/coping support, and parenting skills. |

| | | | | | | |
|---|---|---|---|---|---|---|
| Maclean (2017a)<br><br>Australia | **Purpose:** Descriptive<br><br>**Aims:** To report the prevalence of different disabilities within the child protection system in an Australian state, and to assess risk of maltreatment in various types of disability taking into account child, family, and neighbourhood risk factors<br><br>**Outcomes:** Disabilities within CPS and risk of maltreatment by disability type | 1. Western Australian Data Linkage System: **health** (WA Register of Developmental Anomalies, Hospital Morbidity Data System, Intellectual Disability Exploring Answers (IDEA), Mental Health Information System, Midwives Notification System, Birth Registrations, Mortality Database); **social services** (Department of Child Protection and Family Support (CPS) records)<br>2. No<br>3. Yes<br>4. Yes<br>5. 0-adolescence | Near real-time ('living') linkage from multiple updates using probabilistic linkage technique. Linked on medical record number, first and last name, initial, date of birth, sex, and addresses. Implied exemption from consent requirements. Data were shared with researchers in de-identified format. Linkage quality assessed through clerical review of a sub-sample of matches; very low (~0.1%) false positive and false negative matches. | All children born in Western Australia 1990-2010 (N = 524,534). 48.8% female; 6.0% Aboriginal. 4.6% had a maltreatment allegation and 2.2% had a substantiated allegation. | Allegations and substantiations of maltreatment (physical abuse, sexual abuse, emotional abuse, and neglect) [Outcome] | 25.9% of maltreatment allegations (adj. HR compared with children with no disability = 1.74, 95% CI 1.68, 1.80) and 29.0% of substantiations (adj. HR = 1.89, 95% CI 1.80, 1.98) involved children with a disability. Children with mental/behavioural disorders made up 15.6% of all allegations, 6.7% for intellectual disability 6.6% for birth defects/cerebral palsy, 4.5% for conduct disorder, and <1% each for Down's Syndrome and autism, with similar patterns for substantiations. Type of maltreatment was similar across disability types (~25% neglect, ~24% physical abuse, ~19% sexual abuse, ~3.5% emotional abuse), though children with intellectual disabilities had a higher proportion of neglect, and those with conduct disorder had a higher proportion of physical abuse. The highest risk for maltreatment (compared with children with no disability) was that of children with intellectual disabilities (adj. HR for any maltreatment allegation = 2.14, 95% CI 2.00, 2.28), followed by conduct disorder (adj. HR = 1.84, 95% CI 1.70, 1.89) and mental and behavioural disorders (adj. HR = 1.62, 95% CI 1.55, 1.69). Children with autism had significantly lower risk than those without any disability. There was no significant difference in risk for alleged maltreatment for those with Down's Syndrome or birth defects/Cerebral Palsy. |

| | | | | | | |
|---|---|---|---|---|---|---|
| Parrish (2020)<br><br>USA | **Purpose:** Descriptive<br><br>**Aims:** To calculate unadj. and race-standardised cumulative incidence to first CPS contact before age 7<br><br>**Outcomes:** CPS contact before age 7 | 1. Alaska Longitudinal Child Abuse and Neglect Linkage (ALCANLink): **health** (birth and death records); **social services** (CPS records)<br>2. No<br>3. Yes<br>4. No<br>5. 0-7 years | Multiple one-time linkages using probabilistic linkage technique. Alaska records linked on first and last name, date of birth, and sex; California records linked on first name, last name, date of birth, residential address, and guardian/parent names and birthdates. Linkage processes were assessed (no further detail given). | Whole of population births from Alaska (N = 33,923) & California (N = 1,538,994). Maternal ethnicity for Alaska vs. California: 25.1% vs. 0.4% American Indian/Alaskan Native, 8.6% vs. 12.5% Asian/Pacific Islander, 3.8% vs. 5.4% Black, 6.1% vs. 50.6% Hispanic, 55.4% vs. 27.3% White, 1.0% vs. 3.9% other. 26.0% (95% CI 25.5%, 26.5%) of Alaskan births and 19.0% (95% CI= 8.9%, 19.1%) of Californian births experienced a report of harm to CPS before age 7 years. | Maltreatment reports (including physical abuse, sexual abuse, neglect, mental injury) [Outcome] | Alaska unadj. cumulative incidence = 26.0% (95% CI 25.5%, 26.5%); California unadj. cumulative incidence = 19.0% (95% CI 18.9%, 19.1%). Cumulative incidence similar for each race across states; race-adj. estimate shows relative risk for maltreatment in Alaska vs. California as 1.10, i.e. 10% higher risk of CPS report before age 7 in Alaska. |
| Putnam-Hornstein (2013a)<br><br>USA | **Purpose:** Descriptive<br><br>**Aims:** To document the abuse and neglect histories of adolescent mothers using official child protection records<br><br>**Outcomes:** Allegations and substantiations of maltreatment, foster care placements | 1. **Health** (vital birth records); **social services** (CPS records)<br>2. No<br>3. Yes<br>4. Yes<br>5. 0-19 years | Retrospective, one-time linkage using probabilistic linkage technique. Linked on personally identifiable data. Linkage validation/quality assessment ND. | N = 35,098 girls 12-19 years of age who gave birth in 2009. 70.2% Latina, 15.7% White, 11.0% Black, 2.3% Asian/Pacific Islander, 0.8% Native American. 44.9% had allegations of maltreatment (35.2% neglect, 19.5% physical abuse, 17.3% sexual abuse, 13.1% emotional abuse), 20.8% had substantiated maltreatment reports (17.1% neglect, 10.4% physical abuse, 9.4% sexual abuse, 7.8% emotional abuse), and 9.7% had been placed in foster care. | Allegations and substantiations of maltreatment (incl. sexual abuse/exploitation, physical abuse, neglect, emotional abuse) [Outcome] | 44.9% of the sample had allegations of maltreatment (35.2% neglect, 19.5% physical abuse, 17.3% sexual abuse, 13.1% emotional abuse), 20.8% had substantiated maltreatment reports (17.1% neglect, 10.4% physical abuse, 9.4% sexual abuse, 7.8% emotional abuse), and 9.7% had been placed in foster care. Maltreatment histories differed by age at birth, first vs. repeat birth, race/ethnicity, smoking during pregnancy, and birth payment method (public vs. private insurance) (all $p < 0.001$). |

| | | | | | | |
|---|---|---|---|---|---|---|
| Putnam-Hornstein (2017)<br><br>USA | **Purpose:** Descriptive<br><br>**Aims:** To develop a population-level, epidemiological characterisation of the child protection histories of young adults accessing homelessness services<br><br>**Outcomes:** CPS involvement | 1. **Social services** (Homeless Management Information System Records, CPS records)<br>2. No<br>3. Yes<br>4. No<br>5. 0-24 years (homelessness measured 17-24, CPS involvement from birth) | Retrospective, one-time linkage using probabilistic linkage technique. Linked on a combination of unique (social security number) and non-unique (first name, middle name, last name, date of birth, gender) identifiers. Linkage validation/quality assessment ND. | N = 2241 young people aged 17-24 with an encounter with homelessness services system 2011-2014. 51.1% female; 41.3% Black, 22.0% Hispanic, 18.7% White, 18.0% other/missing ethnicity. 50.0% had ≥1 maltreatment report (16.6% sexual abuse, 29.5% physical abuse, 35.8% neglect, 14.9% emotional abuse). 16.5% had a substantiated report but no foster care placement and 41.3% had a substantiated report with foster care placement. | Allegations and substantiations (+/- foster care) of maltreatment (sexual abuse, physical abuse, emotional abuse, neglect) [Outcome] | 50.0% of youth who had been in contact with homelessness services had ≥1 maltreatment report (16.6% sexual abuse, 29.5% physical abuse, 35.8% neglect, 14.9% emotional abuse (N.B. could have more than one type of report)). Of those with reports, 16.5% had a substantiated report but no foster care placement and 41.3% had a substantiated report with foster care placement. Females were more likely than males to have any report (58.1% vs. 41.5%) and were more likely to have an allegation of each type of maltreatment. Black youth had greater rates of CPS reports than White youth (59.8% vs. 31.8%) and were more likely than White youth to have reports of sexual abuse, physical abuse, and neglect (but not emotional abuse). History of CPS involvement was lower for youth accessing emergency shelter services and greater for those accessing homelessness prevention/rapid re-housing services as well as transitional and permanent supportive housing. |
| Rouland (2018)<br><br>New Zealand | **Purpose:** Descriptive<br><br>**Aims:** To document the cumulative prevalence among New Zealand children of CPS notifications, substantiated maltreatment cases, and out-of-home placements<br><br>**Outcomes:** CPS notifications, substantiated maltreatment | 1. New Zealand's Integrated Data Infrastructure: **health** (birth registration records); **social services** (Ministry of Social Development's Children, Youth and Family (CPS) register); **other** (New Zealand Customs Service (international travel and migration data))<br>2. No<br>3. Yes | Retrospective, one-time linkage. Linkage technique (probabilistic vs. deterministic) ND. Linked on unique identifier. Data were de-identified. Linkage validation/quality assessment ND. | N = 55,443 children born/adopted in New Zealand in 1998. 48.6% female. 23.5% had a CPS report of concern; 9.7% had substantiated reports (5.3% emotional abuse, 3.8% neglect, 3.1% physical abuse, 2.0% sexual abuse). | CPS reports and substantiations (emotional abuse, neglect, physical abuse, sexual abuse) [Outcome] | Cumulative prevalence of CPS reports by age 17 was 23.5% (24.1% for girls, 23.0% for boys); cumulative prevalence of substantiated reports was 9.7% (10.6% for girls, 8.9% for boys). Prevalence of maltreatment types were 5.3% for emotional abuse, 3.8% for neglect, 3.1% for physical abuse, and 2.0% for sexual abuse. Median ages for substantiations for each type of abuse were 6 years for neglect, 9 years for emotional abuse, 11 years for physical abuse, and 13 years for sexual abuse. |

| | | | | | | |
|---|---|---|---|---|---|---|
| | notifications, out-of-home placements | 4. No<br>5. 0-17 years | | | | |
| Segal (2019)<br><br>Australia | **Purpose:** Descriptive<br><br>**Aims:** To describe lifetime involvement in CPS, by type of contact<br><br>**Outcomes:** CPS involvement before age 18 | 1. **Education** (South Australia Schools Census); **health** (South Australia Birth Registry, Perinatal Statistics Collection, hospital in-patient and Emergency Department attendance, South Australia Death Registry); **social services** (South Australia Department for Child Protection records)<br>2. No<br>3. Yes<br>4. No<br>5. 0-18 years | Retrospective, one-time linkage using probabilistic linkage techniques (from a larger research database). Data were de-identified. Quality of matching 'continually checked' (no further detail provided). | All live births in South Australia 1986-2017 (N = 608,547). 4.2% Aboriginal. 3.2-3.6% of non-Aboriginal and 19.3-25.2% of Aboriginal children experienced substantiated maltreatment by age 18. | Child protection notifications, divided into 3 categories: 1) Notifier Only Concern (low risk of serious harm or inadequate information), 2) Child Protection Matters familial (high suspicion of a child at risk of serious maltreatment ('screened-in')), 3) all other notification types ('screened out'); child protection investigations; child protection substantiations; out-of-home care placements [Outcome] | Prevalence of child protection involvement varied across birth cohorts. Prevalence of Child Protection Matters notifications before age 18 were 9.5-14.7% for non-Aboriginal children and 39.9-55.7% for Aboriginal children; prevalence of investigations were 5.0-7.8% for non-Aboriginal children and 34.1-39.4% for Aboriginal children; prevalence of substantiations was 3.2-3.7% for non-Aboriginal children and 19.3-25.2% for Aboriginal children. 0.74-0.93% of non-Aboriginal children and 6.5-9.3% of Aboriginal children experienced entrance into out-of-home care following substantiation. Most CPS involvement types increased until 2010 and occurred earlier in life. There were strong associations between socioeconomic disadvantage and all child protection outcomes for both Aboriginal and non-Aboriginal children, with higher risk for the most disadvantaged non-Aboriginal children compared with least disadvantaged non-Aboriginal children. |

| | | | | | | |
|---|---|---|---|---|---|---|
| Ubbesen (2015)<br><br>Denmark/ England (linkage in Denmark only) | **Purpose:** Descriptive<br><br>**Aims:** To provide a descriptive analysis of the extent to which age at first entry into out-of-home care varies between England and Denmark and how this varies over time<br><br>**Outcomes:** First entry into out-of-home care | (NB information for Denmark only)<br><br>1. **Social services** (Register of Support for Children and Adolescents); **other** (population register)<br>2. No<br>3. Yes<br>4. No<br>5. 0-16 years | Retrospective, one-time linkage using deterministic linkage technique. Linked on personal identification number. Linkage validation/quality assessment ND. | All children in Denmark born 1992-2008, of whom N = 15,644 were placed in out-of-home care. 45.7% female; 89% long-term citizens. (Denmark only) | Out-of-home care placement ('in most cases' due to concerns of child maltreatment) [Outcome] | In England, foster care was the predominant first out-of-home care placement (78%) vs. residential care in Denmark (54%). Cumulative incidence for first entry into out-of-home care for children under 3 years was similar countries. In England, the age-specific cumulative incidence increased with each successive birth cohort at all ages at first entry into out-of-home care. For infants, the rate of entry into care increased from 2.89 per 1000 for children born in 1992–1994 to a peak of 5.88 per 1000 for children born in 2004–2006. Similar absolute increases were seen for first entry by 10 years of age (11.68 per 1000 – if born in 1992–1994 to 13.74 per 1000 if born in 1998–2000). |
| **Risk factors** | | | | | | |
| Austin (2018)<br><br>USA | **Purpose:** Descriptive<br><br>**Aims:** To examine preconception and prenatal predictors of time to first CPS contact among Alaskan children<br><br>**Outcomes:** CPS contact | 1. Alaska Longitudinal Child Abuse and Neglect Linkage (ALCANLink): **health** (Alaska Pregnancy Risk Assessment Monitoring System (PRAMS; population survey of mothers and infants), Alaska Birth Defects Registry, Alaska Child Death Review, death certificates); **social services** (Alaska Office of Children's Services (CPS) records, Alaska Department of Revenue records); **other aggregate data** (geographic census | Retrospective, one-time linkages using deterministic and probabilistic (iterative) linkage techniques. Linked on first, last, and alias names, date of birth and sex. Responding to PRAMS survey indicated consent for linkage. Data were shared with researchers in de-identified format. Match rates showed 94.1% of all PRAMS births could be linked to records from the Department of Revenue records (the most universal of the datasets). See Parrish | N = 3549 children; maternal ethnicity was 35.4% Alaska Native/American Indian, 59.2% non-Native. 30.9% had a CPS contact in the follow-up time. | CPS contact was defined as any allegation of physical or sexual abuse, neglect, or mental injury (i.e., emotional abuse) (regardless of investigation/subst antiation) [Outcome] | Significant predictors of time to first CPS contact included low socioeconomic status (HR=2.23, 95% CI 1.68, 2.96), maternal smoking during pregnancy (HR = 1.87, 95% CI 1.55, 2.24), unmarried maternal marital status (HR = 1.62, 95% CI 1.31, 1.99), urban residence (HR = 1.59, 95% CI 1.32, 1.92), lower maternal education (HR = 1.54, 95% CI 1.24, 1.92), maternal Alaska Native/American Indian race (HR = 1.40, 95% CI1.15, 1.71), maternal experience of intimate partner violence in the 12 months before childbirth (HR = 1.32, 95% CI 1.01, 1.74), a greater number of living children (HR = 1.20, 95% CI 1.13, 1.29), a greater number of stressful life events in the 12 months before childbirth (HR = 1.16, 95% CI 1.11, 1.21), and younger maternal age at childbirth (HR = 0.95, 95% CI 0.93, 0.97). |

| | | | | | | |
|---|---|---|---|---|---|---|
| | | classification data)<br>2. Yes<br>3. Yes<br>4. Yes<br>5. 0-6 years | (2017) for additional detail on quality assessment and match rates. | | | |

| | | | | | | |
|---|---|---|---|---|---|---|
| Cant (2019)<br><br>Australia | **Purpose:** Descriptive<br><br>**Aims:** To investigate whether children living in areas with high levels of household overcrowding are at higher risk for child sexual abuse allegations and substantiations<br><br>**Outcomes:** Sexual abuse allegations and substantiations | 1. Western Australia Data Linkage System: **health** (Midwives Notification System, Hospital Morbidity Data Collection records, Mental Health Information System, Birth Registrations, Mortality Register); **social services** (Department for Child Protection and Family Support (CPS) records); **other (aggregate) data** (Index of Education and Occupation, Australian Bureau of Statistics Census of Population and Housing)<br>2. No<br>3. Yes<br>4. Yes<br>5. 0-18 years | Retrospective, one-time linkage. Linkage technique (probabilistic vs. deterministic) ND. Linked on name, address, and birthdate. Implied exemption from consent requirements. Data were shared with researchers in a de-identified format. Reported linkage quality of 97-98%. | N = 524,478 children in Western Australia aged 0–18 years (born 1990-2010). 48.8% female; 6.0% Aboriginal, 93.9% non-Aboriginal. 2.1% had a sexual abuse allegation and 0.5% had a substantiated sexual abuse allegation. | Allegations and substantiations of sexual abuse [Outcome] | Greater household overcrowding was associated with a 23%-46% increase in the risk of child sexual abuse allegations (following a dose-response relationship). The highest level of overcrowding was associated with a 40% increased risk of substantiated sexual abuse (adj. HR = 1.40, 95% CI 1.16, 1.68). Other factors significantly associated with an increased risk of allegation or substantiated report of sexual abuse (in adjusted models) included female gender, Aboriginality, younger maternal age, higher socio-economic disadvantage, maternal mental health contact, maternal substance abuse, marital status, and later year of birth; paternal age and remoteness area did not show a consistent relationship. |

| | | | | | | |
|---|---|---|---|---|---|---|
| Eastman (2016)<br><br>USA | **Purpose:**<br>Descriptive<br><br>**Aims:** To identify classes of children who had been reported to CPS as infants and may be at increased risk of a re-report during the first 5 years of life<br><br>**Outcomes:** Re-report of child maltreatment to CPS | 1. **Health** (vital birth records); **social services** (CPS records)<br>2. No<br>3. Yes<br>4. No (maternal characteristics taken from offspring birth records)<br>5. 0-5 years | Retrospective, one-time linkage using probabilistic linkage technique. Linked on a combination of unique and non-unique parent and child identifiers. Data de-identified after linkage. Quality assessment showed a match rate of 91.9%; manual cleaning eliminated an additional 125 records with inconsistencies. | N = 23,871 infants reported to CPS during the first year of life (and remaining at home following report). 48.6% female; 30.1% White, 29.5% US-born Hispanic, 20.5% foreign-born Hispanic, 14.0% Black, 3.3% Asian/Pacific Islander, 1.5% Native American. 74.2% had a first report of neglect/substantial risk, 10.4% of emotional abuse, 8.9% of sibling risk, and 6.6% of abuse (physical or sexual). | Initial CPS report in first year of life (abuse, neglect or substantial risk, emotional abuse, sibling risk); all outcomes included (evaluated out, unfounded, inconclusive, substantiated without services, substantiated with services) [Exposure]<br><br>CPS re-report: any CPS report following the initial report up until 5 years of age, regardless of investigation/ substantiation of first report but excluding when the first report was still under investigation (N = 362) [Outcome] | 60.7% of children were re-reported within 5 years. Re-reported children were more likely than those who were not re-reported to have a health condition at birth (18.2% vs. 15.6%), no established paternity (30.4% vs. 20.8%), a family history of CPS involvement with older siblings (50.7% vs. 35.1%), a young mother (<19 years old; 31.7% vs. 17.9%), a mother with low education (high school degree or less; 73.5% vs. 66.5%), a mother who received late or no prenatal care, or a mother who had public health insurance (76.5% vs. 66.5%). Asian/Pacific Islander and foreign-born Hispanic maternal ethnicity was underrepresented in re-reported children. Re-reported children were also more likely to have an initial report of neglect (76.6% vs. 70.4%), a substantiated initial report with services (11.3% vs. 7.8%), and to have been reported by a mandated reporter (14.6% vs. 10%). Latent class analysis identified four classes of children differentiated by risk of re-report: class 1 (‘lowest risk (less education)’; 19.0% of infants), class 2 (‘lowest risk (more education)’; 21.0% of infants), class 3 (‘medium risk (young mothers)’; 27.0% of infants), and class 4 (‘highest risk (CPS history)’; 33.0% of infants). Classes 1 and 2 had the lowest probability of re-report (44%), then class 3 (60%), then class 4 (78%). |

| | | | | | | |
|---|---|---|---|---|---|---|
| Ekéus (2004)<br><br>Sweden | **Purpose:** Descriptive<br><br>**Aims:** To explore whether children of teenage mothers have an increased risk of hospital admissions or death due to unintentional and violent injuries compared with children of older mothers. To investigate whether or not the risks were mediated by social factors or by parental psychiatric morbidity and/or substance misuse<br><br>**Outcomes:** Hospital admissions or death due to unintentional and violent injuries | 1. **Education** (Swedish Register of Education); **health** (Swedish Medical Birth Register, National Cause of Death Register, Swedish Hospital Discharge Register); **social services** (Swedish Register of Children and Young Persons Subjected to Child Welfare Measures); **other** (Register of the Total Population, Sweden's Total Enumeration Income Survey, Swedish Population and Housing Census)<br>2. No<br>3. Yes<br>4. Yes<br>5. 0-7 years | Retrospective, one-time linkage using deterministic linkage technique (within a system using national registries). Linked on unique personal identification numbers of child and mother. Implied exemption from active consent. 99% linkage between child and mother, 99.4% linkage between child and father, and 91.0% linkage with maternal grandmother. | N = 800,190 children born in Sweden between 1987 and 1993. 48.6% female. N = 3729 with mother aged 12-17 and N = 18,097 with mother aged 18-19. | ICD-9 or -10 code for child abuse [Outcome] | N = 93 children had a hospital admission with an ICD code for abuse, with higher rates amongst children of younger mothers. Crude rates per maternal age were 1.1 per 1000 for ages 12-17, 0.6 per 1000 for ages 18-19, 0.2 per 1000 for ages 20-24, and 0.1 for ages 25-28, 29-32, and 33-55. |
| Finno-Velasquez (2017)<br><br>USA | **Purpose:** Descriptive<br><br>**Aims:** To examine subgroup patterns of CPS involvement in California for Asian and Pacific Islander children prospectively based on maternal nativity and ethnic origin<br><br>**Outcomes:** CPS involvement (maltreatment reports before age 5, regardless of | 1. **Health** (vital birth records); **social services** (CPS records)<br>2. No<br>3. Yes<br>4. No (maternal characteristics collected from offspring birth record)<br>5. 0-5 years | Retrospective, one-time linkage using probabilistic linkage technique. Linked on a combination of unique (maternal social security number) and non-unique (first name and last name, date of birth) child and parent identifiers. Linkage validation/quality assessment ND. | All California births in 2006-2007 for which maternal race and ethnicity was self-reported as Asian in the birth record (N = 138,858; 12.2% of the entire 2006-2007 birth cohort). 4.7% of children of foreign-born mothers had a CPS report vs. 9.5% of children of native-born mothers had a CPS report. | Any maltreatment report before age 5 (regardless of investigation or substantiation) [Outcome] | 9.5% children of US-born mothers had a CPS report before age 5, compared with 4.7% of children with foreign-born mothers. After adjusting for other known risk factors, the highest rates of CPS reports were in children of US-born Hawaiian/Guamanian/Samoan and Pacific Islander mothers (20.4% and 18.0%, respectively; adj. RRs = 2.12, 95% CI 1.93, 2.32 and 1.93, 95% CI 1.71, 2.18, respectively, compared with all other children born to Asian mothers). For other groups (e.g. foreign-born Pacific Islander and Hmong mothers), relatively high crude risk ratios were greatly attenuated in the adjusted model. |

| | | | | | | |
|---|---|---|---|---|---|---|
| | substantiation) | | | | | |
| Hafekost (2017a)<br><br>Australia | **Purpose:** Descriptive<br><br>**Aims:** To examine the relationship between a maternal alcohol-use diagnosis, and the timing of diagnosis, and child protection outcomes in a Western Australian population cohort<br><br>**Outcomes:** Substantiated child maltreatment allegations and out-of-home care placement | 1. Western Australian Data Linkage System: **health** (Midwives Notification System, Hospital Morbidity Data System, Mental Health Information System, Drug and Alcohol Office, Intellectual Disability Database, Western Australian Register of Developmental Anomalies); **social services** (Department for Child Protection and Family Support (CPS) records)<br>2. No<br>3. Yes<br>4. Yes<br>5. 0-ND | Retrospective, one-time linkage using probabilistic linkage technique (from a larger 'living' research database using multiple/updated linkages). Linked on medical record number, first and last name, initial, date of birth, sex, and addresses. Data were de-identified. Linkage quality assessed through clerical review of a sub-sample of matches; very low (~0.1%) false positive and false negative matches. | N = 84,245 (N = 23,509 of whom had an alcohol-related diagnosis & N = 60,736 controls). 48.6% of children with maternal alcohol-related diagnoses were female vs. 48.9% of controls. 41.3% of mothers with an alcohol-related diagnosis were Indigenous. 17.6% of children whose mothers had an alcohol-related diagnosis had at least one substantiated maltreatment report (of these, 53% had 1+ report of neglect, 21.0% physical abuse, 14.6% sexual abuse, 11.3% emotional and psychological abuse), vs. 4.0% for controls (of these, 36.6% had 1+ report of neglect, 28.5% physical abuse, 24.2% sexual abuse, 10.7% emotional and psychological abuse); 13.4% and 2.1% had been placed into out-of-home care, respectively. | Contact with CPS included substantiated child maltreatment reports (neglect, physical abuse, sexual abuse, emotional and psychological abuse) and out-of-home care placements (due to substantiated claims or because a caregiver can no longer look after them, e.g. has died) [Outcome] | Children with maternal alcohol-related diagnoses had a significantly greater risk of a substantiated maltreatment report (adj. OR = 2.92, 95% CI 2.71, 3.14) and out-of-home care placement (adj. OR = 3.78, 95% CI 3.46, 4.13). Children at greatest risk of substantiated maltreatment were those with mothers who received their diagnosis during pregnancy (adj. OR = 4.85, 95% CI 4.25, 5.53), children with Indigenous mothers (adj. OR = 2.11, 95% CI 1.96, 2.28), low socioeconomic status (adj. OR for bottom 10% vs. top 10% = 2.99, 95% CI 2.25, 3.95), children of young mothers (adj. OR for <25 years vs. 25-30 years = 1.40, 95% CI 1.31, 1.50), mothers not married (adj. ORs = 1.38, 95% CI 1.31, 1.47 for never married; 1.55 (1.45, 1.79) for separated/widowed/divorced), maternal mental health record (adj. OR = 1.90, 95% CI 1.77, 2.05), maternal illicit drug record (adj. OR = 1.98, 95% CI 1.82, 2.16), child Fetal Alcohol Spectrum Disorder diagnosis (adj. OR = 3.25, 95% CI 2.28, 4.63), child intellectual disability (adj. OR = 1.86, 95% CI 1.65, 2.10), later parity (adj. OR for 3+ vs. only child = 1.88, 95% CI 1.71, 2.06), and low birthweight (adj. OR for <10th percentile vs. normal = 1.21, 95% CI 1.13, 1.29). Similar patterns were found for out-of-home care placements. |

| | | | | | | |
|---|---|---|---|---|---|---|
| Hafekost (2017b)<br><br>Australia | **Purpose:** Descriptive<br><br>**Aims:** To investigate whether a child whose mother has an alcohol use disorder diagnosis has an increased risk of contact with the justice system in comparison with other children and if so, whether the relationship persists when known sociodemographic and child risk factors are controlled for<br><br>**Outcomes:** Justice system contact | 1. Western Australian Data Linkage System: **education** (Western Australian Literacy and Numeracy Assessment and National Assessment Program—Literacy and Numeracy); **health** (Midwives Notification System, Hospital Morbidity Data System, Mental Health Information System, Drug and Alcohol Office, Intellectual Disability Database, Western Australian Register of Developmental Anomalies); **justice** (Department of Corrective Services dataset); **social services** (Department for Child Protection and Family Support (CPS) records)<br>2. No<br>3. Yes<br>4. Yes<br>5. 0-ND | Retrospective, one-time linkage using probabilistic linkage technique (from a larger 'living' research database using multiple/updated linkages). Linked on medical record number, first and last name, initial, date of birth, sex, and addresses. Data were de-identified. Linkage quality assessed through clerical review of a sub-sample of matches; very low (~0.1%) false positive and false negative matches. | N = 67,283 children born in Western Australia (N = 18,740 whose mothers had an alcohol use diagnosis and N = 48,543 controls). 48.8% female; 35.8% Indigenous. | CPS contact: substantiated allegations of maltreatment and out-of-home care placements [Exposure (covariate)] | Children with a child protection contact were more likely than those without to have contact with the justice system (adj. OR = 2.27, 95% CI 2.09, 2.47). |

| | | | | | | |
|---|---|---|---|---|---|---|
| Högberg (2019)<br><br>Sweden | **Purpose:** Descriptive<br><br>**Aims:** To analyse infants placed in out-of-home care in Sweden by incidence, medical diagnoses, and perinatal factors.<br><br>**Outcomes:** Out-of-home care by category (problems relating to social environment/up-bringing; abuse diagnoses without SDH (subdural haemorrhage), RH (retinal haemorrhage), rib fracture, or long bone fracture; and SDH, RH, rib fracture, or long bone fracture) before 1 year of age | 1. **Education** (Education Register); **health** (Swedish Medical Birth Register, National Patient Register (inpatient and outpatient)); **social services** (Register of Children and Young Persons Subjected to Child Welfare Measures (CPS))<br>2. No<br>3. Yes<br>4. Yes<br>5. 0-1 year | Retrospective, one-time linkage using a deterministic linkage technique (within a system using national registries). Linked on personal identity number. Exempt from consent requirements. Linkage validation/quality assessment ND. | N = 1,855,267 live births in Sweden between 1997 and 2014, of which N = 395,812 had any entry in the National Patient Register before age 1, N = 182,974 had one of 119 select diagnoses of interest, N = 1514 had child welfare involvement, and N = 782 (51.6%) had a diagnosis related to abuse or adverse social or parental circumstances before first entry in the register | Out-of-home care placement due to social environment/up-bringing; abuse diagnoses without SHD/RH, or rib/long bone fractures; and SHD/RH, or rib/long bone fractures (high predictive value for abuse) [Outcome] | 23.3% of infants with an entry in the National Patient Register had a diagnosis of SDH, RH, or rib/long bone fracture and 9% had a diagnosis of abuse without any of these. Compared with the general population, infants with a diagnosis of SDH, RH, or rib/long bone fractures were significantly more likely to be male, multiple born, preterm, or small for gestational age, and to have mothers who were primipara or 4+ multipara, young, over- or underweight, smokers, single, not Nordic-born, and less educated. Compared with infants in out-of-home care without these diagnoses, they were more likely to be male and less likely to have a mother who smoked or was single.<br><br>(N.B. study also included incidence estimates, but these were derived through a single database rather than using the linked data and so are not included in this review) |

| | | | | | | |
|---|---|---|---|---|---|---|
| Johnson-Motoyama (2015)<br><br>USA | **Purpose:** Descriptive<br><br>**Aims:** To determine whether maternal foreign-born status conferred a protective advantage against reported and substantiated maltreatment across Hispanic-origin groups, and whether the likelihood an infant was reported or substantiated for maltreatment varied by Hispanic origin<br><br>**Outcomes:** CPS involvement in first year of life | 1. **Health** (vital birth records); **social services** (CPS records)<br>2. No<br>3. Yes<br>4. No (maternal characteristics taken from offspring birth records)<br>5. 0-1 year | Retrospective, one-time linkage using probabilistic linkage technique. Linked on a combination of unique (maternal social security number) and non-unique (first name and last name, date of birth) child and parent identifiers. Identifiable data. Linkage validation/quality assessment ND. | All California births occurring 2000-2006 where maternal race/ethnicity was coded as Hispanic origin (N = 1,909,155). 87.4% Mexican heritage, 9.7% Central or South American, 1.9% 'other Hispanic', 0.8% Puerto Rican, 0.3% Cuban. 63.4% born to foreign-born mothers. | Alleged abuse/neglect during the first year of life (reported vs. not reported) and substantiated report of abuse/neglect (substantiated vs. unsubstantiated) [Outcome] | 4.4% of children born to Hispanic mothers were reported to CPS during the first year of life. 7.2% of children of US-born mothers and 2.7% of children born to foreign-born mothers had maltreatment reports, with children of US-born mothers at higher risk than foreign-born mothers for each origin group. The highest rates of maltreatment reports were for US-born Puerto Rican, Mexican, and Cuban mothers (10.0%, 7.2%, and 6.5%, respectively) and the lowest were for foreign-born Mexican, Central/S. American, and Cuban mothers (2.7%, 2.9%, and 3.0%, respectively). For foreign-born mothers, all groups were more likely to have maltreatment reports than children born to Mexican mothers (adj. RRs 1.22-2.80, $p < 0.001$). For foreign-born mothers, children born to Puerto Rican and Cuban mothers were more likely to have maltreatment reports than children born to Mexican mothers (adj. RRs 1.43-1.59, $p < 0.001$). 1.5% of children born to Hispanic mothers had a substantiated report of maltreatment in the first year of life. The highest rates of substantiated maltreatment reports were for US-born Puerto Rican, Mexican, and Cuban mothers (2.7%, 2.8%, and 2.8%, respectively) and the lowest were for foreign-born Mexican, Central/S. American, and Cuban mothers (0.8%, 0.9%, and 1.1%, respectively). Patterns in the adj. RRs were similar to those for reported maltreatment. |

| | | | | | | |
|---|---|---|---|---|---|---|
| Kalland (2006)<br><br>Finland | **Purpose:** Descriptive<br><br>**Aims:** To investigate retrospectively neonatal health and maternal background among a sample of children taken into custody and placed in foster care. To investigate the relation between medical and social risk in the neonatal period<br><br>**Outcomes:** Foster care placement | 1. **Health** (Finnish Medical Birth Registry); **social services** (Finnish Child Welfare Registry (CPS) records)<br>2. No<br>3. Yes<br>4. No (maternal characteristics collected from offspring birth records)<br>5. 0-18 years | Retrospective, one-time linkage using deterministic linkage technique (within a system using national registries). Linked on personal identification numbers. Anonymised data. Linkage validation/quality assessment ND. | N = 1668 children born in 1987-89 placed in foster care due to maltreatment and all Finnish births in 1987 (N = 59,727) as controls. For foster care children: 48.6% female; mean age at care placement = 53 months. | Foster care placement specifically due to maltreatment (abuse or neglect) [Outcome] | Bivariate analyses indicated that children later placed in foster care had lower birth-weight and -length, shorter gestational age, lower 1-minute Apgar scores, and later nursery discharge compared with controls. They further had a greater proportion of teenage, first-time, and unmarried mothers, as well as mothers who smoked (all $p<0.001$). Poorer health at birth was not fully explained by maternal smoking during pregnancy. |

| | | | | | | |
|---|---|---|---|---|---|---|
| King (2013)<br><br>USA | **Purpose:** Descriptive<br><br>**Aims:** To examine whether the status and identity of the maltreatment reporter are independent predictors of substantiation across maltreatment types and in the presence of multiple sociodemographic characteristics and risk factors present at birth<br><br>**Outcomes:** Substantiation of alleged maltreatment | 1. **Health** (birth records); **social services** (California Child Welfare Services/Case Management System (CPS) records)<br>2. No<br>3. Yes<br>4. No (maternal characteristics collected from offspring birth record)<br>5. 0-5 years | Retrospective, one-time linkage using probabilistic linkage technique. Linked on a combination of unique (e.g. social security number) and non-unique (e.g. first name, date of birth) child and parent identifiers. Linkage quality assessed by manual review; match rate of CPS records to birth records was 84% (not matched included those with missing information and those born out of state). | N = 59,413 children born in 2002 in California reported and investigated for maltreatment before age 5. Maternal ethnicity: 52.9% Latina, 28.9% White, 12.6% Black, 4.5% Asian/Pacific Islander, 1.0% Native American. 73.9% of children had unsubstantiated maltreatment reports; 26.1% had substantiated reports. Allegation types were sexual abuse (2.8%), physical abuse (8.9%), neglect (49.8%), emotional abuse (13.2%), and substantial risk (25.2%). | Five types of reported and investigated maltreatment (listed hierarchically): (1) sexual abuse (sexual abuse or exploitation); (2) physical abuse; (3) neglect (severe neglect, general neglect, or caretaker absence or incapacity); (4) emotional abuse (may include witnessing domestic violence); and (5) substantial risk (at risk due to sibling being maltreated or substantial risk of abuse).<br>[Substantiation = outcome; type, reporter identity = predictors] | 26.1% of children had substantiated maltreatment reports before age 5. Reports from mandated reporters were more likely to be substantiated than those from non-mandated reporters (adj. RR = 2.49, 95% CI 2.40, 2.60). Reports from law enforcement/legal services, medical professionals, public agencies, and other professionals were more likely to be substantiated than reports from non-mandated reporters while reports from school/childcare personnel and helping professionals were more likely to be substantiated than those from family, community members, and unidentified reporters. Type of reported maltreatment was also a significant predictor of substantiation: compared with physical abuse, sexual abuse was less likely to be substantiated (adj. RR = 0.84, 95% CI 0.71, 0.98) while substantial risk, emotional abuse, and neglect were all more likely to be substantiated (adj. RRs = 1.62, 1.80, and 2.36, respectively). |

| | | | | | | |
|---|---|---|---|---|---|---|
| Maloney (2017)<br><br>USA | **Purpose:** Descriptive<br><br>**Aims:** To examine the relative contributions of different risks to Black-White disparities in maltreatment reports and foster care placements<br><br>**Outcomes:** CPS contact | 1. **Health** (vital/birth registration records, Allegheny County behavioural health and drug and alcohol systems records); **justice** (Allegheny County prison and youth justice systems); **social services** (Allegheny County child protection (CPS) data systems)<br>2. No<br>3. Yes<br>4. Yes<br>5. 0-4 years | Retrospective, one-time linkage. Linkage technique (probabilistic vs. deterministic) ND (linked on a combination of unique and non-unique child and parental identifiers, e.g. first name, last name, maternal Social Security Number, date of birth). Anonymised data. Linkage validation/quality assessment ND. | All live births to Black and White families in one county in Pennsylvania, USA 2008-2010 (N = 27,527; 21.1% Black families). 20.0% and 6.9% children from Black and White families, respectively, had a CPS report by age 4; 4.0% and 1.3% had placements by age 4, respectively. | Allegations of maltreatment (abuse and neglect) and foster care placement before age 4 [Outcome] | In unadjusted models, children of Black families had 2.9 times the risk of a CPS report by age 4 and 3.1 times the risk of foster care placement. However, these differences were completely explained by between-group differences in parental marital status (single motherhood) and age at birth (early parenthood). |
| McDonnell (2019)<br><br>USA | **Purpose:** Descriptive<br><br>**Aims**: To evaluate the odds of experiencing maltreatment among children with autism spectrum disorder (ASD) and/or intellectual disability (ID) in comparison to population controls<br><br>**Outcomes:** Maltreatment reports and substantiations | 1. **Health** (South Carolina Autism and Developmental Disabilities Monitoring Network, birth records); **social services** (South Carolina Department of Social Services (CPS) records)<br>2. No<br>3. Yes<br>4. No<br>5. 2-18+ years | Retrospective, one-time linkage. Linkage technique (probabilistic vs. deterministic) ND. Linkage validation/quality assessment ND. | N = 4,988 children born in 1992, 1994, 1996, and 1998 (N = 316 ASD-only, N = 291 ASD + ID, N = 1280 ID-only, N = 3101 population controls). Proportions with a maltreatment report/ maltreatment substantiation for each group were 21.2%/10.1% for ASD-only, 31.3%/16.5% for ASD + ID, 39.2%/24.7% for ID-only, and 16.1%/8.8% for ID-only. | Reports and substantiations of maltreatment (sexual abuse, physical abuse, emotional abuse, physical neglect) [Outcome] | All three study groups were more likely than population controls to have any maltreatment report (adj. ORs = 1.86, 95% CI 1.36, 2.52 for ASD-only; 2.35, 95% CI 1.77, 3.12 for ASD + ID; and 2.45, 95% CI 2.09, 2.88 for ID-only). The same was true for substantiations (adj. ORs = 1.51, 95% CI 1.01, 2.26 for ASD-only; 1.97, 95% CI 1.39, 2.79 for ASD + ID; and 2.49, 95% CI 2.05, 3.02 for ID-only). Individuals in the ASD-only group were significantly more likely than controls to have reports of physical abuse and physical neglect and substantiations of physical neglect. Individuals in the ASD + ID group were significantly more likely than controls to have reports all types of maltreatment and substantiations of physical neglect. Individuals in the ID-only group were significantly more likely than controls to have reports and substantiations of all types of maltreatment. |

| | | | | | | |
|---|---|---|---|---|---|---|
| O'Donnell (2015)<br><br>Australia | **Purpose:** Descriptive<br><br>**Aims:** To quantify the relationship between maternal mental health and risk of child maltreatment according to the different types of mental health diagnoses<br><br>**Outcomes:** Child maltreatment allegations and substantiations | 1. Western Australian Data Linkage System: **health** (Intellectual Disability Exploring Answers dataset, Cerebral Palsy Register, Birth Defects Register, Hospital Morbidity Data System, Mental Health Information System); **social services** (Department of Child Protection and Family Support (CPS) records)<br>2. No<br>3. Yes<br>4. Yes<br>5. 0-17 years | Near real-time ('living') linkage from multiple updates using probabilistic linkage technique. Linked on medical record number, first and last name, initial, date of birth, sex, and addresses. Data were de-identified. Linkage quality assessed through clerical review of a sub-sample of matches; very low (~0.1%) false positive and false negative matches. | All live births in Western Australia 1990-2005 (N = 404,022; N = 74,888 with mothers with a mental health contact). 48.7% female; 7.6% Aboriginal or Torres Strait Islander. 3.5% had a maltreatment allegation (primarily for neglect). | Child maltreatment allegations and substantiations [Outcome] | 9.2% of children whose mother had a prior mental health contact had a maltreatment allegation (approx. half of all children with allegations had mothers with a prior mental health contact). Prior maternal mental health contact was significantly associated with maltreatment allegations overall (adj. HR = 2.64, 2.50, 2.80) and across diagnostic categories (adj. HRs 1.54-13.15). Disorders most associated with allegations were intellectual disability (adj. HR = 13.15, 95% CI 9.40, 18.40), disorders of childhood/psychological development (adj. HR = 2.99, 95% CI 2.44, 3.67), personality disorders (adj. HR = 2.70, 95% CI 2.26, 3.23), substance-related disorders (adj. HR = 2.66, 95% CI 2.46, 2.89), and organic disorders (adj. HR = 2.48, 95% CI 1.53, 4.02). |
| Orr (2019)<br><br>Australia | **Purpose:** Descriptive<br><br>**Aims:** To examine the risk of maltreatment allegations in children whose mothers were hospitalised due to an assault<br><br>**Outcomes:** Maltreatment allegations | 1. **Health** (Midwives Notification System, birth registrations, Mental Health Information System, Western Australian Register of Developmental Anomalies, Intellectual Disability Exploring Answers Database, Hospital Morbidity Data Collection); **social services** (Department for Child Protection and Family Support (CPS) records)<br>2. No<br>3. Yes<br>4. Yes | Retrospective, one-time linkage (using data from a research database with near real-time linkage) using probabilistic linkage technique. Linked on medical record number, first and last name, initial, date of birth, sex, and addresses. Exempt from consent requirements. Data were de-identified. Clerical review showed linkage accuracy >99%. | All live births in Western Australia 1990-2009 and their parents (N = 524,534 children). 48.8% female; 7.8% Aboriginal or Torres Strait Islander. 6.6% had a maltreatment allegation (1.3% emotional abuse, 2.0% neglect, 1.4% physical abuse, 1.6% sexual abuse); 3.1% had a substantiated maltreatment allegation. | Allegation and substantiations of maltreatment (physical abuse, sexual abuse, emotional abuse, neglect); maternal assaults included domestic violence, but these cases were not separated from all assaults [Child maltreatment: outcome] | 20.3% of children whose mothers were hospitalised for assault had a subsequent maltreatment allegation; this rose to 41.9% for mothers hospitalised in the prenatal period. Maternal assault-related hospitalisation was a significant risk factor for subsequent maltreatment allegation (adj. HR = 9.20, 95% CI 8.98, 9.43, compared with children whose mothers had no assault-related admission). Aboriginal (adj. HR = 1.57, 95% CI 1.44, 1.71) and non-Aboriginal children (adj. HR = 1.93, 95% CI 1.79, 2.07) with maternal assault hospitalisations had a greater chance than those without hospitalisations of maltreatment allegation. |

| | | | | | | |
|---|---|---|---|---|---|---|
| | | 5. 0-approx. 23 years | | | | |

| | | | | | | |
|---|---|---|---|---|---|---|
| Papalia (2017)<br><br>Australia | **Purpose:** Descriptive<br><br>**Aims:** To investigate whether there are distinct temporal pathways of interpersonal re-victimisation between the ages of 10–25 years among medically confirmed child sexual abuse cases and whether abuse variables, re-victimisation variables, and presence of other adverse outcomes, were associated with heterogeneity in re-victimisation pathways<br><br>**Outcomes:** Interpersonal (re)-victimisation | 1. **Health** (Office of Forensic Medicine clinical records, Victoria's public psychiatric patient information system); **justice** (Victoria's criminal records database)<br>2. No<br>3. Yes<br>4. No<br>5. Sexual abuse occurred from 0-16 years; follow-up 13-44 years | Retrospective, one-time linkage using deterministic and probabilistic techniques. Linked on identifying information including surname, first name, date of birth or age range, gender. Consent not sought. Data were de-identified. Linkage 'checked for accuracy' (no further detail provided). | N = 510 participants with experience of interpersonal (re)-victimisation (N = 401 victims of sexual abuse and N = 109 comparisons). Sexual abuse victims: 77.1% female; mean age at follow-up = 24.38 years (SD 3.93). Comparison group: 67.0% female; mean age at follow-up = 23.32 years (SD 3.34). | Sexual abuse (could be by someone not related to the child); cases identified on the basis of findings from the Office of Forensic Medicine case files [Exposure] | Abuse victims were more likely than comparisons to have more interpersonal (re)-victimisation incidents, be older at the time of the most recent incident, be (re)-victimised for a longer time, be younger at time of first interpersonal (re)-victimisation. They were also 4.43 (95% CI 2.35, 8.35) times as likely to be the victim of a sexual offence, 3.30 (95% CI 2.07, 5.25) times as likely to have a personal safety intervention order, 2.12 (95% CI 1.32, 3.62) times as likely to have a criminal history, and 3.02 (95% CI 1.35, 6.80) times as likely to have been charged with a violent offence. Abuse victims were 3.02 (95% CI 1.75, 5.21) as likely as comparisons to have mental health system contact. LCA resulted in four pathways to interpersonal (re)-victimisation: (1) ‘Normative’ (relatively low rates of re-victimisation; 65% of female victims and 71.7% of males), (2) ‘Childhood-Limited’ (moderate rates of re-victimisation until age 14, followed by a sharp decline; 15.5% of female victims ad 18.5% of males), (3) ‘Emerging-Adulthood’ (low rates of re-victimisation during late childhood/adolescence followed by a spike in early adulthood; 10.4% of female victims and 5.3% of males), and (4) ‘Chronic re-victimisation’ (moderate-to-high rates of re-victimisation throughout early life course; 9.1% of female victims and 4.3% of males). Membership in more problematic and persistent trajectories was predicted by older age at abuse, criminal history, and mental health problems. |

| | | | | | | |
|---|---|---|---|---|---|---|
| Parrish (2011)<br><br>USA | **Purpose:** Descriptive<br><br>**Aims:** To assess the utility of combining Pregnancy Risk Assessment Monitoring System (PRAMS) data with CPS records to identify risk factors associated with Protective Services Reports suggestive of child maltreatment<br><br>**Outcomes:** CPS reports before age 4 | 1. **Health** (Pregnancy Risk Assessment Monitoring System, vital statistics); **social services** (Alaska Division of Family and Youth Services (CPS) records)<br>2. Yes<br>3. Yes<br>4. Yes<br>5. 0-4 years | Repeated one-time linkages. Linkage technique (probabilistic vs. deterministic) ND. Linked on using name, date of birth, birth certificate number, and Social Security Number. 90% of CPS records matched with a birth certificate. | N = 29,851 births in Alaska, USA 1997-1999; PRAMS sampled nearly 20% and after weighting represented N = 29,432 births. 13.9% of the full birth cohort and 13.5% of the PRAMS cohort had a CPS report in the study period. | Protective service reports (incl. physical abuse, emotional abuse, sexual abuse, and neglect) [Outcome]<br><br>Domestic violence/sexual assault (time of occurrence, e.g. before/during/after pregnancy ND) [Exposure] | 13.9% of all births and 13.5% of the PRAMS sample had a protection report before age 4. Risk factors included in the final multivariate model were low maternal age and education, domestic violence and sexual assault, maternal tobacco use, maternal marital status of unmarried, maternal substance abuse, living with ≥2 children, medical vulnerability, and an interaction term for public aid receipt and Alaska Native ethnicity. Compared with children with 0-1 risk factors, those with 2 (adj. OR = 5.3, 95% CI 3.8, 7.2), 3 (adj. OR = 8.5, 95% CI 6.2, 11.6), or ≥4 risk factors (adj. OR = 16.5, 95% CI 11.9, 22.9) were more likely to have a report. 75% of maltreatment reports were among those with ≥2 risk factors (32% of the population). |
| Parrish (2016)<br><br>USA | **Purpose:** Descriptive<br><br>**Aims:** To investigate the predictive relationship between a maternal prebirth self-reported history of intimate partner violence (IPV) and any post-birth reported allegation to CPS by age 2<br><br>**Outcomes:** CPS report (regardless of screening determination) by age 2 | 1. **Health** (Alaska Pregnancy Risk and Assessment Monitoring System (PRAMS), birth records); **social services** (CPS records)<br>2. Yes<br>3. Yes<br>4. Yes<br>5. 0-2 years | Repeated one-time linkages using probabilistic linkage technique. Linked on infant first and last name and date of birth. Linkage validation/quality assessment ND. | N = 2389 PRAMS respondents 2009-2010. 29.0% Alaska Native. 7.98% had a history of intimate partner violence during or 12 months prior to pregnancy. 8.04% of children had at least one CPS report before age 2. | CPS reports of physical abuse, sexual abuse, neglect, or mental injury (regardless of screening determination) before age 2 [Outcome] | 7.98% of PRAMS respondents self-reported intimate partner violence (IPV) during or in the year leading up to pregnancy (N.B. all percentage estimates are weighted). 8.04% of children had ≥1 CPS report by age 2 (25.2% of children with mothers who had experienced IPV and 6.5% of mothers who had not). Children whose mothers had experience of IPV had a higher likelihood of CPS report (OR = 4.84, 95% CI 3.06, 7.52) than those whose mothers did not; odds were highest for mental injury (OR = 5.25, 95% CI 2.15, 11.78). When stratified by years of education, children whose mothers had completed ≥12 years of education had 3.88 (95% CI 2.25, 6.71) times the risk of CPS report when their mothers had experienced IPV; there was no significant relationship for those with |

| | | | | | | |
|---|---|---|---|---|---|---|
| | | | | | | <12 years of education. |

| | | | | | | |
|---|---|---|---|---|---|---|
| Putnam-Hornstein (2011a)<br><br>USA | **Purpose:** Descriptive<br><br>**Aims:** To examine birth variables that predict which children will be reported for maltreatment by age five<br><br>**Outcomes:** Maltreatment allegation before age 5 | 1. **Health** (vital birth records); **social services** (Department of Social Services CPS records)<br>2. No<br>3. Yes<br>4. No<br>5. 0-5 years (up to 5th birthday) | Retrospective, one-time linkage using probabilistic linkage technique. Linked on unique (Social Security Number) and non-unique (e.g. first name, date of birth) parent and child identifiers. Match rate was 84% with differences by substantiation (substantiated files more likely to be linked) but not type of allegation. | N = 531,035 children born in California in 2002. 48.9% female; 6% Black, 51% Latino/a, 31% White, 12% Asian/Pacific Islander, <0.5% Native American/Alaskan Native. 13.9% had a child protection report (of these, approx. 52% were reported for neglect, 12% for physical abuse, 10% for emotional abuse, 5% for sexual abuse, and 20% were for being at substantial risk of maltreatment) | Child maltreatment allegations [Outcome] | In the full cohort, factors associated with increased risk of CPS report were low birth weight (adj. RR vs. normal birth weight = 1.18, 95% CI 1.15, 1.21), not receiving prenatal care (adj. RR vs. 1st trimester = 1.79, 95% CI 1.72, 1.87), or receiving it in the 2nd/3rd trimester (adj. RRs = 1.20 to 1.29), having a birth abnormality (adj. RR vs. none = 1.07, 95% CI 1.04, 1.10), having a US-born mother (adj. RR vs. foreign-born = 2.13, 95% CI 2.10, 2.17), birth to a Black (adj. RR vs. White mothers = 1.19, 95% CI 1.17, 1.22) or Native American mother (adj. RR = 1.27, 95% CI 1.20, 1.34), maternal age < 30 years (adj. RRs = 1.17 to 2.09 in a dose response relationship with younger age), maternal education less than college degree (adj. RRs= 2.31 to 3.54 in a dose response relationship with less education), maternal history of abortion (adj. RR vs. none = 1.13, 95% CI 1.12, 1.15), missing paternity on birth certificate (adj. RR vs. established = 1.56, 95% CI 1.53, 1.59), family has other children (adj. RRs vs. no other children = 1.40 to 2.33 in dose response relationship with more children), and public insurance for birth (adj. RR vs. other insurance = 1.69, 95% CI 1.66, 1.72). There were significant interaction effects for insurance and maternal race/ethnicity, education, birth place, and age. |

| | | | | | | |
|---|---|---|---|---|---|---|
| Putnam-Hornstein (2013b)<br><br>USA | **Purpose:** Descriptive<br><br>**Aims:** To explore whether aggregate Black/White disparities are largely attributable to the distribution of risk and protective factors at birth. To determine whether the epidemiologic or health paradox observed for Latinos in other domains of health is similarly observed in maltreatment risk among children in California, and whether there were variations among Latinos by maternal nativity.<br><br>**Outcomes:** Referrals and substantiations of maltreatment and out-of-home foster care placement before age 5 | 1. **Health** (vital birth records); **social services** (Department of Social Services CPS records)<br>2. No<br>3. Yes<br>4. No<br>5. 0-5 years (up to 5th birthday) | Retrospective linkage using probabilistic linkage technique. Linked on unique (Social Security Number) and non-unique (e.g. first name, date of birth) parent and child identifiers. Match rate was 84% with differences by substantiation (substantiated files more likely to be linked) but not type of allegation. | N = 531,035 children born in California in 2002. 48.9% female; 6% Black, 51% Latino/a, 31% White, 12% Asian/Pacific Islander, <0.5% Native American/Alaskan Native. 13.9% had a child protection report; 5.2% had a substantiated report. | Child maltreatment (abuse and neglect); referrals and substantiated reports [Outcome] | Before adjusting for confounders, Black children were more than twice as likely as White children to have maltreatment referrals, substantiations, and entry into foster care. In adjusted models, Black children had a significantly lower risk of referral (adj. RR = 0.95, 95% CI 0.92, 0.97), substantiation (adj. RR = 0.79, 95% CI 0.76, 0.83), and entry to foster care (adj. RR = 0.81, 95% CI 0.76, 0.86) compared with White children. Among Latino/a children, before adjustment, those born to foreign-born mothers had lower risk than White children of CPS contact and those born to US-born mothers had higher risk. In adjusted models, Latino/a children (regardless of maternal nativity) had a significantly lower risk of referrals, substantiations, and foster care entry than White children. |

| | | | | | | |
|---|---|---|---|---|---|---|
| Van Horne (2018)<br><br>USA | **Purpose:** Descriptive<br><br>**Aims:** To determine whether the prevalence of maltreatment decreased with age in children with birth defects, and whether differences in maltreatment between birth defect groups remained as children aged. To identify factors at the child-, family-, and neighbourhood-levels associated with an increased risk of maltreatment in children over age 2<br><br>**Outcomes:** Substantiated maltreatment from age 2-10 | 1. **Health** (Texas Department of State Health Services Vital Statistics Unit birth and death records, Texas Birth Defects Registry); **social services** (Texas Department of Family and Protective Services (CPS) records); **other aggregate** (American Community Survey)<br>2. No<br>3. Yes<br>4. Yes<br>5. 0-10 years (maltreatment measured 2-10 years) | Multiple one-time linkages using probabilistic linkage technique. Linked on parent and child names, dates of birth, and social security numbers. Data were de-identified. Implied exemption from active consent. 85.8% of CPS records and >98% of birth defect records were matched to birth certificates. | N = 2,902,385 children born in Texas 2002-2009 without substantiated maltreatment before age 2. 49.3% female; maternal ethnicity 49.8% Hispanic, 35.0% White non-Hispanic, 10.9% Black non-Hispanic, 4.2% other non-Hispanic. 2.9% of unaffected children, 2.8% of children with Down Syndrome, 4.3% of children with cleft lip +/- cleft palate, and 2.7% of children with spina bifida had a substantiated maltreatment report (approx. 75-85% were for neglectful supervision). | Substantiated maltreatment (neglectful supervision, physical abuse, physical neglect, medical neglect, sexual abuse, abandonment, emotional abuse, refusal to accept parental responsibility; could be perpetrated by someone other than a parent) after age 2 [Outcome] | Maltreatment generally decreased from age 2-10. Children with Down Syndrome (adj. HR = 1.32, 95 CI 1.06, 1.64) and cleft lip with or without cleft palate (adj. HR = 1.26, 95% CI 1.01, 1.56), but not children with spina bifida, were significantly more likely than those with no birth defects to have a substantiated maltreatment report. For the full group (with and without birth defects), risk factors for maltreatment were prematurity (adj. HR vs. full-term = 1.10, 95% CI 1.07, 1.14), low birth weight (adj. HR vs. normal birth weight = 1.20, 95% CI 1.16, 1.24), higher parity (adj. HRs = 1.42 to 2.37, in a dose-response relationship with higher parity), teenage motherhood (adj. HR vs. 20-34 years = 1.69, 95% CI 1.66, 1.72), unmarried mothers (adj. HR =1.66, 95% CI 1.63, 1.69), maternal education less than high school (adj. HRs = 1.84 to 2.46 in a dose-response relationship for less education), public insurance at birth (adj. HR vs. non-public insurance = 2.64, 95% CI 2.59, 2.68), missing paternity (adj. HR = 1.34, 95% CI 1.31, 1.36), and high neighbourhood impoverishment (adj. HR = 1.09, 95% CI 1.07, 1.12). Lower risk of maltreatment was associated multiple birth (adj. HR = 0.47, 95% CI 0.44, 0.50), maternal ethnicity other than White non-Hispanic (adj. HRs = 0.39 to 0.70), and older maternal age (adj. HR vs. 20-34 years = 0.51, 95% CI 0.49, 0.53). |

| | | | | | | |
|---|---|---|---|---|---|---|
| Van Horne (2015)<br><br>USA | **Purpose:** Descriptive<br><br>**Aims:** To determine whether the risk and predictors of maltreatment differ between children with and without 3 birth defects: Down syndrome, cleft lip with/without cleft palate, and spina bifida<br><br>**Outcomes:** Substantiated maltreatment aged 4 days to 2 years | 1. **Health** (Texas Department of State Health Services Vital Statistics Unit birth and death records, Texas Birth Defects Registry); **social services** (Texas Department of Family and Protective Services (CPS) records); **other aggregate** (American Community Survey)<br>2. No<br>3. Yes<br>4. Yes<br>5. 0-2 years | Multiple one-time linkages using probabilistic linkage technique. Linked on parent and child names, dates of birth, and social security numbers. Data were de-identified. Implied exemption from active consent. 85.8% of CPS records and >98% of birth defect records were matched to birth certificates. | N = 2,977,758 children born in Texas 2002-2009 without substantiated maltreatment before age 2. 49.3% female; maternal ethnicity 49.6% Hispanic, 35.1% White non-Hispanic, 11.1% Black non-Hispanic, 4.1% other non-Hispanic. 2.3% of unaffected children, 2.0% of children with Down Syndrome, 3.6% of children with cleft lip +/- cleft palate, and 3.8% of children with spina bifida had a substantiated maltreatment report. | Substantiated maltreatment (neglectful supervision, physical abuse, physical neglect, medical neglect, sexual abuse, abandonment, emotional abuse, refusal to accept parental responsibility; could be perpetrated by someone other than a parent) [Outcome] | Children with cleft lip with or without cleft palate (adj. RR = 1.40, 95% CI 1.14, 1.71) and spina bifida (adj. RR = 1.58, 95% CI 1.12, 2.24), but not Down Syndrome, were more likely than children without a birth defect to have substantiated maltreatment by age 2. Children with any birth defect were more likely than those without to have a report of medical neglect (adj. RRs = 3.6 to 62.) For the full group (with and without birth defects), risk factors for maltreatment were prematurity (adj. HRs for premature and very premature vs. full-term = 1.18 to 1.28), low birth weight (adj. HR vs. normal birth weight = 1.39, 95% CI 1.34, 1.43), higher parity (adj. HRs = 1.55 to 3.17, in a dose-response relationship with parity), teenage motherhood (adj. HR vs. 20-34 years = 1.64, 95% CI 1.61, 1.68), unmarried mothers (adj. HR =1.83, 95% CI 1.79, 1.87), maternal education less than high school (adj. HRs = 1.79 to 2.43 in a dose-response relationship for less education), public insurance at birth (adj.HR vs. non-public insurance = 2.71, 95% CI 2.66, 2.76), missing paternity (adj. HR = 1.52, 95% CI 1.49, 1.55), and high neighbourhood impoverishment (adj. HR = 1.12, 95% CI 1.09, 1.15). Lower risk of maltreatment was associated multiple birth (adj. HR = 0.55, 95% CI 0.52, 0.58), maternal ethnicity other than White non-Hispanic (adj. HRs = 0.42 to 0.62), and older maternal age (adj. HR vs. 20-34 years = 0.54, 95% CI 0.52, 0.56). |
| **Outcomes** | | | | | | |

| | | | | | | |
|---|---|---|---|---|---|---|
| Abajobir (2017a)<br><br>Australia | **Purpose:** Descriptive<br><br>**Aims:** To examine the independent effect of single and multiple forms of substantiated child maltreatment on quality of life<br><br>**Outcomes:** Quality of life | 1. **Social service**s (CPS records); **study-specific** (Mater Hospital-University of Queensland Study of Pregnancy measures incl. measures of quality of life)<br>2. Yes<br>3. Yes<br>4. Yes<br>5. 0-21 years (data on maltreatment until age 14) | Repeated one-time/near real-time linkage. Linkage technique (probabilistic vs. deterministic) ND. Linked on unique identification numbers. Active consent for linkage. Identifiable data. Linkage validation/quality assessment ND. | N = 3730 young people from the Mater Hospital-University of Queensland Study of Pregnancy (51.6% of original sample). 49.7% female; mean age = 20.6 years. 4.7% had substantiated reports of child maltreatment. (N.B. 65.6% of original sample with substantiated child maltreatment reports were lost to follow-up) | Substantiated child maltreatment reports (including physical abuse, sexual abuse, emotional abuse, and neglect) [Exposure] | Compared with those who without substantiated child maltreatment reports, those who had experienced emotional abuse and neglect had a lower quality of life (adj. ORs = 2.49, 95% CI 1.41, 4.39 and 2.86, 95% CI 1.53, 5.36, respectively). Sexual and physical abuse were not significantly correlated in the adjusted model. In terms of combined maltreatment measures, having any substantiated child maltreatment report was associated with lower quality of life (adj. OR = 2.08, 95% CI 1.37, 3.16), as was emotional abuse with or without neglect (adj. OR = 3.34, 95% CI 2.06, 5.41). Sexual abuse with or without physical abuse was not significantly associated with lower quality of life in the adjusted model. |

| | | | | | | |
|---|---|---|---|---|---|---|
| Abajobir (2017b)<br><br>Australia | **Purpose:** Descriptive<br><br>**Aims:** To quantify the association between co-occurring and specific forms of substantiated childhood maltreatment and high fat intake-related behaviours in adulthood and whether there is an interaction with gender. To examine the association between age and number of childhood maltreatment substantiations and subsequent high dietary fat intake.<br><br>**Outcomes:** High dietary fat intake behaviours at age 21 | 1. **Social services** (CPS records); **study-specific** (Mater Hospital-University of Queensland Study of Pregnancy measures incl. Short Fat Questionnaire)<br>2. Yes<br>3. Yes<br>4. Yes<br>5. 0-21 years (data on maltreatment until age 14) | Repeated one-time linkages using probabilistic linkage technique. Mothers' records linked on personally identifiable maternal data from the birth record; infants' data matched on personally identifiable data. Parents/children provided consent for follow-ups (parents until age 14 follow-up and adolescents and parents at ages 14 and 21). Linkage validation/quality assessment ND. | N = 3766 young people from the Mater Hospital-University of Queensland Study of Pregnancy (52.1% of original sample). 47.4% female; mean age = 20.6 years. 4.5% had substantiated reports of child maltreatment (N.B. 66.8% of original sample with substantiated child maltreatment reports were lost to follow-up) | Substantiated CPS reports (including physical abuse, sexual abuse, emotional abuse and neglect) [Exposure] | Participants with any substantiated child maltreatment report were significantly more likely than those without to have high dietary fat intake (adj. OR = 1.65, 95% CI 1.06, 2.57). By category, only physical abuse was significantly associated with having high dietary fat intake (adj. OR = 1.91, 95% CI 1.04, 3.49); sexual abuse, emotional abuse, and neglect were not significantly associated with having a high dietary fat intake. Exposure at an older age (5-14 years) was associated with a greater likelihood of high fat intake compared with at a younger age (0-4 years; adj. OR = 1.77, 95% CI 1.08, 2.89). Those with more than one substantiated report had a greater likelihood of high fat intake than those with only one report (adj. OR = 1.41, 95% CI 1.05, 1.89). |

| | | | | | | |
|---|---|---|---|---|---|---|
| Boyd (2019)<br><br>Australia | **Purpose:** Descriptive<br><br>**Aims:** To examine whether child maltreatment is associated with attentional problems in adolescence and young adulthood, and whether outcomes depend on the type of maltreatment<br><br>**Outcomes:** Attentional problems at ages 14 and 21 years | 1. **Social services** (Department of Families, Youth and Community Care (CPS) records); **study-specific** (Mater Hospital-University of Queensland Study of Pregnancy measures incl. Child Behaviour Checklist, Youth Self-Report, and Young Adult Self-Report)<br>2. Yes<br>3. Yes<br>4. Yes<br>5. 0-21 years | Retrospective, one-time linkage. Linkage technique (probabilistic vs. deterministic) ND. Linked on unique identification numbers. Mothers gave consent for linkage. Data were shared with researchers in anonymised format. Linkage validation/quality assessment ND. | N = 7214 mother-child dyads, of whom 72% completed the age 14 follow-up and 52.4% of whom completed the age 21 follow-up. 9.9% of children had a non-sexual abuse CPS notification and 3.6% had a sexual abuse CPS notification. | Child protection notifications of physical, sexual, or emotional abuse, and neglect (before the age of 14) [Exposure] | Childhood factors included in bivariate analyses associated with increased likelihood of CPS notification for physical abuse, emotional abuse, or neglect included being Indigenous Australian, low family income, parents not living together, mother not completing high school, chronic maternal depression, and low birthweight ($p < 0.001$). For sexual abuse, being Indigenous Australian was not significantly associated with CPS notification, but being female was ($p < 0.001$). Compared with those who had not, those who had experienced non-sexual maltreatment had a greater likelihood of attentional problems at age 14 and 21 ($p < 0.001$ and $p = 0.044$, respectively in adjusted models). Compared with those who had not, those who had experienced sexual maltreatment did not have a significantly higher chance of attentional problems at age 14 or 21 on self-report measures. |
| Cederbaum (2013)<br><br>USA | **Purpose:** Descriptive<br><br>**Aims:** To examine the maltreatment history of adolescent mothers as an independent predictor of infant birth weight<br><br>**Outcomes:** Infant birth weight | 1. **Health** (vital birth records); **social services** (CPS records)<br>2. No<br>3. Yes<br>4. Yes<br>5. Mothers: 12-19 years (child maltreatment measured after age 10 and before giving birth only) | Retrospective, one-time linkage using probabilistic linkage techniques. Linkage validation/quality assessment ND. | N = 153,762 births to teenage mothers (12-19 years old); maternal race/ethnicity 72.4% Latina, 13.6% White, 8.5% Black, 2.5% Asian/Pacific Islander, 0.5% Native American. 13.6% had a substantiated child maltreatment report (after age 10 and before giving birth) | Mothers' substantiated CPS reports of child maltreatment (abuse or neglect) after age 10 and before giving birth [Exposure] | Maternal history of child maltreatment was significantly associated with an increased risk of low birth weight in their infants (adj. RR = 1.06, 95% CI 1.01, 1.12). |

| | | | | | | |
|---|---|---|---|---|---|---|
| Coulton (2016)<br><br>USA | **Purpose:** Descriptive<br><br>**Aims:** To examine the influence of housing and neighbourhood conditions on school readiness<br><br>**Outcomes:** Kindergarten readiness (literacy skills) | 1. ChildHood Integrated Longitudinal Data (CHILD) system: **health** (public health); **social services** (public assistance and social services agencies data, early childhood programmes); **education**; **other aggregate data** (geographic information system data)<br>2. No<br>3. Yes<br>4. ND<br>5. 0-5 years | Retrospective, one-time linkage of two sources (integrated data systems). First source is a linked dataset using probabilistic linkage technique (multiple retrospective linkages. Probabilistic, retrospective linkage. Linkage validation/quality assessment ND. | All children entering kindergarten in Cleveland in 2007-2010. Mean age at entry = 65.6 months (SD 3.9); 51.5% female; 69.0% African American, 18.2% Non-Hispanic White, 11.7% Hispanic, 1.1% other. | Child abuse/neglect investigations before kindergarten entry [Exposure (covariate)] | Children with maltreatment reports score significantly lower on school readiness than do their peers. Higher proportions of time spent in poverty and certain adverse housing conditions (e.g. in poor condition, in foreclosure, public housing) were significantly associated with greater likelihood of child maltreatment report. Concentrated neighbourhood disadvantage, low market value, tax delinquency, and speculator ownership of housing were not associated with greater likelihood of child maltreatment reports. |
| Cram (2015)<br><br>New Zealand | **Purpose:** Descriptive<br><br>**Aims:** To investigate the high representation of Māori children in child welfare data<br><br>**Outcomes:** Infant mortality | 1. **Health** (birth notifications and registrations, Ministry of Health publicly-funded maternity services, publicly-funded hospitalisations, and mortality records); **justice** (corrections sentences); **social services** (Children, Youth and Family (CPS) records, Ministry of Social Development public welfare benefits)<br>2. No<br>3. Yes<br>4. Yes<br>5. 0-3 years | Retrospective, one-time linkage using probabilistic linkage technique. Clerical review of a subsample of linkages found good overall accuracy for children, but lower accuracy for adults. | All live births from July 1, 2004, to June 30, 2007 (N = 180, 794); 29.1% Māori. For Māori children, 22.1% had a notification or police family violence contact record, 7.6% had a substantiated finding of maltreatment: 0.04% substantiated sexual abuse, 0.6% physical abuse, 3.1% neglect, 5.5% emotional abuse. For non-Māori, non-Pacific children, 5.7% had notification or police family violence contact record, 1.5% had a substantiated finding of maltreatment: 0.01% substantiated sexual abuse, 0.2% physical | Notifications, investigations, and substantiations of child maltreatment (sexual abuse, physical abuse, emotional abuse, neglect) [Outcome] | Rate ratios for all indicators of child maltreatment were >1 (generally >3.0), indicating greater rates for Māori children. However, rate ratios were attenuated when stratified by duration on welfare benefits (0 of the last 5 years vs. ≥4) and maternal age (<25 vs. 25+). |

| | | | | | | |
|---|---|---|---|---|---|---|
| | | | | abuse, 0.7% neglect, 1.0% emotional abuse. | | |
| Cutajar (2010a)<br><br>Australia | **Purpose:** Descriptive<br><br>**Aims:** To determine the rate and risk of clinical and personality disorders diagnosed in childhood and adulthood for those exposed to childhood sexual abuse<br><br>**Outcomes:** Contact with public mental health services and psychiatric diagnoses | 1. **Health** (Victorian Psychiatric Case Register, Victoria Institute of Forensic Medicine records (medical examinations of all cases of suspected sexual abuse))<br>2. No<br>3. Yes<br>4. No<br>5. Sexual abuse occurred from 0-16 years; follow-up 12-43 years | Retrospective, one-time linkage using deterministic and probabilistic linkage techniques.<br>Linked on identifying information including surname, first name, date of birth or age range, gender. No consent requirement to access information. Data anonymised post-linkage. Linkage 'checked for accuracy' (no further detail provided). | N = 2688 cases of childhood sexual abuse. 80.1% females; mean age at examination = 10.17 years (SD 4.46) mean age at follow-up 33.68 years (SD 11.05). Abuse was primarily penetrative (63.2%), by one offender (94.4%), on more than one occasion (61.9%); over half were abused by someone outside of the family (51.9%). | Sexual abuse (could be by someone not related to the child); cases identified on the basis of the history provided by the child or other informants and examination findings/laboratory analysis [Exposure] | 23.3% of those with a history of sexual abuse had a lifetime record of contact with public mental health service (vs. 7.7% of controls) (adj. OR = 3.65, 95% CI, 3.09–4.32, $p < 0.001$). History of sexual abuse accounted for approx. 7.83% of mental health service contact (6.21% for males, 1.62% for females). Those with a history of sexual abuse had a significantly higher likelihood than those without of childhood or adulthood diagnosis of psychosis, affective, post-traumatic stress disorder, other anxiety disorders, substance abuse, and personality disorders (but not eating disorders), with older age at abuse and greater severity of abuse associated with greater risk of psychopathology. When examined by gender, females who had experienced sexual abuse had significantly higher risk than those who had not of all disorders except eating disorders, whereas the increased risk affective disorders, post-traumatic stress disorder, non-cluster B personality disorders, and borderline personality disorders ceased to be significant for males. |

| | | | | | | |
|---|---|---|---|---|---|---|
| Cutajar (2010b)<br><br>Australia | **Purpose:** Descriptive<br><br>**Aims:** To explore whether child sexual abuse is a risk factor for later psychotic disorders<br><br>**Outcomes:** Psychotic disorders | 1. **Health** (Victorian Psychiatric Case Register, Victoria Institute of Forensic Medicine records (medical examinations of all cases of suspected sexual abuse))<br>2. No<br>3. Yes<br>4. No<br>5. Sexual abuse occurred from 0-16 years; follow-up 13-44 years | Retrospective, one-time linkage using deterministic and probabilistic linkage techniques.<br>Linked on identifying information including surname, first name, date of birth or age range, gender. No consent requirement to access information. Data anonymised post-linkage. Linkage 'checked for accuracy' (no further detail provided). | N = 2759 cases of childhood sexual abuse. 79.8% females; mean age at examination = 10.22 years (SD 4.4); mean age at follow-up 33.68 years (SD 11.05). Abuse was primarily penetrative (63%) and with higher rates for females than males (64.9% vs. 55.2%; p<0.001). | Sexual abuse (could be by someone not related to the child); cases identified on the basis of the history provided by the child or other informants and examination findings/laboratory analysis [Exposure] | 25.3% of those with a history of sexual abuse had a lifetime record of contact with public mental health service (vs. 7.7% of controls) (adj. OR = 4.1, 95% CI 3.4–4.8, p < 0.001). Compared with controls, those with a history of sexual abuse were more likely to develop any psychosis (2.8% vs. 1.4%, adj. OR = 2.1, 95% CI 1.4, 3.1, p<0.001) or schizophrenia (1.9% vs. 0.7%, adj. OR = 2.6, 95% CI 1.6, 4.4, p<0.001), but not non-schizophrenic psychotic disorders. Penetrative abuse was associated with an even higher rate of any psychosis (3.4%) and schizophrenia (2.4%) while non-penetrative abuse was not significantly associated with increased risk. The highest risk of psychosis existed for those with the most severe cases, i.e. those with penetrative abuse that occurred at an older age, and involved more than one perpetrator (17.2% for any psychosis, 8.6% for schizophrenia; adj. ORs compared with peers without sexual abuse history = 14.9, 95% CI 8.4, 26.3 for any psychosis and 12.5, 95% CI 3.4, 45.3 for schizophrenia). |

| | | | | | | |
|---|---|---|---|---|---|---|
| Dahl (2017)<br><br>Denmark | **Purpose:** Descriptive<br><br>**Aims:** To examine the individual and cumulative effects of 9 different types of early adversity on risk for moderate to severe unipolar depression in adolescence and adulthood<br><br>**Outcomes:** Moderate to severe unipolar depressive disorder diagnosis | 1. **Health** (Danish National Patient Register, Danish Psychiatric Central Research Register (inpatient, outpatient, and emergency admissions), Danish Register of Causes of Death); **justice** (Danish National Crime Register); **other** (Danish Register on Personal Labour Market Affiliation)<br>2. No<br>3. Yes<br>4. Yes<br>5. 0-34 years; abuse measured from age 0-15 years | Retrospective, one-time linkage using deterministic linkage technique (within a system using national registries). Linked on personal ID number. Implied exemption from active consent. Linkage validation/quality assessment ND. | All live births in Denmark between 1990 and 1998 (N = 978,674). 48.7% female. N = 1004 (0.1%) had a diagnosis of abuse (0.07% of males and 0.14% of females) (*N.B.* abuse diagnoses only available from 1992 onwards). | ICD diagnosis of physical abuse, sexual abuse, psychological abuse, neglect or abandonment, other maltreatment syndrome, or maltreatment syndrome, unspecified before age 15 [Exposure] | Diagnosis of childhood abuse was significantly associated with depression diagnosis (unadj. HR = 2.72, 95% CI 1.95, 3.80, p<0.001; HR adj. for other adversities = 1.70, 95% CI 1.22, 2.38, p = 0.002). |

| | | | | | | |
|---|---|---|---|---|---|---|
| Font (2019)<br><br>USA | **Purpose:** Descriptive<br><br>**Aims:** To assess differences in the risk of early motherhood among low-income, maltreated, and foster girls and investigate whether differences likely reflect selection factors versus effects of involvement with CPS or foster care<br><br>**Outcomes:** Early motherhood | 1. Wisconsin Multi-Sample Person File: **Education** (Department of Public Instruction); **health** (Medicaid); **justice** (state prisons); **social services** (State-Administered Child Welfare Information System (CPS records), Supplemental Nutrition Assistance Program (food stamps), Temporary Assistance to Needy Families (TANF/cash welfare))<br>2. No<br>3. Yes<br>4. Yes<br>5. 0-18 years | Retrospective, one-time linkage using probabilistic linkage technique (from a larger 'living' research database). Implied exemption from consent requirements. Linkage validation/quality assessment ND. | N = 71,824 teenage girls born 1991-1996 (N = 48,915 SNAP (low income) alone, 18,869 CPS but not foster care, N = 4,040 foster care). SNAP girls: 52.0% White, 25.8% Black, 10.8% Hispanic, 6.8% Asian, 2.4% American Indian, 1.8% Multiracial, 0.5% other/unknown. CPS girls: 57.0% White, 23.7% Black, 9.3% Hispanic, 2.2% Asian, 3.0% American Indian, 2.3% Multiracial, 2.5% other/unknown. Foster care girls: 52.4% White, 29.7% Black, 8.1% Hispanic, 1.9% Asian, 4.3% American Indian, 3.3% Multiracial, 0.4% other/unknown. | CPS investigations between 2004 and 18th birthday (N.B. this means some girls will not have CPS records from before age 13) [Exposure] | Girls with CPS investigations/foster care experience had significantly higher risk for early motherhood compared with those whose families received SNAP only (adj. HR = 2.00 and 2.54 for CPS and foster care, respectively; p<0.001). These girls also had significantly greater risk of early motherhood than the SNAP group at all timepoints (before, during, and after CPS investigation/foster care placement). CPS risk was highest before investigation (adj. HR = 3.0 vs. 1.9 for before vs. during/after CPS investigation; adj. HR = 3.6 vs. 2.7 vs. 1.7 for before vs. after vs. and during foster care placement; p<0.001). |

| | | | | | | |
|---|---|---|---|---|---|---|
| Font (2020a)<br><br>USA | **Purpose:** Descriptive<br><br>**Aims:** To estimate the 'added harm' of CPS-investigated neglect, net of poverty exposure (depth and duration), on high school completion, employment and earnings, incarceration, and teen parenthood. To assess whether abuse is a stronger risk factor for adverse outcomes than neglect.<br><br>**Outcomes:** High school completion, employment and earnings, incarceration, teen parenthood | 1. Wisconsin Administrative Data Core (WADC; linked with additional data): **Education** (Department of Public Instruction); **health** (Medicaid); **justice** (Department of Corrections, Milwaukee County Jail); **social services** (child welfare (CPS records), Supplemental Nutrition Assistance Program (SNAP; food stamps), Unemployment Insurance system (including earning data), Supplemental Security Income programme records, cash welfare, child support records)<br>2. No<br>3. Yes<br>4. Yes<br>5. 0-20th birthday (maltreatment records 0-16 years) | Retrospective, one-time linkage using deterministic and probabilistic linkage techniques (from a larger research database that is routinely updated). Implied exemption from consent requirements. Manual checks of false matches (described in detail on the WADC website). | N = 29,154 children who received SNAP (no maltreatment) or had a CPS allegation before age 16.<br><br>No maltreatment group: N = 19,876. 48.4% female; 14.7% White non-Hispanic, 55.3% Black non-Hispanic, 17.9% Hispanic (any race), 12.2% other/unknown race.<br><br>Neglect only group: N = 3060. 47.4% female; 18.6% White non-Hispanic, 59.7% Black non-Hispanic, 11.4% Hispanic (any race), 10.3% other/unknown race.<br><br>Abuse only group: N = 2248. 53.0% female; 22.5% White non-Hispanic, 53.2% Black non-Hispanic, 15.6% Hispanic (any race), 8.7% other/unknown race.<br><br>Abuse & neglect group: N = 3970. 53.3% female; 22.3% White non-Hispanic, 51.0% Black non-Hispanic, 15.4% Hispanic (any race), 11.3% other/unknown race. | Allegations and substantiations of abuse (physical, sexual, emotional) and neglect before age 16 years, categorised as follows: no maltreatment (NM), alleged or confirmed neglect only (NO), alleged or confirmed abuse only (AO), and alleged or confirmed abuse and neglect (AN) [Exposure] | Compared with those with no maltreatment allegations, all youth with maltreatment allegations had lower odds of high school graduation and regular employment, lower average earnings, and higher odds of teen parenthood and incarceration. Youth with allegations of abuse AND neglect had lower odds of high school graduation and higher odds of teen parenthood than those with only abuse OR neglect allegations. Youth with only abuse allegations had higher odds of incarceration and those with neglect only allegations had lower odds of stable employment. No sig. differences between abuse only and neglect only in terms of odds of high school graduation, teen parenthood, or earnings. Probabilities of each outcome (high school graduation, teen parenthood, stable employment, average earnings, incarceration in jail, and incarceration in prison) varied by outcome, though the general trend was that the NM group had the highest probability of a positive outcome, followed by AO and NO (similar), and AN had the lowest probability of a positive outcome; outcomes improved with lower poverty exposure (depth and duration). Outcomes were worse for the groups with maltreatment allegations regardless of CPS intervention. |

| | | | | | | |
|---|---|---|---|---|---|---|
| ßGreen (2018a)<br><br>Australia | **Purpose:** Descriptive<br><br>**Aims:** To determine classes of children who may be at risk for later mental health disorder and associated exposures in a population cohort<br><br>**Outcomes:** Risk for developing an adult mental health disorder | 1. New South Wales - Child Development Study (NSW-CDS): **education** (Australian Government Department of Education Australian Early Development Census (AEDC)); **health** (NSW Registry of Births, Deaths and Marriages Birth Registrations, Death Registrations, NSW Ministry of Health's Perinatal Data Collection, Admitted Patient Data Collection, Emergency Department Data Collection, Mental Health Ambulatory data collection); **justice** (NSW Bureau of Crime Statistics and Research Reoffending data); **social services** (NSW Family and Community Services Child Protection Case Management System – Key Information Directory System (CPS) records)<br>2. No<br>3. Yes<br>4. Yes<br>5. 0-5 years | Repeated one-time linkages using probabilistic linkage technique (data from data linkage centre/linked data resource) – study used dataset created with a one-time retrospective linkage. Linked on name, date of birth, residential addresses, and sex. Data were de-identified. No consent requirements. Low rates of false-positive linkages for children's data (0.3%) and for linkage of parents to children (0.5%). | N = 67,353 children enrolled in the New South Wales Child Development Study. 49.5% female; mean age = 5.6 years (SD 0.4). 3.1% had at least one substantiated childhood maltreatment report. Of these, 46.9% had experienced emotional abuse, 26.2% neglect, 16% physical abuse, 11% sexual abuse; of those with more than one type of maltreatment, the most common combination was emotional abuse and neglect (39.6%) followed by physical and emotional abuse (18.9%), physical abuse and neglect (11.3%), emotional and sexual abuse (9.1%), physical and sexual abuse (3.0%), sexual abuse and neglect (2.4%) or the combination of sexual, physical and emotional abuse (1.7%); less than 1% of children experienced all four types of maltreatment. | Substantiated reports of childhood maltreatment (sexual abuse, physical abuse, emotional abuse, neglect) [Exposure (covariate)] | Four classes of risk for adult mental health disorders were identified: (1) disrespectful and aggressive/hyperactive behaviour, labelled 'misconduct risk' (6.5% of children); (2) 'pervasive risk' (4.0% of children); (3) 'mild generalised risk' (1.6% of children); and (4) 'no risk' (77.9% of children). Membership to classes 1-3 was predicted by children with experience of maltreatment, parental history of mental health disorders, parental history of criminal offending, socioeconomic disadvantage, and perinatal adversities. Compared with those in the 'no risk' group, odds of maltreatment were highest for 'pervasive risk' class (adj. OR = 6.35, 95% CI 5.45, 7.40) followed by the 'misconduct risk' and 'mild generalised risk' classes (adj. OR = 4.43, 95% CI 3.80, 5.15 and adj. OR = 3.41, 95% CI 2.97, 3.92, respectively). These relationships were attenuated with additional covariates (incl. parental mental health disorders, parental history of offending, and perinatal risk factors); in the final model, ORs for maltreatment relative to the 'no risk' class were 2.84 (95% CI 2.40, 3.35), 2.34 (95% CI 1.99, 2.75), and 1.93 (95% CI 1.66, 2.24) for the 'misconduct risk', 'pervasive risk', and 'mild generalised risk,' classes, respectively. |

| | | | | | | |
|---|---|---|---|---|---|---|
| Green (2018b)<br><br>Australia | **Purpose:** Descriptive<br><br>**Aims:** To estimate (a) associations between any exposure to child maltreatment and developmental vulnerability at age 5, (b) the relative strength of association between developmental vulnerabilities and exposure to multiple types of maltreatment versus a single type of maltreatment, and (c) the relative strength of associations between differential timing of first reported maltreatment on any single or multiple domains of developmental vulnerability at age 5.<br><br>**Outcomes:** Developmental vulnerability on five domains of functioning at age 5 (including social, emotional, physical, cognitive, and communication competencies) | 1. New South Wales - Child Development Study (NSW-CDS): **education** (Australian Government Department of Education Australian Early Development Census (AEDC)); **health** (NSW Registry of Births, Deaths and Marriages Birth Registrations, Death Registrations, NSW Ministry of Health's Perinatal Data Collection, Admitted Patient Data Collection, Emergency Department Data Collection, Mental Health Ambulatory data collection); **justice** (NSW Bureau of Crime Statistics and Research Reoffending data); **social services** (NSW Family and Community Services Child Protection Case Management System – Key Information Directory System (CPS) records and additional data sets pertaining to allocation to a family remedial program and out-of-home care) | Repeated one-time linkages using probabilistic linkage technique (data from data linkage centre/linked data resource) – study used dataset created with a one-time retrospective linkage. Linked on name, date of birth, residential addresses, and sex. Data were de-identified. No consent requirements. Low rates of false-positive linkages for children's data (0.3%) and for linkage of parents to children (0.5%). | N = 68,459 children enrolled in the New South Wales Child Development Study. 49.5% female; mean age = 5.6 years (SD 0.37). 3.1% had at least one substantiated childhood maltreatment report. Of these, 46.6% had experienced emotional abuse, 26.3% neglect, 16.1% physical abuse, 11% sexual abuse; of those with more than one type of maltreatment, the most common combination was emotional abuse and neglect (39.5%) followed by physical and emotional abuse (19.1%), physical abuse and neglect (11.3%), emotional and sexual abuse (9.1%), physical and sexual abuse (3.0%), sexual abuse and neglect (2.3%) or the combination of sexual, physical and emotional abuse (1.7%); less than 1% of children experienced all four types of maltreatment. | Substantiated reports of childhood maltreatment (sexual abuse, physical abuse, emotional abuse, neglect) [Exposure] | Children exposed to any maltreatment were more likely to be vulnerable on each of the 5 domains of the AEDC: adj. ORs (95% CIs) for social, emotional, physical, cognitive, and communication domains were 2.28 (2.01, 2.58), 2.26 (1.95, 2.58), 2.11 (1.87, 2.39), 2.50 (2.19, 2.86), and 2.13 (1.87, 2.44), respectively. Significant associations with 'at-risk' status were also found across all domains. Adj. OR for vulnerability on ≥3 domains was 3.38 (95% CI 2.89, 3.96), compared with those without exposure to maltreatment. Factors associated with greater likelihood included being exposed to multiple types of maltreatment and older age at reported maltreatment (>3 years). |

| | | | | | | |
|---|---|---|---|---|---|---|
| | | 2. No<br>3. Yes<br>4. Yes<br>5. 0-5 years | | | | |
| Green (2019a)<br><br>Australia | **Purpose:** Descriptive<br><br>**Aims:** To examine associations between developmental vulnerability profiles determined at age 5 years and subsequent childhood mental illness between ages 6 and 13 years in an Australian population cohort<br><br>**Outcomes:** Childhood mental health disorders and treatment | 1. New South Wales - Child Development Study (NSW-CDS): **education** (Australian Government Department of Education Australian Early Development Census (AEDC)); **health** (NSW Ministry of Health Mental Health Emergency Department Data Collection, Admitted Patients Data Collection, Mental Health Ambulatory records); **social services** (Department of Family & Community Services (CPS) records)<br>2. No<br>3. Yes<br>4. Yes<br>5. 0-13 years | Repeated one-time linkages using probabilistic linkage technique (data from data linkage centre/linked data resource) – study used dataset created with a one-time retrospective linkage. Linked on name, date of birth, residential addresses, and sex. Data were de-identified. No consent requirements. Low rates of false-positive linkages for children's data (0.3%) and for linkage of parents to children (0.5%). | N = 82,891 children enrolled in the New South Wales - Child Development Study. 49.2% female; mean age = 13.15 years (SD 0.4). N = 20,741 (23.9%) had a child protection contact. | Child protection report or out-of-home placement prior to age 13 [Exposure (covariate)] | Children with child protection contact had at least double the risk of those without contact of all mental health disorders/behaviours studied, with largest risk for self-harm (adj. OR = 7.66, 95% CI 4.55, 12.88), conduct disorders (adj. OR = 5.82, 95% CI 4.35, 7.79), stress reactions (adj. OR = 5.40, 95% CI 3.82, 7.64), and hyperkinetic disorders (adj. OR = 3.53, 95% CI 2.58, 4.81). Child protection contact was also related to more days of mental health treatment (RR = 1.56, 95% CI 1.35, 1.81). |

| | | | | | | |
|---|---|---|---|---|---|---|
| Gwaltney (2019)<br><br>USA | Study 1: **Purpose:** Descriptive<br><br>**Aims:** To examine the association between individual and contextual-level factors and medication use among children in foster care<br><br>**Outcomes:** psychotropic medication prescription<br><br>Study 2: **Purpose:** Descriptive<br><br>**Aims:** To examine the effect of psychotropic medication use on three foster care outcomes: time to exit, exit type, and placement stability<br><br>**Outcomes:** foster care outcomes: time to exit, exit type, placement stability | 1. **Health** (North Carolina Medicaid claims database); **social services** (CPS records)<br>2. No<br>3. Yes<br>4. No<br>5. 0-19 years | Retrospective, one-time linkage. Linkage technique (probabilistic vs. deterministic) ND. Linkage validation/quality assessment ND. | Study 1:<br>N = 30,657 children in foster care. 49.7% female; 48.3% White, 34.1% Black, 8.4% Hispanic, 9.2% Other. 8.8% had experienced physical abuse, 3.9% sexual abuse, and 79.8% neglect.<br><br>Study 2:<br>N = 14,799 children in foster care. 51.4% female; 49.2% White, 34.9% Black, 8.0% Hispanic, 7.5% Other. 7.8% had experienced physical abuse, 5.9% sexual abuse, and 75.5% neglect. | Child maltreatment resulting in removal from home (incl. physical abuse, sexual abuse, neglect, or 'other') [Exposure] | Study 1: Stimulants, antidepressants, and antipsychotics were the most commonly prescribed medication classes across all maltreatment histories. Rates of any medication use were 30.0% for those with experience of sexual abuse, 21.4% for physical abuse, and 19.4% for neglect.<br><br>Study 2: Maltreatment history type was not significantly related to the number of placements or exit type (reunification, guardianship, adoption). |

| | | | | | | |
|---|---|---|---|---|---|---|
| Hu (2017)<br><br>Australia | **Purpose:** Descriptive<br><br>**Aims:** To investigate longitudinally the effects of the number, timing, and type of maltreatment allegations on adolescent risk of having a deliberate self-harm-related hospital admission<br><br>**Outcomes:** Deliberate-self-harm (DSH)-related hospital admission | 1. Western Australian Data Linkage System: **health** (Midwives Notification System, Hospital Morbidity Data System, Mental Health Information System, Emergency Department Data Collection, Mortality Registrations); **social services** (Western Australia Department for Child Protection and Family Support (CPS) records)<br>2. No<br>3. Yes<br>4. Yes<br>5. 0-20 years | Retrospective, one-time linkage (from a larger 'living' research database using multiple/updated linkages) using probabilistic linkage technique. Linked on demographic information including name, date of birth, residential address, sex, record date, and unique identifiers (e.g., hospital unique medical record number, electoral number). Implied exemption from consent requirements. Data were shared with researchers in de-identified format. Linkage quality assessed through clerical review of a sub-sample of matches; good (>99%) accuracy with ~0.1% missed or invalid links. | N = 351,372 live births in Western Australia from 1986-2000 (N = 21,460 children with an allegation of maltreatment and N = 329,912 children with no allegations). Of children with allegations, N = 11,546 (53.8%) had unsubstantiated allegations only and N = 9914 (46.2%) had 1+ substantiated allegation(s). Children with unsubstantiated allegations only were 51.10% female and 21.05% Aboriginal; those with 1+ substantiated allegation(s) were 55.68% female and 31.02% Aboriginal. | Substantiated and unsubstantiated maltreatment allegations (emotional abuse, physical abuse, sexual abuse, neglect) [Exposure] | 2.3% of children with no maltreatment allegations had a DSH-related hospital admission, compared with 7.47% and 11.22% of children with unsubstantiated allegations only and ≥1 substantiated allegation, respectively. Compared with children without allegations of maltreatment, children with unsubstantiated allegations only and children with at least one substantiated allegation had significantly increased risk of DSH in adolescence (adj. HR = 1.04, 95%CI 1.00, 1.08, $p < 0.01$ and 1.10, 95% CI 1.05, 1.15, $p<0.001$, respectively). For children with ≥1 substantiated allegation (but not those with unsubstantiated allegations only), greater risk of DSH-related admission was significantly associated with higher number of allegations, longer exposure of maltreatment, and more types of maltreatment. |
| Jackisch (2019)<br><br>Sweden | **Purpose:** Descriptive<br><br>**Aims:** To examine the relationship between childhood adversity (ages 0–18) and premature all-cause mortality | 1. Stockholm Birth Cohort Multigenerational Study (SBC Multigen): **health** (cause of death register, delivery records, **social services** (the social register and | Retrospective, one-time linkage using probabilistic linkage technique and supplemental manual searching of local child welfare data (within a system using national | N = 14,004 live births from 1953 living in the greater municipal area of Stockholm in 1963. 49.0% female. N = 1354 (9.7%) had an unsubstantiated child welfare investigation, N = 284 (2.0%) had in-home child welfare | Involvement with child welfare (unsubstantiated investigation, in-home services, out-of-home care placement); stratified by family circumstances | Children with child welfare involvement due to family circumstances were significantly more likely than those who were not to die prematurely (adj. HR for unsubstantiated allegations = 1.53, 95% CI 1.03, 2.26; adj. HR for in-home services = 2.40, 95% CI 1.43, 4.02; adj. HR for out-of-home care placement = 2.07, 95% CI 1.71, 2.51). |

| | | | | | | |
|---|---|---|---|---|---|---|
| | **Outcomes:** Premature all-cause mortality (age 19-65 years) | local child welfare records); **justice** (crime register); **other** (register of population, occupational and income, censuses)<br>2. No<br>3. Yes<br>4. Yes<br>5. 0-65 years (maltreatment occurred from birth to 18) | registries). Data were anonymised. Implied exemption from consent requirements. N = 509 of the 15,117 individuals in the cohort (3.4%) could not be linked. | services, and N = 1266 (9.0%) had an out-of-home care placement. Of all children involved with CWS, N = 1770 were involved due to family circumstances. | (abuse, neglect) and behavioural problems [Exposure] | |
| Jenkins (2012)<br><br>USA | **Purpose:** Descriptive<br><br>**Aims:** To generate descriptive data about engagement among parents whose children have been removed by the child welfare system. To examine the effect of initial level of parental engagement on family reunification. To examine the effect of child welfare system level engagement efforts on family reunification<br><br>**Outcomes:** Court-ordered reunification | 1. **Social services** (CPS records); **study-specific** (Client Engagement in Child Protective Services questionnaire)<br>2. Yes<br>3. No<br>4. No<br>5. 0-5 years | Could not categorise due to insufficient description of linkage techniques. Linked on court number. De-identified. | N = 150 parents: 80.7% mothers; mean age = 27.8 years (SD = 6.7); 55.3% Hispanic, 37.3% White; 5.4% Black; 2% other ethnicity. Children: mean age at detention = 28.0 (SD = 19.8) months; 52.7% female; primary allegation type: 88% general neglect, 12% other. | Removal due to physical abuse, severe neglect, general neglect, other (incl. emotional abuse, sexual abuse, failure to protect, caretaker absence) [Exposure] | Small predictive relationship between early engagement and family reunification within 6 months of removal. No significant relationship between social worker/collateral contacts or any of the parent (age, gender, ethnicity, level of education, risk factors incl. substance use/mental health concerns) or child level (age at removal, gender, ethnicity, primary allegation type) factors. |

| | | | | | | |
|---|---|---|---|---|---|---|
| King (2017)<br><br>USA | **Purpose:** Descriptive<br><br>**Aims:** To examine the relationship between time in foster care and giving birth as an adolescent among a cohort of girls who experienced substantiated maltreatment<br><br>**Outcomes:** Teenage birth (i.e. first birth at age 12-19) | 1. **Health** (vital records); **social services** (California Child Welfare Services/Case Management System (CPS) records)<br>2. No<br>3. Yes<br>4. Yes<br>5. Mothers: 10-19 years | Retrospective, one-time linkage. Linked on a combination of unique and non-unique identifiers. Personally-identifiable data shared through agreements between university and data-holding organisations. Linkage validation/quality assessment ND. | N = 85,766 girls with substantiated allegations of maltreatment after their 10th birthday (27.1% of whom had a foster care placement). 47.7% Latina, 29.4% White, 14.0% Black, 4.3% Asian/Pacific Islander, 0.8% Native American. 21.9% had experienced sexual abuse, 20.2% physical abuse, 2.2% severe neglect, 41.1% severe neglect, 18.0% caretaker absence/incapacity, 16.7% other neglect, and 19.8% emotional abuse (categories not mutually exclusive); 24.0% experienced recurrence of maltreatment. | Substantiated maltreatment including: (1) sexual abuse, (2) physical abuse, (3) severe neglect, (4) general neglect, (5) caretaker absence/incapacity, (6) other neglect, and (7) emotional abuse, with recurrence defined as another substantiated allegation after the initial allegation but before giving birth (for those who gave birth) [Exposure] | 17.8% of girls with substantiated maltreatment after age 10 subsequently gave birth before age 20. 19.5% of those who had spent time in foster care gave birth vs. 17.2% of those who had not (adj. HR = 1.10, 95% CI 1.06, 1.14). Risk factors most associated with a higher chance of first birth were older age at substantiated allegation and Latina, Black, and Native American ethnicity (vs. White; Asian/Pacific Islander girls had a significantly lower chance of childbirth compared with White girls). Girls with recurrent maltreatment and those with substantiated reports of sexual or physical abuse, general neglect, or caretaker absence/incapacity also had a higher risk of giving birth, but with small effect sizes (girls who had experienced emotional abuse had lower rates of birth; severe/other neglect had no significant association). |
| King (2014)<br><br>USA | **Purpose:** Descriptive<br><br>**Aims:** To estimate the annual incidence of births among girls who were placed in foster care<br><br>**Outcomes:** Birth rates for 15-17-year-old girls | 1. **Health** (birth records); **social services** (California Child Welfare Services/Case Management System (CPS) records)<br>2. No<br>3. Yes<br>4. Yes<br>5. ND-17 years (must have been in foster care at time of birth, but no placement start date requirements listed) | Retrospective, one-time linkage using probabilistic linkage technique. Linked on a combination of unique (i.e. social security number) and non-unique (e.g. first name, date of birth) identifiers. Data shared via agreement between university and data-holding organisations. Linkage validation/quality assessment ND. | All girls in the California foster system who gave birth aged 15-17 between 2006 and 2010 (N = 62,402). | Sexual abuse, physical abuse, or neglect resulting in foster care placement [Exposure] | Birth rates were marginally higher for girls in foster care versus the general California population (average birth rates = 3.2 per 100 and 2.0 per 100, respectively, over the study period). Birth rates were not significantly associated with the removal reason (i.e. sexual/physical abuse or neglect); average birth rates for sexual and physical abuse were both 2.8 per 100 and for neglect was 3.4 per 100. |

| | | | | | | |
|---|---|---|---|---|---|---|
| Kisely (2018)<br><br>Australia | **Purpose:** Descriptive<br><br>**Aims:** To examine whether substantiated child maltreatment is associated with adverse psychological outcomes in early adulthood<br><br>**Outcomes:** Adverse psychological outcomes in early adulthood | 1. **Social services** (Department of Families, Youth and Community Care (CPS) records); **study-specific** (Mater Hospital-University of Queensland Study of Pregnancy measures incl. Youth Self-Report, Centre for Epidemiological Studies - Depression scales, World Health Organization Composite International Diagnostic Interview)<br>2. Yes<br>3. Yes<br>4. Yes<br>5. 0-21 years (maltreatment measured from 0-14 years) | Retrospective, one-time linkage using probabilistic linkage technique (from larger, near real-time linked research database). Linked on an identification number. Data provided to researchers in anonymised format. High match rate: could not match approx. 0.1% of records. | N = 3778 young people from the Mater Hospital-University of Queensland Study of Pregnancy (52.4% of original sample). 52.6% female; 89.8% White, 3.8% Indigenous. 4.5% had substantiated maltreatment reports (N.B. loss to follow-up was more common for those with substantiated reports.) 2.4% had a substantiated report of emotional abuse, 2.1% of physical abuse, 1.9% of neglect, and 1.4% of sexual abuse; 2.3% had multiple types of substantiated maltreatment. | Substantiated child maltreatment reports (including physical abuse, sexual abuse, emotional abuse, and neglect) before age 14 [Exposure] | Depressive symptoms on the Centre for Epidemiological Studies-Depression (CES-D) scale and internalising and externalising behaviours on the Youth Self-Report were strongly associated with substantiated notifications in the adjusted models for all types of maltreatment except sexual abuse. For those who completed the Composite International Diagnostic Interview-Auto, substantiated maltreatment reports were significantly associated with anxiety disorders but showed an inconsistent relationship for depressive disorders. |

| | | | | | | |
|---|---|---|---|---|---|---|
| Kisely (2020a)<br><br>Australia | **Purpose:** Descriptive<br><br>**Aims:** To examine whether notified and/or substantiated child maltreatment is associated with the prevalence and persistence of smoking in early adulthood<br><br>**Outcomes:** Any smoking versus no smoking in the previous week; any cigarette use; persistent smoking | 1. **Social services** (Department of Families, Youth and Community Care (CPS) records); **study-specific** (Mater Hospital-University of Queensland Study of Pregnancy measures incl. smoking)<br>2. Yes<br>3. Yes<br>4. Yes<br>5. 0-21 years (maltreatment measured from 0-14years) | Retrospective, one-time linkage using probabilistic linkage technique (from larger, near real-time linked research database). Linked on an identification number. Data provided to researchers in anonymised format. High match rate: could not match approx. 0.1% of records. | N = 3758 young people from the Mater Hospital-University of Queensland Study of Pregnancy (52.1% of original sample). 52.6% female; 89.8% White, 3.8% Indigenous. 7.5% had any history of notified maltreatment (N.B. loss to follow-up was much more common for those with maltreatment notifications.) 4.2% had notifications for physical abuse, 4.0% for emotional abuse, 3.9% for neglect, and 2.5% for sexual abuse; 3.6% had ≥2 maltreatment notifications. 4.4% had substantiated maltreatment reports, of which the most common type of maltreatment was emotional abuse or neglect. 1.7% had ≥2 substantiated reports. | Notifications and substantiations of alleged maltreatment (including physical abuse, sexual abuse, emotional abuse, and neglect) before age 14 [Exposure] | 35.3% of the cohort were smokers at the 21-year follow-up, of whom 21.2% were 'persistent' smokers (i.e. had also reported smoking at 14-year follow-up). Those who had notifications of maltreatment were significantly more likely than those who did not to be smokers at 21 (adj. OR = 1.80, 95% CI 1.38, 2.34) and persistent smokers (adj. OR = 1.80, 95% CI 1.24, 2.62). The same was true for those with substantiated maltreatment notifications (adj. ORs = 1.88, 95% CI 1.34, 2.63 for smoker at 21 and 1.84, 95% CI 1.16, 2.90 for persistent smoker). Estimates were similar for any cigarette use. All forms of maltreatment except sexual abuse were associated with 12-month nicotine use disorder (adj. ORs between 1.45 and 2.13). |

| | | | | | | |
|---|---|---|---|---|---|---|
| Kisely (2020b)<br><br>Australia | **Purpose:** Descriptive<br><br>**Aims:** To examine the association of different types of child maltreatment with alcohol use disorders at 21 years of age<br><br>**Outcomes:** Heavy alcohol use at 21 years; severe impact of alcohol use; lifetime diagnoses of alcohol use disorders | 1. **Social services** (Department of Families, Youth and Community Care (CPS) records); **study-specific** (Mater Hospital-University of Queensland Study of Pregnancy measures incl. alcohol use)<br>2. Yes<br>3. Yes<br>4.Yes<br>5. 0-21 years (maltreatment measured from 0-14 years) | Retrospective, one-time linkage using probabilistic linkage technique (from larger research database using repeated one-time/near real-time linkage). Linked on an identification number. Data provided to researchers in anonymised format. High match rate: could not match approx. 0.1% of records. | N = 3762 young people from the Mater Hospital-University of Queensland Study of Pregnancy (52.1% of original sample). 52.6% female; 89.8% White, 3.8% Indigenous. 7.5% had any history of notified maltreatment. 4.5% had substantiated maltreatment reports (2.4% for emotional abuse, 2.1% for physical abuse, 1.2% for neglect, and 1.4% for sexual abuse; N.B. loss to follow-up was greater for those with substantiated maltreatment reports). | Substantiated child maltreatment reports (including physical abuse, sexual abuse, emotional abuse, and neglect) before age 14 [Exposure] | 10.8% of the cohort reported heavy alcohol use at the 21-year follow-up. Those with substantiated reports of emotional abuse (but not other types of maltreatment) had a significantly increased likelihood of heavy alcohol use in the last month (adj. OR = 1.856, 95% CI 1.038, 3.319) compared with those who did not have any substantiated reports of maltreatment. Those with substantiated reports of neglect (but not other types of maltreatment) had a significantly increased likelihood of lifetime alcohol use disorder (adj. OR = 1.947, 95% CI 1.047, 3.621) compared with those who did not have any substantiated reports of maltreatment. No type of maltreatment was significantly related to severe impact of alcohol use in the last month. |
| Kjelsberg (1999a)<br><br>Norway | **Purpose:** Descriptive<br><br>**Aims:** To identify in former adolescent psychiatric inpatients factors predicting a non-negative outcome (defined as not having entered the registers of delinquency, disability, and death during a 15-year follow-up period)<br><br>**Outcomes:** Non-negative outcome: lack of delinquency, disability, death | 1. **Health** (psychiatric inpatient records, Death Cause Registry); **justice** (National Crime Registry (all offences)); **social services** (Disability Benefits Registry)<br>2. No<br>3. No<br>4. No<br>5. 11-22 years during inpatient stay; followed up 15-33 years after hospitalisation | Near real-time linkage with deterministically-linked registry data. Linked using unique personal identifiers. Implied exemption from consent requirements. Linkage validation/quality assessment ND. | N = 932 adolescent psychiatric inpatients followed up over 15-33 years. 49.2% female; mean age at first admission = 14.9 years (SD 1.5) and 39.5 years (SD 5.6) at follow-up. 19.7% had experienced physical abuse at home. | Physical abuse (no further detail given) [Exposure] | Physical abuse at home was significantly associated with having a negative outcome ($p<0.001$): 21.9% of those with a history of physical abuse had no negative outcome vs. 38.5% of those without such history. |

| | | | | | | |
|---|---|---|---|---|---|---|
| Kjelsberg (1999b)<br><br>Norway | **Purpose:** Descriptive<br><br>**Aims:** To identify predictors of later delinquency in adolescent psychiatric inpatients<br><br>**Outcomes:** Delinquency, defined as having a record in the National Crime Registry (crime or misdemeanour) | 1. **Health** (psychiatric inpatient records, Death Cause Registry); **justice** (National Crime Registry (all offences)); **social services** (Disability Benefits Registry)<br>2. No<br>3. No<br>4. No<br>5. 11-22 years during inpatient stay; followed up 15-33 years after hospitalisation | Near real-time linkage with deterministically-linked registry data. Linked using unique personal identifiers. Implied exemption from consent requirements. Linkage validation/quality assessment ND. | N = 932 adolescent psychiatric inpatients followed up over 15-33 years. 49.2% female; mean age at first admission = 14.9 (SD 1.5) and 39.5 (SD 5.6) at follow-up. | Verbal abuse (no further detail given) [Exposure] | Male patients who had experienced verbal abuse were 1.5 times as likely as those who had not to have a record of delinquency (adj. RR = 1.5, 95% CI 1.1 - 2.0). Female patients who had experienced verbal abuse were also 1.5 times as likely as those who had not to have a record of delinquency (adj. RR = 1.5, 95% CI 1.0 - 2.1). Of environmental factors studied, abuse was the strongest independent predictor of delinquency. |
| Kjelsberg (1999c)<br><br>Norway | **Purpose:** Descriptive<br><br>**Aims:** To identify predictors of early death in former adolescent psychiatric inpatients<br><br>**Outcomes:** early death | 1. **Health** (psychiatric inpatient records, Death Cause Registry); **justice** (National Crime Registry (all offences)); **social services** (Disability Benefits Registry)<br>2. No<br>3. No<br>4. No<br>5. 11-22 years during inpatient stay; followed up 15-33 years after hospitalisation | Near real-time linkage with deterministically-linked registry data. Linked using unique personal identifiers. Implied exemption from consent requirements. Linkage validation/quality assessment ND. | N = 1095 adolescent psychiatric inpatients followed up over 15-33 years. 49.2% female; mean age at first admission = 15.0 years (SD 1.6) and 39.5 years (SD 5.5) at follow-up. | Physical or verbal abuse (no further detail given) [Exposure] | Physical or verbal abuse at home was not significantly associated with early death (no further details given). |

| | | | | | | |
|---|---|---|---|---|---|---|
| Lanier (2017)<br><br>USA | **Purpose:** Descriptive<br><br>**Aims:** To use large, longitudinal, multisector administrative datasets of families investigated by child welfare services for maltreatment to explore which children are more likely to later enter psychiatric residential treatment facilities<br><br>**Outcomes:** Entrance into psychiatric residential treatment facilities | 1. **Health** (Medicaid); **social services** (CPS records, Temporary Assistance for Needy Families (income maintenance))<br>2. No<br>3. Yes<br>4. Yes<br>5. 5-17 years | Multiple one-time linkages. Linkage technique (probabilistic vs. deterministic) ND. Linked on common identifiers, names, birthdates, Social Security Numbers. Implied exemption from consent requirements. Linkage validation/quality assessment ND. | N = 183,795 children whose families were investigated for maltreatment. 50.7% female; 50.2% White. 17.7% had a substantiated first investigation (mean age at investigation = 8.9 years (SD 4.0)) | Child maltreatment investigations and substantiations [Exposure] | N = 720 children (0.43%) entered into a psychiatric residential treatment facility. In the final model (controlling for interaction effects between income assistance and child welfare systems), children who had a foster care placement were more likely than those who did not to enter a treatment facility (adj. HR = 11.63, p<0.001), as were children whose first investigation was substantiated (adj. HR = 1.58, p<0.001), boys (adj. HR = 1.52, p<0.001), White children (adj. HR vs. non-White = 1.47, p<0.001), and older children (adj. HR = 1.08, p<0.001). Receipt of income maintenance was not significantly associated with entry into a psychiatric residential treatment facility. |
| Lanier (2010)<br><br>USA | **Purpose:** Descriptive<br><br>**Aims:** To examine if maltreatment predicted increased risk of hospital-based treatment prior to age 18 years for asthma, cardio-respiratory, and non-sexually transmitted infectious disease in a sample of low-income children<br><br>**Outcomes:** Asthma, cardio-respiratory, and non-sexually transmitted infectious disease | 1. **Health** (vital statistics, parent Medicaid mental health records, Medicaid reimbursed child healthcare, emergency department records, children's mental health and disability records from special education and Department of Mental Health); **social services** (child abuse and neglect reports, child welfare service records, Aid to Families with Dependent Children (income maintenance)) | Multiple one-time linkages. Linkage technique (probabilistic vs. deterministic) ND. Linked on common identifier/ combination of individual identifiers. Linkage validation/quality assessment ND. | N = 6282 low-income children (no further characteristics provided for the full group) | Investigated child maltreatment reports (regardless of substantiation) [Exposure] | Children with maltreatment reports had a significantly higher risk of hospital treatment than those without reports (adj. HRs = 1.73, 95% CI 1.47, 2.04 for asthma; 2.07, 95% CI 1.87, 2.29 for cardio-respiratory; 2.09, 95% CI 1.85, 2.36 for non-sexually transmitted infections). Recurrent reports were associated with a greater number of hospital care episodes (1.05 additional episodes per additional maltreatment report, p<0.0001). Maltreatment type (abuse vs. neglect) and substantiation of first report were not significantly associated with hospital treatment. |

| | | | | | | |
|---|---|---|---|---|---|---|
| | | 2. No<br>3. Yes<br>4. Yes<br>5. 0-18 years | | | | |
| Laurens (2020)<br><br>Australia | **Purpose:** Descriptive<br><br>**Aims:** To examine associations between child protection involvement and 3rd- and 5th-grade reading and numeracy attainment, while controlling multiple other adversities<br><br>**Outcomes:** Reading and numeracy attainment at the 3rd- and 5th-grades | 1. New South Wales - Child Development Study (NSW-CDS): **education** (NSW Education Standards Authority National Assessment Program – Literacy and Numeracy, Australian Government Department of Education Australian Early Development Census (AEDC)); **health** (NSW Registry of Births, Deaths and Marriages, NSW Ministry of Health Perinatal Data Collection, Mental Health Ambulatory records, Emergency Department Data Collection, Admitted Patients Data Collection); **justice** (NSW Bureau of Crime Statistics and Research Reoffending data); **social services** (Department of Family & Community Services Child Protection Case Management System)<br>2. No | Multiple one-time linkages using probabilistic linkage technique. Linked on name, date of birth, residential addresses, and sex. Exempt from consent requirements. Data provided to researchers in de-identified format. Low rates of false-positive linkages for children's data (0.3%) and for linkage of parents to children (0.5%). | N = 56,860 children enrolled in the New South Wales - Child Development Study. 49.8% female; 6.4% Indigenous. 9.4% of children had a child protection report 3rd grade and 15.5% by 5th grade; 1.6% had a substantiated report by 3rd grade and 2.1% by 5th grade. | Child protection reports, coded hierarchically by most severe presentation: (1) children with an out-of-home care placement, (2) substantiated 'risk of significant harm' reports (risk/actual harm) without out-of-home care placement, (3) unsubstantiated but investigated reports, (4) un-investigated ('screened out') reports [Exposure] | Compared with children with no child protection report, children with unsubstantiated (adj. OR = 1.32, 95% CI 1.19, 1.47) and substantiated reports (adj. OR = 1.50, 95% CI 1.26, 1.78) had a significantly higher risk of below average reading scores in 3rd grade, and those with all categories of report had a significantly higher risk of below average reading scores at 5th grade (adj. ORs = 1.30 to 1.64). Children with all categories of report had a significantly higher risk than those without a report of below average numeracy scores in 3rd and 5th grade (adj. ORs = 1.24 to 1.58). Children with screened out, unsubstantiated, and substantiated reports had a lower chance than those without reports of scoring above average on reading in 3rd and 5th grade (adj. ORs = 0.75-0.76), but there was no relationship for those with out-of-home care placements. Children with unsubstantiated and substantiated reports had a lower chance than those without reports of scoring above average on numeracy in 3rd grade and 5th grade (adj. ORs = 0.61 to 0.77) and those with screened out reports had a lower chance in 5th grade only. |

| | | | | | | |
|---|---|---|---|---|---|---|
| | | 3. Yes<br>4. Yes<br>5. 0 years-5th grade (approx. 10 years) | | | | |
| Lee (2009)<br><br>USA | **Purpose:** Descriptive<br><br>**Aims:** To examine the needs and outcomes of children in special education according to child welfare involvement<br><br>**Outcomes:** Emergency room treatment for mental health, school problems (incl. school behaviours, truancy, drop-out, withdraw due to behaviour), juvenile delinquency | 1. **Education** (special education case files, truancy records); **health** (mental health (emergency room and Department of Mental Health records)); **justice** (juvenile court); **social services** (CPS records); **other aggregate data** (census-tract records for income/parental education)<br>2. No<br>3. No<br>4. Yes<br>5. 0-ND years | Retrospective, one-time linkage using probabilistic linkage technique. Linked on common identification numbers and other identifiers. Identifiable data. All matches were cross-checked for consistency across data sets; case files were checked against administrative records. | N = 371 youth with special education files. 35.5% female; 84.3% Black; mean age at first assessment for special education = 7.4 years. 53% had a report of child abuse or neglect at the start of the study period; by the end of the study, 43% of children without reports at baseline had reports of abuse or neglect. | Child welfare case files (incl. cases of abuse/neglect that were substantiated or had multiple report cases; i.e. those with 1 unsubstantiated case were not included) [Exposure] | Children with experience of abuse/neglect without services (CAN), children who received child welfare services but had no foster care placements (CWS), and children with at least one foster care placement (FC) were all significantly more likely to be in special education for an emotional disturbance (ED) than those with experience of poverty only (non-CAN) ($p<0.0001$). In the CAN, CWS, and FC groups, ED special education status did not significantly differ by gender, parental age at birth, parental education. With minor exceptions, those in the CAN, CWS, and FC groups generally had lower rates of recorded needs/problems than the non-CAN group. Children in the CAN, CWS, and FC groups were no more likely than children in the non-CAN to have a delinquency petition. Children in CWS (HR = 3.20, $p=0.038$) and FC (HR = 4.96, $p=0.003$) groups were significantly more likely than those in the non-CAN to have an emergency room mental health treatment episode, but this was not true for the CAN group. Children in the CAN (HR = 5.64, $p=0.036$), CWS (HR = 11.64, $p=0.0006$), and FC (HR = 7.32, $p=0.009$) groups were all more likely than those in the non-CAN group to have school problems. For children with an ED diagnosis, those with child welfare contact were more likely to have a negative outcome than those without. |

| | | | | | | |
|---|---|---|---|---|---|---|
| Leslie (2000)<br><br>USA | **Purpose:** Descriptive<br><br>**Aims:** To determine factors influencing outpatient mental health service use by children in foster care<br><br>**Outcomes:** Outpatient mental health service use (number of visits) | 1. **Health** (San Diego County Mental Health Management Information System, Medicaid Management Information System); **social services** (Social Service Reporting System (CPS records)); **study-specific** (cohort data incl. measures of mental health)<br>2. Yes<br>3. No<br>4. No<br>5. 0-16 years | Multiple one-time linkages. Linkage technique (probabilistic vs. deterministic) ND. Linked on children's Social Security Numbers, Department of Social Services case numbers, name, birth date, and county identifier number. Linkage validation/quality assessment ND. | N = 480 children in foster care. 55.2% female; 44.2% Caucasian, 27.7% African American, 22.5% Latino, 5.6% Asian/other, 44.2% Caucasian. 14.0% had experienced sexual abuse, 25.4% physical abuse, 68.1% neglect, 13.3% emotional abuse, and 46.2% caretaker absence. | Substantiated reports of maltreatment (incl. sexual abuse, physical abuse, emotional abuse, caregiver absence, and neglect) resulting in home removal [Exposure] | Mean number of outpatient mental health visits per year were 2.79, 4.15, 2.84, 3.20, and 2.42 for sexual abuse, physical abuse, neglect, emotional abuse, and caretaker absence, respectively. Removal due to sexual abuse, physical abuse, neglect, and emotional abuse were not significantly associated with the number of outpatient mental health visits in the adjusted model; removal due to caretaker absence was associated with fewer visits (rate ratio: 0.64, 95% CI: 0.48, 0.86; $p<0.003$). |
| Lewis (2011)<br><br>USA | **Purpose:** Descriptive<br><br>**Aims:** To examine the association between childhood maltreatment and adolescent smoking and the extent to which internalising behavioural problems mediate this hypothesised link<br><br>**Outcomes:** Adolescent smoking | 1. **Social services** (CPS records); **study-specific** (LONGSCAN study, including data on maltreatment, mental health, and smoking)<br>2. Yes<br>3. No<br>4. No<br>5. 0-16 years (maltreatment measured 0-12 years) | Could not categorise due to insufficient description of linkage techniques. Consent from parents and assent from young people. Linkage validation/quality assessment ND. | N = 522 children from the Longitudinal Studies of Child Abuse and Neglect (LONGSCAN) study. 51% female; 53% African American, 28% White, 12% mixed race, 7% other race/ethnicity. 81% had experienced ≥1 form of maltreatment. | CPS reports (physical abuse, sexual abuse, emotional abuse, or neglect) and/or self-reported physical abuse, sexual abuse, or emotional abuse [Exposure] | Compared with their peers, a higher proportion of youth with experience of maltreatment smoked at age 16 (19% vs. 17%). Childhood maltreatment predicted smoking at age 16 (adj. OR = 1.96, $p<0.05$). Maltreatment before age 12 was associated with internalising problems at age 14, which were associated with smoking at 16. Internalising problems partially mediated the relationship between maltreatment and smoking. |

| | | | | | | |
|---|---|---|---|---|---|---|
| Maclean (2016)<br><br>Australia | **Purpose:** Descriptive<br><br>**Aims:** To examine prevalence, risk, and protective factors for low educational achievement among children involved with the child protection system compared to other children<br><br>**Outcomes:** Low reading achievement in Year 3 | 1. Western Australian Data Linkage System: **health** (Western Australian Register of Developmental Anomalies, Hospital Morbidity Data System, Intellectual Disability Exploring Answers (IDEA), Mental Health Information System, Midwives Notification System, Birth Registrations); **education** (Western Australian Department of Education records); **social services** (Department of Child Protection and Family Support (CPS) records)<br>2. No<br>3. Yes<br>4. Yes<br>5. 0-Year 3 (approx. 8 years) | Near real-time ('living') linkage from multiple updates using probabilistic linkage technique. Linked on medical record number, first and last name, initial, date of birth, sex, and addresses. Implied exemption from consent requirements. Data were shared with researchers in de-identified format. Linkage quality assessed through clerical review of a sub-sample of matches; very low (~0.1%) false positive and false negative matches. | N = 46,838 children born in Western Australia who were eligible to sit the Year 3 National Assessment of Literacy and Numeracy (NAPLAN) reading test between 2008-2010. 48.8% female; mean age at NAPLAN exam = 8 years, 5 months; 10.4% Aboriginal. 5.8% had a maltreatment allegation (2.1% physical abuse, 1.9% sexual abuse, 1.2% emotional abuse, 2.5% neglect) and 2.9% had a substantiated allegation. | Allegations and substantiations of maltreatment (physical abuse, sexual abuse, emotional abuse, and neglect) [Exposure] | Prevalence of low reading achievement was higher for children with maltreatment allegations than for those without (30.2% vs. 11.4%; adj. OR = 1.46, 95% CI 1.31, 1.63), with overrepresentation of children with allegations within the bottom decile. All types of maltreatment except emotional abuse were associated with a significantly higher risk of low achievement (adj. ORs = 1.53, 95% CI 1.29, 1.82 for sexual abuse; 1.52, 95% CI 1.30, 1.77 for neglect; 1.26, 95% CI 1.07, 1.49 for physical abuse). Children with unsubstantiated allegations (adj. OR = 1.49, 95% CI 1.29, 1.72), those with substantiated allegations who remained at home (adj. OR = 1.63, 95% CI 1.33, 2.00), and those with out-of-home placements (adj. OR = 1.28, 95% CI 1.05 2.55) all had significantly higher risk of low achievement compared to peers with no involvement when taking attendance into account. |
| Maclean (2017b)<br><br>Australia | **Purpose:** Descriptive<br><br>**Aims:** To examine the influence of placement stability, reunification, type of care, time in care and age at entry to care on children's educational outcomes<br><br>**Outcomes:** Low reading achievement in Year 3 | 1. Western Australian Data Linkage System: **health** (Western Australian Register of Developmental Anomalies, Hospital Morbidity Data System, Intellectual Disability Exploring Answers (IDEA), Mental Health Information System, Midwives | Near real-time ('living') linkage from multiple updates using probabilistic linkage technique. Linked on medical record number, first and last name, initial, date of birth, sex, and addresses). Implied exemption from consent requirements. Data | N = 235,045 children born in Western Australia children born in Western Australia who were eligible to sit the Year 3 National Assessment of Literacy and Numeracy (NAPLAN) reading test between 2008-2010 (N = 2160 children with substantiated maltreatment and subsequent out-of-home | Out-of-home care placement with substantiated maltreatment [Exposure] | 30.5% children who had been reunified at the time of the test and 29.1% of children in care at the time of the test had low reading achievement scores on the NAPLAN, compared with 9.4% of children with no CPS contact. Associations between number of placement changes and scores did not follow a dose-response relationship (only 2-3 placements were associated with a significantly higher likelihood of low achievement compared with 0 placements; adj. OR = 1.41, 95% CI 1.18, |

| | | | | | | |
|---|---|---|---|---|---|---|
| | | Notification System, Birth Registrations); **education** (Western Australian Department of Education records); **social services** (Department of Child Protection and Family Support (CPS) records)<br>2. No<br>3. Yes<br>4. Yes<br>5. 0-Year 3 (approx. 8 years) | were shared with researchers in de-identified format. Linkage quality assessed through clerical review of a sub-sample of matches; very low (~0.1%) false positive and false negative matches. | care placement and N = 232,885 children with no maltreatment allegations). 48.7% female; 6.6% Aboriginal. | | 1.68). Children with a short duration in current placement (≤12 months) had a significantly higher likelihood of low achievement compared with children with no CPS contact (adj. OR = 1.44, 95% CI 1.12, 1.85) but this was not true for placements >12 months. Children reunified at the time of the test did significantly worse than those without CPS contact (adj. OR = 1.35, 95% CI 1.16, 1.57) but there was no significant difference for children in care at the time of the test. Children reunified between 1 and 6 years before the exam had a significantly lower chance of low achievement than those reunified ≤12 months of the exam, but there was no relationship for those reunified >6 years before the exam. Scores varied by primary type of care, with lower proportions for those in foster care (23.9%) and higher for those in residential care (41.6%). Children with a primary placement kinship care, foster care, or residential care did not have a significantly higher chance than control children of low achievement (those in mixed/no primary type of care did; adj. OR = 1.44, 95% CI 1.13, 1.84). Those with a most recent placement in residential care (but not kinship or foster care) had a higher chance than control children of low achievement (adj. OR = 1.50, 95% CI 1.08, 2.08). Effects were slightly attenuated when attendance was taken into account. |

| | | | | | | |
|---|---|---|---|---|---|---|
| Maclean (2018)<br><br>Australia | **Purpose:** Descriptive<br><br>**Aims:** To estimate the influence of out-of-home care on reading scores, attendance, and suspensions by comparing a matched sample of maltreated children who entered out-of-home care and maltreated children who remained at home<br><br>**Outcomes:** Reading achievement in Year 9 | 1. Western Australian Data Linkage System: **health** (Western Australian Register of Developmental Anomalies, Hospital Morbidity Data System, Intellectual Disability Exploring Answers (IDEA), Mental Health Information System); **education** (Western Australian Department of Education records); **social services** (Department of Child Protection and Family Support (CPS) records)<br>2. No<br>3. Yes<br>4. Yes<br>5. 0-Year 9 (approx. 14 years) | Near real-time ('living') linkage from multiple updates using probabilistic linkage technique. Linked on medical record number, first and last name, initial, date of birth, sex, and addresses). Implied exemption from consent requirements. Data were shared with researchers in de-identified format. Linkage quality assessed through clerical review of a sub-sample of matches; very low (~0.1%) false positive and false negative matches. | N = 3297 children born in Western Australia children born in Western Australia who were eligible to sit the Year 9 Western Australian Literacy and Numeracy Assessment or National Assessment of Literacy and Numeracy (NAPLAN) reading test between 2005-2013 who had substantiated reports of maltreatment before Year 9 (N = 1166 of whom had ever been placed in out-of-home care). In-home care only: 58.5% female; 15.4% Aboriginal; 19.5% had an allegation of neglect, 28.8% of sexual abuse, 27.0% of physical abuse, and 9.9% of emotional abuse. Out-of-home care placement: 52.5% female; 28.7% Aboriginal; 54.3% had an allegation of neglect, 23.8% of sexual abuse, 44.3% of physical abuse, and 23.2% of emotional abuse. | Substantiated maltreatment (physical abuse, sexual abuse, emotional abuse, neglect), with or without out-of-home care placement [Exposure] | 26% of children in in-home care and 34% of children with an out-of-home care placement had low reading scores (bottom decile). After adjustment for differences in risk factors at baseline, there was no significant difference between children who did and did not have out-of-home care placements in terms of reading achievement or suspensions but children with out-of-home care placements were less likely to have high school absence (≥20% of days) than those with in-home care (adj. OR = 0.37, 95% CI 0.15, 0.91, p=0.03). |

| | | | | | | |
|---|---|---|---|---|---|---|
| Matheson (2017)<br><br>Australia | **Purpose:** Descriptive<br><br>**Aims:** To determine the independent and moderating effects of maltreatment and parental schizophrenia spectrum disorder on early childhood social-emotional functioning<br><br>**Outcomes:** Social-emotional functioning (as measured by the AEDC) | 1. New South Wales - Child Development Study (NSW-CDS): **education** (Australian Government Department of Education Australian Early Development Census (AEDC)); **health** (NSW Registry of Births, Deaths and Marriages — Birth Registrations, Admitted Patients Data Collection, Mental Health Ambulatory Data Collection); **social services** (NSW Family and Community Services Child Protection Case Management System – Key Information Directory System (CPS) records)<br>2. No<br>3. Yes<br>4. Yes<br>5. 0-5 years | Dataset from a larger database of repeated one-time/near real-time linkage using probabilistic linkage technique. Linked on name, date of birth, residential addresses, and sex. Data were provided to researchers in de-identified format. Low rates of false-positive linkages for children's data (0.3%) and for linkage of parents to children (0.5%). | N = 69,116 kindergarten-aged children enrolled in the New South Wales Child Development Study. 49.5% female; mean age = 5.6 years (SD 0.4). 2.9% had a CPS report (0.6% for physical abuse, 1.6% for emotional abuse, 0.3% for sexual abuse, 1.0% for neglect); 0.6% had ≥2 types of maltreatment reports. | Substantiated CPS reports for maltreatment (physical maltreatment, emotional maltreatment, sexual maltreatment, or neglect) [Exposure] | Controlling for parental schizophrenia spectrum disorder and other potential confounders, children with experience of maltreatment had significantly a higher likelihood than those without of vulnerability across all five AEDC domains (poor social competency, poor prosocial/helping behaviour, anxious/fearful behaviour, aggressive behaviour, hyperactivity/inattention; adj. ORs 1.7-3.2). When examined separately, all types of maltreatment showed medium-sized effects for poor social competency, aggressive behaviour, and hyperactive-inattentive behaviour and small-to-medium-sized effects for prosocial/helping behaviour and anxious/fearful behaviour. Dose-response relationships for 1 vs. ≥2 types of maltreatment were apparent for poor social competency, anxious/fearful behaviour, aggressive behaviour, and hyperactive-inattentive behaviour (and to a lesser extent for prosocial/helping behaviour). Effects of maltreatment on social-emotional functioning were larger for children whose parents did not have a history of schizophrenia spectrum disorder. |
| Meuleners (2010)<br><br>Australia | **Purpose:** Descriptive<br><br>**Aims:** To investigate the incidence and nature of interpersonal violence hospitalisations for victims aged 11–18 years and to identify subgroups at risk of repeat hospital admissions | 1. Western Australian Data Linkage System: **health** (Hospital Morbidity Data System, Mental Health Information System)<br>2. No<br>3. Yes<br>4. No<br>5. 11-18 years | Near real-time ('living') linkage from multiple updates using probabilistic linkage technique. Linked on medical record number, first and last name, initial, date of birth, sex, and addresses. Data were provided to researchers in de- | All hospitalisations in Western Australia due to interpersonal violence for adolescents 11-18 years old (N = 3607). 75% male; mean age = 16 years (SD 0.3); 32% Indigenous. 4.4% were hospitalised due to 'maltreatment or rape' | US Centres for Disease Control and Prevention code for assault by 'maltreatment or rape' [Exposure] | N = 158 (4.4%) of hospitalisations were for assault by maltreatment or rape. Those hospitalised for maltreatment/rape were not more likely than those hospitalised for bodily force to experience a repeat admission. |

| | | | | | | |
|---|---|---|---|---|---|---|
| | **Outcomes:** Interpersonal violence hospitalisations and repeat admissions | | identified format. Linkage quality assessed through clerical review of a sub-sample of matches; very low (~0.1%) false positive and false negative matches. | | | |
| Morgan (2019)<br><br>Australia | **Purpose:** Descriptive<br><br>**Aims:** To estimate independent and combined effects of familial liability for schizophrenia and exposure to obstetric complications on risk for developing psychotic illness, covarying with exposure to other environmental stressors<br><br>**Outcomes:** Psychotic illness | 1. Western Australia Data Linkage System: **health** (Midwives Notification System, Hospital Morbidity Data Collection, Mental Health Information System, birth registration records); **social services** (Department of Communities, Child Protection and Family Support Division (CPS) databases)<br>2. No<br>3. Yes<br>4. Yes<br>5. 0-31 years | Near real-time ('living') linkage from multiple updates using probabilistic linkage technique. Data were provided to researchers in de-identified format. Linkage quality assessed through clerical review of a sub-sample of matches; very low (~0.1%) false positive and false negative matches. | N = 299,416 children born in Western Australia in 1980-1995 (N = 1046 born to 654 mothers with a lifetime history of schizophrenia and N = 298, 370 born to a mother with no known psychiatric history). | Substantiated maltreatment allegations (sexual abuse, physical abuse, emotional abuse) [Exposure (covariate)] | 9.4% of children of mothers with schizophrenia and 1.6% of children with no known maternal psychiatric history developed a psychotic illness. Of all non-focal adverse environmental exposures studied, childhood abuse had the largest effect size (adj. HR =2.8, 95% CI 2.4, 3.2) in terms of developing a psychotic illness. The population attributable fraction for substantiated childhood abuse on psychotic illness outcomes was 3.5 (95% CI 3.3, 3.8). |

| | | | | | | |
|---|---|---|---|---|---|---|
| Needell (2002)<br><br>USA | **Purpose:** Descriptive<br><br>**Aims:** To examine characteristics of youth emancipated from child welfare (ECW) and probation supervised foster care (EPR)<br><br>**Outcomes:** Receipt of mental health services for emancipating youth; births to emancipating females; deaths of youth who emancipated from foster care; receipt of MediCal due to AFDC/TANF, SSI/disability, or medical indigence after emancipation; and receipt of GED, community college enrolment, California Youth Authority involvement, and state prison involvement for emancipating youth. | 1. **Education** (community college enrolment records, Department of Education General Education Development records); **health** (Vital Statistics, Medi-Cal Eligibility Data System, California Department of Mental Health Services); **justice** (California Department of Corrections, California Youth Authority); **social services** (California Children's Services Archive (CPS records))<br>2. No<br>3. Yes<br>4. No<br>5. 0-16+ years | Multiple one-time linkages to different sources. Linkage technique (probabilistic vs. deterministic) ND. Linked on Social Security Number. Linkage validation/quality assessment ND. | N = 12,306 youth who emancipated from foster care 1992-1997 (N = 11,060 (90%) emancipated under the supervision of a child welfare agency (ECW), N = 1,246 (10 percent) probation supervised (EPR). Comparison group of entry into foster care: N = 227,574.<br><br>N = 11,060 ECW: 61.8% female; 29.1% Black, 43.1% White, 22.7% Hispanic, 1.2% Native American, 4.7% Asian; removal reasons were 60.5% neglect, 15.7% physical abuse, 15.0% sexual abuse, and 8.9% other.<br><br>N = 1246 EPR: 25.0% female; 18.4% Black, 55.3% White, 20.7% Hispanic, 1.8% Native American, 3.9% Asian; removal reasons were 3.6% neglect, 1.0% physical abuse, 0.8% sexual abuse, and 94.5% other. | Removal from home due to neglect (general neglect, severe neglect, or caretaker incapacity), physical abuse, sexual abuse, or other reason (exploitation, child's disability or handicap, relinquishment, disrupted adoptive placement, or voluntary placement) [Exposure] | Likelihood of receiving any mental health services before emancipation, becoming pregnant while in care, receiving Aid to Families with Dependent Children/Temporary Aid to Needy Families or Medi-Cal due to medical indigence did not vary by reasons (types of maltreatment). Likelihood of receiving disability-related Medi-Cal following emancipation was higher (OR 1.37, $p<0.05$) in youth removed due to sexual abuse than to neglect (no significant difference between physical abuse and neglect). |

| | | | | | | |
|---|---|---|---|---|---|---|
| O'Donnell (2010a)<br><br>Australia | **Purpose:** Descriptive<br><br>**Aims:** To determine the extent to which children with a hospital admission related to assault or maltreatment or to a notified sexually transmitted infection have contact with the Western Australian Department for Child Protection. To investigate injuries and conditions often associated with child maltreatment and subsequent contact with the Department for Child Protection<br><br>**Outcomes:** Notifications and substantiations of child maltreatment, hospital admissions related to assault/maltreatment resulting in notifications/substantiations/out-of-home care | 1. Western Australian Data Linkage System: **health** (Midwives Notifications System, birth registrations, Hospital Morbidity Data Collection, Notifiable and Infectious Disease Database); **social services** (Department of Child Protection records)<br>2. No<br>3. Yes<br>4. No<br>5. 0-17 years | Near real-time ('living') linkage from multiple updates using probabilistic linkage technique. Linked on medical record number, first and last name, initial, date of birth, sex, and addresses. Data were provided to researchers in de-identified format. Linkage quality assessed through clerical review of a sub-sample of matches; very low (~0.1%) false positive and false negative matches. | All children aged 0-17 years in Western Australia born between 1990-2005 (N = 397,346). 48.7% female; 5.6% Aboriginal. N = 50,692 notifications of maltreatment (N = 19,207 of these substantiated). | Notifications and substantiations of maltreatment; hospital admissions for child maltreatment and assault (defined by ICD-10 codes) [Outcome] | 4.0% of children with maltreatment notifications and 6.4% of those with substantiations had a maltreatment-related admission. 89.7% of children with maltreatment-related admissions had child protection contact (81.1% had maltreatment notifications, 68.4% had substantiations, and 50.5% were placed in out-of-home care). Child protection contact was highest for the following admissions categories: rib fractures (69%), retinal haemorrhage (67%), sexually transmitted infections <14 years of age (64.3%), malnourishment (38%), and multiple injuries (31%). |

| | | | | | | |
|---|---|---|---|---|---|---|
| O'Donnell (2010b)<br><br>Australia | **Purpose:** Descriptive<br><br>**Aims:** To determine whether children who have child maltreatment allegation or substantiation have a higher rate of general hospital admissions and injury related admissions when compared to other children and to investigate other types of admissions, such as mental health, infections, and admissions due to external causes<br><br>**Outcomes:** Hospital admissions (general, injury-related, mental health, infections, external causes) | 1. Western Australian Data Linkage System: **health** (Midwives Notifications System, birth registrations, death registrations, Hospital Morbidity Data Collection, Mental Health Information System); **social services** (Department of Child Protection records)<br>2. No<br>3. Yes<br>4. No<br>5. 0-15 years | Near real-time ('living') linkage from multiple updates using probabilistic linkage technique. Linked on medical record number, first and last name, initial, date of birth, sex, and addresses. Data were de-identified. Linkage quality assessed through clerical review of a sub-sample of matches; very low (~0.1%) false positive and false negative matches. | All children born in Western Australia between 1990 and 2005. Two study groups were selected, each with a set of cases and associated controls selected from the same population.<br><br>1) N = 13,648 children with a maltreatment allegation. 51.3% female; 20.4% Aboriginal. Comparison group: N = 54,592. 48.3% female; 4.7% Aboriginal<br><br>2) N = 6486 children with a substantiated allegation. 52.1% female; 24.7% Aboriginal. Comparison group: N = 25,944. 48.5% female; 5.0% Aboriginal | Allegations and substantiations of child abuse, neglect, or harm [Outcome] | Children with maltreatment allegations/substantiations had higher mean prior admission rates than controls: 61% of children with a maltreatment allegation/substantiation and 40% of controls had ≥1 hospital admission (mean rates 0.7 and 0.2 per year, respectively). 36% of children with an allegation and 40% of children with a substantiation had ≥2 admissions compared with 18% of controls. Risk of maltreatment allegations (adj. OR = 1.49, 95% CI 1.44, 1.53) and substantiations (adj. OR = 1.74, 95% CI 1.65, 1.83) increased significantly with every one admission per year. All types of admissions were associated with an increased risk of maltreatment allegations and substantiations, with the strongest relationships for mental and behavioural disorders (adj. OR = 10.20, 95% CI 5.14, 20.25 for allegations and 26.54, 95% CI 8.28, 85.07 for substantiations), injuries/poisoning (adj. OR = 9.63, 95% CI 7.85, 11.83 for allegations and 21.47, 95% CI 15.74, 29.29 for substantiations), and external causes (adj. OR = 7.84, 95% CI 6.48, 9.47 for allegations and 17.62, 95% CI 13.21, 23.51 for substantiations). |

| | | | | | | |
|---|---|---|---|---|---|---|
| O'Donnell (2012)<br><br>Australia | **Purpose:** Descriptive<br><br>**Aims:** To determine the proportion of child maltreatment-related emergency department presentations in Western Australia and describe the associated injury types. To investigate the proportion of maltreatment-related emergency department presentations resulting in hospitalisation, the proportion referred to the Department for Child Protection, and their outcomes<br><br>**Outcomes:** Maltreatment-related hospital admissions and associated outcomes (hospitalisations, child protection involvement) | 1. Western Australian Data Linkage System: **health** (Emergency Department Information System, Open Patient Administration System, Health Care and Related Information System, Electronic Patient Administrative System, Hospital Morbidity Data System, Midwives' Notifications and Birth Registrations); **social services** (Department for Child Protection reports)<br>2. No<br>3. Yes<br>4. No<br>5. 0-17 years | Near real-time ('living') linkage from multiple updates using probabilistic linkage technique. Linked on medical record number, first and last name, initial, date of birth, sex, and addresses. Data were de-identified. Linkage quality assessed through clerical review of a sub-sample of matches; very low (~0.1%) false positive and false negative matches. | N = 657,656 children aged 0-17 years in Western Australia with a recorded emergency department admission 2001-2005. For the 0.03% of presentations identified as maltreatment-related: 55.9% female; 24.3% Aboriginal. | Maltreatment-related emergency department presentations (based on ICD-10 codes); allegations and substantiations of child maltreatment [Outcome] | 0.03% of all emergency department presentations were identified as maltreatment-related. 22% of these had a hospital admission and 21% had a child protection notification within 2 days before or 5 days after presentation (of which 87% were substantiated).<br>0.2% of all presentations for intestinal injury. 14% of these had a hospital admission and 6% had a child protection notification within 2 days before or 5 days after presentation (of which 76% were substantiated). |

| | | | | | | |
|---|---|---|---|---|---|---|
| Patton (2019)<br><br>USA | **Purpose:** Descriptive<br><br>**Aims:** To identify the social determinants that have the greatest impact on Medicaid expenditures in adolescence<br><br>**Outcomes:** Health care costs (Medicaid expenditures) | 1. **Health** (Department of Social and Health Services (incl. developmental disabilities, mental health, substance abuse records), Health Care Authority (Medicaid), Department of Health birth and death records); **justice** (State Patrol (arrests), Administrative Office of the Courts (filings, adjudications, convictions), Department of Corrections (prison incarcerations); **social services** (Department of Social and Health Services economic services records and CPS records)<br>2. No<br>3. Yes<br>4. Yes<br>5. 0-17 years (costs measured from 12-17) | Dataset from larger repeated one-time/near real-time database. Linkage technique (probabilistic vs. deterministic) ND. Data were provided to researchers in de-identified format. 77.7% of young people were able to be linked to a parent (no additional detail on linkage validation/quality assessment). | N = 181,176 Medicaid-enrolled young adults aged 12-17 years during first month of coverage. 49.2% female; mean age = 14.4 years (SD 1.70); 42.8% non-Hispanic White, 29.2% Hispanic, 15.0% multiple races (non-Hispanic), 5.1% non-Hispanic Black, 2.7% non-Hispanic Asian, 1.7% non-Hispanic Indian, 1.1% non-Hispanic Pacific Islander, 2.3% unknown. 40.9% had an allegation of abuse/neglect (17.3% physical abuse, 6.9% sexual abuse, 36.9% neglect); 8.8% had ≥1 out-of-home placement. 16.7% had (a) parent(s) involved in domestic violence. | Allegations of maltreatment that generated an intake, regardless of investigation or substantiation (sexual abuse, physical abuse, neglect, out-of-home placements); parent involvement in domestic violence [Exposure] | 55.2% of children with any abuse or neglect and 15.0% with abuse or neglect in the prior year had health care costs in the top decile (for behavioural health costs, these estimates increased to 68.0% and 24.8%, respectively). Factors most predictive of higher health care costs were child abuse (especially sexual abuse), child neglect, and instability of out-of-home placements (parental involvement in domestic violence was predictive for higher overall medical system costs ($p<0.05$), but not behavioural health costs). Increased medical costs for children who had experienced maltreatment were primarily driven by increased use of behavioural health services. |

| | | | | | | |
|---|---|---|---|---|---|---|
| Prince (2019)<br><br>USA | **Purpose:** Descriptive<br><br>**Aims:** To estimate the impact of state and individual-level risk and protective factors on adverse 19-year-old outcomes among a cohort of U.S. transition age youth<br><br>**Outcomes:** Adverse outcome at age 19 (homelessness, incarceration, substance abuse referral, childbirth) | 1. **Social services** (Administration for Children and Families (National Youth in Transition Database general release files), Adoption and Foster Care Analysis and Reporting System, Comprehensive Housing Affordability Strategy); **other aggregate** (U.S. Department of Housing and Urban Development American Community Survey)<br>2. No<br>3. No<br>4. No<br>5. 0-19 years (risk factors self-reported at age 17) | Could not categorise due to insufficient description of linkage techniques. Linked on unique case IDs. Linkage validation/quality assessment ND. | N = 7449 young people in foster care placement aged 17-19 (N = 4991 present in the services file). 45.4% White, 29.3% Black, 17.4% Latino/a, 8.0% other. 56.2% had a removal reason of child maltreatment. | Child maltreatment (physical abuse, sexual abuse, and/or neglect) as reason for removal from care [Exposure] | At age 19, 24.0% had experienced homelessness in the last two years, 14.4% had a substance abuse referral, 22.4% had been incarcerated, 12.3% had a childbirth. Compared with children removed due to maltreatment, children removed due to behavioural/emotional problems were more likely to experience homelessness (adj. OR = 1.44, 95% CI 1.17, 1.78), have criminal justice involvement (adj. OR = 1.46, 95% CI 0.99, 2.15), or have a substance abuse referral (adj. OR = 1.46; 95% CI 1.14, 1.87), but not to have a childbirth outcome. Children removed due to other reasons had a higher chance of homelessness than those removed due to maltreatment (adj. OR = 1.36, 95% CI 1.15, 1.59) but not any other outcome. There were no significant differences in outcome for children removed for parental substance abuse compared with child maltreatment. |
| Putnam-Hornstein (2011b)<br><br>USA | **Purpose:** Descriptive<br><br>**Aims:** To examine a prior, nonfatal allegation of maltreatment as an independent risk factor for intentional injury mortality as well as unintentional injury mortality<br><br>**Outcomes:** Intentional/ unintentional injury fatality before age 5 | 1. **Health** (vital birth and death records); **social services** (CPS records)<br>2. No<br>3. Yes<br>4. No<br>5. 0-5 years | Retrospective, one-time linkage (per source) using probabilistic linkage technique. Linked on personal identifiers e.g. child's first and last name, date of birth, gender, maternal first and last name. Extensive manual review of matches; match rates varied between data sources (98.1% match rate for birth to death records) and across years (84.6- | All live births in California 1999-2006 (N = 4,317,321). 48.8% female; maternal race/ethnicity 50.8% Hispanic, 31.3% White, 11.8% Asian/Pacific Islander, 6.1% Black. 11.9% had an allegation of maltreatment. | Maltreatment allegations regardless of whether reports were screened in/out [Exposure] | N = 1917 children died by injury in the study period. 75.0% of these died by unintentional injury, 19.9% by intentional (assault/maltreatment-related injury), and 5.1% had undetermined intent. Children with a prior maltreatment allegation were more likely than those without an allegation to die by injury (adj. HR = 2.59, 95% CI 2.27, 2.97). Greater risk was especially pronounced in intentional injuries (adj. HR = 5.86, 95% CI 4.39, 7.81) compared with unintentional injuries (adj. HR = 2.00, 95% CI 1.71, 2.35). Prior CPS allegations were the strongest independent predictor of injury mortality. |

| | | | | | | |
|---|---|---|---|---|---|---|
| | | | 91.9% match rate for CPS to birth records). | | | |
| Putnam-Hornstein (2014)<br><br>USA | **Purpose:** Descriptive<br><br>**Aims:** To determine incidence of first and repeat births among girls who were in foster care at age 17<br><br>**Outcomes:** First and repeat teenage births (before age 20) | 1. **Health** (vital birth records); **social services** (CPS records)<br>2. No<br>3. Yes<br>4. Yes<br>5. 0-20 years | Retrospective, one-time linkage using probabilistic linkage technique. Linked on a combination of unique (social security number) and non-unique (first name, last name, date of birth) identifiers. Linkage validation/quality assessment ND. | N = 20,222 girls in foster care at age 17 between 2003-2007. 35.0% Latina, 32.4% Black, 28.6% White, 4.0% other/missing. Removal reasons were 68.7% neglect, 12.5% physical abuse, 8.6% sexual abuse, 10.3% other/missing. | Abuse (incl. physical and sexual abuse) or neglect as reason for removal from care [Exposure (covariate)] | 11.4% of girls gave birth before age 18 and 28.1% before age 20 (cumulative rates). Compared with girls removed due to neglect, girls removed due to physical abuse (crude RR = 0.66, 95% CI 0.57, 0.75), sexual abuse (crude RR = 0.76, 95% CI 0.65, 0.88), or other/missing reasons (crude RR = 0.72, 95% CI 0.62, 0.83) were less likely to give birth before age 18. For physical and sexual abuse, this relationship was not significant for births before age 20 (crude RR for other/missing removal reason compared with neglect = 0.84, 95% CI 0.77, 0.91). There was no relationship between removal reason and risk of repeat birth. |
| Putnam-Hornstein (2013c)<br><br>USA | **Purpose:** Descriptive<br><br>**Aims:** To examine variations in children's risk of an unintentional or intentional fatal injury following an allegation of physical abuse, neglect, or other maltreatment<br><br>**Outcomes:** Fatal injury before age 5 | 1. **Health** (vital birth and death records); **social services** (CPS records)<br>2. No<br>3. Yes<br>4. No<br>5. 0-5 years | Retrospective, one-time (per source) linkage using probabilistic linkage technique. Linked on personal identifiers e.g. child's first and last name, date of birth, gender, maternal first and last name. Match rate for death records to birth records = 98.1%, for CPS records to birth records 86.6%. | All live births in California 1999-2006 (N = 4,317,216). 48.9% female; maternal race/ethnicity 50.1% Latina, 31.3% White, 11.8% Asian/Pacific Islander, 6.0% Black, 0.8% Native American. 11.9% had an allegation of maltreatment (2.0% physical abuse, 7.7% neglect, 2.3% other maltreatment). | Allegations of maltreatment (coded hierarchically: (1) physical abuse, (2) neglect, and (3) other maltreatment (incl. emotional abuse, sexual abuse, abuse of a sibling), regardless of investigation/ substantiation [Exposure] | N = 392 children had a fatal injury before age 5, of which 31.4% were determined to be intentional. Compared with children with allegations of neglect, those with allegations of physical abuse had a higher risk of all injury deaths (adj. HR = 1.70, 95% CI 1.34, 2.17) and intentional injury deaths (adj. HR = 5.22, 95% CI 3.61, 7.57), and a lower risk of unintentional injury deaths (adj. HR = 0.59, 95% CI 0.39, 0.90). Compared with children with allegations of neglect, those with allegations of 'other maltreatment' had a lower rate of all injury deaths (adj. HR = 0.27, 95% CI 0.17, 0.42), intentional injury deaths (adj. HR = 0.18, 95% CI 0.05, 0.56), and unintentional injury deaths (adj. HR = 0.91, 95% CI 0.84, 0.99). |

| | | | | | | |
|---|---|---|---|---|---|---|
| Rhodes (2013)<br><br>Canada | **Purpose:** Descriptive<br><br>**Aims:** To identify factors associated with repeat emergency department presentations for suicide-related behaviours ('repetition') among children/youth to aid secondary prevention initiatives. To compare rates of repetition in children/youth with substantiated maltreatment requiring removal from their parental home with their peers in the general population<br><br>**Outcomes:** Repeat emergency department suicide-related behaviour presentation(s) | 1. Ontario Registered Persons Data Base: **health** (National Ambulatory Care Recording System, Discharge Abstract Database); **social services** (Crown wards database); **other aggregate** (Statistics Canada Postal Conversion File)<br>2. No<br>3. Yes<br>4. No<br>5. 0-18 years (index presentations 12-17 years) | Retrospective, one-time linkage using probabilistic linkage technique. Linked on date of birth, full name, and sex. 95% match rate. | N = 6305 children aged 12-17 years with an emergency department presentation for suicide-related behaviours (N = 179 Crown wards). Full cohort 72.21% female; maltreatment cohort 64.25% female. | Substantiated maltreatment (resulting in removal from home ('Crown wards')) [Exposure] | Youth removed from home due to substantiated maltreatment (Crown wards) were two times as likely (adj. HR = 2.0, 95% CI 1.59; 2.53) to have ≥1 repetition(s) than their peers when adjusting for potential confounders. |

| | | | | | | |
|---|---|---|---|---|---|---|
| Rhodes (2012)<br><br>Canada | **Purpose:** Descriptive<br><br>**Aims:** To determine whether the rates of a first presentation to the emergency department for suicide-related behaviour are higher among children/youth permanently removed from their parental home because of substantiated maltreatment than their peers. To describe the health care settings accessed by these children/youth before a first suicide-related behaviour presentation.<br><br>**Outcomes:** First emergency department presentation for suicide-related behaviour and prior health care use | 1. Ontario Registered Persons Data Base: **health** (National Ambulatory Care Recording System, Discharge Abstract Database); **social services** (Crown wards database); **other aggregate** (Statistics Canada Postal Conversion File)<br>2. No<br>3. Yes<br>4. No<br>5. 0-17 years (presentations aged 12-17 years) | Retrospective, one-time linkage using probabilistic linkage technique. Linked on date of birth, full name, and sex. 95% match rate. | N = 1,039,229 children in Ontario aged 12-17 years (N = 4,683 Crown wards and N = 1,034,546 peers). | Substantiated maltreatment (resulting in removal from home ('Crown wards')) [Exposure] | Youth removed from home due to substantiated maltreatment (Crown wards) were five times as likely (adj. HRs = 5.13, 95% CI 3.94, 6.68 for boys and 5.36, 95% CI 4.40, 6.54 for girls) to have a first emergency department presentation for a suicide-related behaviour than their peers when adjusting for potential confounders. |

| | | | | | | |
|---|---|---|---|---|---|---|
| Rose (2017)<br><br>USA | **Purpose:** Descriptive<br><br>**Aims:** To examine the predictors of psychiatric residential treatment facility entry among a state-wide sample of children in families investigated for maltreatment<br><br>**Outcomes:** Entrance into a psychiatric residential treatment facility | 1. **Health** (Medicaid); **social services** (child abuse/neglect reports and foster care placement records, Temporary Assistance for Needy Families (income maintenance))<br>2. No<br>3. Yes<br>4. Yes<br>5. 5-17 years | Retrospective, one-time linkage using probabilistic linkage technique. Linked on common identifiers, names, birthdates, Social Security Numbers. Linkage validation/quality assessment ND. | N = 105,982 children whose families were investigated for maltreatment. 51.1% female; 46.8% White, 36.2% Black, 10.7% Hispanic, 1.4% American Indian/Alaskan Native, 0.6% Asian, 0.2% Native Hawaiian/Pacific Islander. 1.5% had a substantiated report of abuse, 7.9% of neglect, 1.4% of abuse and neglect (mean age at first maltreatment report = 11.6 years (SD 3.2)) | Child maltreatment (abuse/neglect) investigations and substantiations [Exposure] | N = 1646 (1.6%) of children entered into a psychiatric residential treatment facility. Clinical/health care-related factors that predicted entrance into a psychiatric residential treatment facility included diagnosis for a trauma-related behavioural health diagnosis (adj. HR = 3.9, 95% CI 2.9, 5.3), antipsychotic medication prescriptions (adj. HR = 8.7, 95% CI 7.0, 10.7), residential treatment (adj. HR = 4.2, 95% CI 3.5, 4.9), and secure residential treatment (adj. HR = 1.7, 95% CI 1.3, 2.3). Care-related factors that predicted entrance were prior placement in residential care (therapeutic family and group foster placement, adj. HR = 1.5, 95% CI 1.3, 1.7), placement in foster care for the first time during the study period (adj. HR = 1.9, 95% CI 1.2, 3.1), return to care during the study period (adj. HR = 4.1, 95% CI 1.9, 9.0), and placement in a treatment centre setting during the first foster care spell (adj. HR = 3.1, 95% CI 1.9, 5.1). Factors associated with a lower chance of entrance included exit from foster care during the study period (adj. HR = 0.1, 95% CI 0.1, 0.2), receipt of income assistance (TANF; adj. HR = 0.6, 95% CI 0.5, 0.7), and placement in a therapeutic home during the first foster care spell (adj. HR = 0.6 95% CI 0.4, 0.9). |

| | | | | | | |
|---|---|---|---|---|---|---|
| Rossen (2019)<br><br>Australia | **Purpose:** Descriptive<br><br>**Aims:** To examine the associations between early developmental vulnerabilities and (a) the highest level of child protection response and (b) the developmental timing of the first child protection report<br><br>**Outcomes:** Developmental vulnerability at age 5 | 1. New South Wales - Child Development Study (NSW-CDS): **education** (Australian Government Department of Education Australian Early Development Census (AEDC)); **health** (NSW Registry of Births, Deaths and Marriages Birth Registrations, NSW Ministry of Health's Perinatal Data Collection, Admitted Patient Data Collection, Emergency Department Data Collection, Mental Health Ambulatory Data Collection); **social services** (NSW Department of Family and Community Services Child Protection Case Management System – Key information Directory System (CPS) records)<br>2. No<br>3. Yes<br>4. Yes<br>5. 0-14 years | Dataset from a larger database of repeated one-time/near real-time linkage using probabilistic linkage technique. Linked on personal information including name/alias, date of birth, residential address, sex. Exempt from consent requirements. Data provided to researchers in de-identified format. Quality check showed linkage rates had a false-positive rate of 0.5% | N = 67,027 children enrolled in the New South Wales Child Development Study. 49.4% female; mean age = 5.62 years (SD 0.37). 16.3% had ≥1 child protection report or out-of-home care placement by age 5. 10.7% of reports were coded as domestic violence, 5.0% as physical abuse, and 4.5% as neglect. | Child protection reports, coded hierarchically by most severe presentation: (1) children with an out-of-home care placement, (2) substantiated 'risk of significant harm' reports (risk/actual harm) without out-of-home care placement, (3) unsubstantiated but investigated reports, (4) un-investigated ('screened out') reports [Exposure] | There were medium to large effect sizes across all five developmental domains for all levels of CPS involvement. Children with out-of-home care placements had greatest odds of vulnerability for social (adj. OR = 3.05, 95% CI 2.56, 2.63) and emotional domains (adj. OR = 3.11, 95% CI 2.59, 3.73); children with substantiated reports but no out-of-home placement had greatest odds of vulnerability on physical (adj. OR = 3.19, 95% CI 2.78, 3.65), cognitive (adj. OR = 3.67, 95% CI 3.15, 4.27) and communication domains (adj. OR = 2.77, 95% CI 2.39, 3.21). Compared with children with no CPS involvement, children with any CPS involvement had a greater likelihood of vulnerability on ≥3 domains, with the highest odds for children with substantiated reports (adj. OR = 4.90, 95% CI 4.13, 5.80) and children with out-of-home care placements (adj. OR = 3.93, 95% CI 3.16, 4.90). In terms of timing of reports, children with reports before age 18 months had higher odds than older children of vulnerability on 1, 2, or ≥3 domains (adj. ORs 1.79-3.56). |

| | | | | | | |
|---|---|---|---|---|---|---|
| Ryan (2018)<br><br>USA | **Purpose:** Descriptive<br><br>**Aims:** To investigate the prevalence of early contact with child protection services and to determine whether early exposure to maltreatment investigations was associated with important academic outcomes<br><br>**Outcomes:** prevalence of early contact with child protection services; academic outcomes in 3rd grade (standardised math and reading scores, grade repetition, special education status) | 1. **Education** (records from the Michigan Department of Education); **social services** (Michigan Department of Health and Human Services (CPS records)); **other aggregate** (census data)<br>2. No<br>3. Yes<br>4. No<br>5. 0-approx. 8-9 years (3rd grade) | Retrospective, one-time linkage using deterministic and probabilistic linkage techniques. Linked on first name, last name, date of birth, and gender. Data provided to researchers in de-identified format. 87.6% of CPS records were successfully matched and 92% of matches were included due to being rated as a 'definite match' or 'very high certainty' match. | All children born in Michigan from 2000-2006 attending public schools (N = 732,828). 49.3% female; approx. age at 3rd grade = 8-9 years; 68.7% White, 19.4% Black, 7.2% Hispanic, 3.5% Asian, 1.2% other race/ethnicity. 11.3% had ≥1 unsubstantiated investigation (but no substantiated investigation) and 6.4% had ≥1 substantiated investigation. 83.8% were investigated for neglect, 21.9% for physical abuse, 3.8% for sexual abuse. | CPS involvement before 3rd grade: formal investigation of a report of abuse and/or neglect, including substantiated and unsubstantiated investigations. Three mutually exclusive categories: (1) no investigation, (2) ≥1 unsubstantiated investigation and 0 substantiated investigations, and (3) ≥1 substantiated investigation [Exposure/ Outcome, depending on research question] | 17.7% of children attending public schools had a formal investigation by the end of 3rd grade, 36.0% of which were substantiated investigations. Rates of investigations were higher among more disadvantaged groups (e.g. those receiving free school lunch, those from the poorest neighbourhoods). Black students also had experienced proportionally more investigations. Compared with children with no CPS involvement, children with investigations for maltreatment (regardless of substantiation) had poorer academic outcomes. Children with ≥1 *unsubstantiated* investigation had significantly lower scores on standardised math ($\beta$ = -0.15, 95% CI: -0.17, -0.13) and reading ($\beta$ = -0.16, 95% CI: -0.18, -0.14) scores and were more likely to be in special education (adj. OR = 1.34, 95% CI: 1.30, 1.37) and repeat a grade (adj. OR = 1.32, 95% CI: 1.29, 1.36). Children with ≥1 *substantiated* investigation had significantly lower scores on standardised math ($\beta$ = -0.19, 95% CI: -0.21, -0.17) and reading ($\beta$ = -0.20, 95% CI: -0.22, -0.18) scores and were more likely to be in special education (adj. OR = 1.44, 95% CI: 1.39, 1.50) and repeat a grade (adj. OR = 1.40, 95% CI 1.35, 1.45). |

| | | | | | | |
|---|---|---|---|---|---|---|
| Schuck (2005)<br><br>USA | **Purpose:** Descriptive<br><br>**Aims:** To investigate how neighbourhood conditions influence the relationship between early child maltreatment and criminal behaviour<br><br>**Outcomes:** Criminal behaviour (juvenile and adult arrests) | 1. **Justice** (juvenile and adult arrest data); **social services** (CPS records); **other aggregate** (census-level data on neighbourhood characteristics)<br>2. No<br>3. Yes<br>4. No<br>5. 0-mid-30s | Could not categorise due to insufficient description of linkage techniques. Implied exemption from consent requirements. Linkage validation/quality assessment ND. | N = 1575 (N = 908 children with substantiated maltreatment and N = 667 matched controls). Maltreated group: 49% female; 67% White, 31% African American. Controls: 50% female; 65% White; 35% African American. | Substantiated maltreatment (incl. physical abuse, sexual abuse, and neglect) before age 11) [Exposure] | Children with substantiated maltreatment were more likely than controls to have a criminal arrest. Adjusting for residential stability increased the interaction term between abuse/neglect and disadvantage, i.e. the relationship between maltreatment and offending was greatest for those from the most disadvantaged and most stable neighbourhoods. There was a significant, positive interaction between neighbourhood concentrated disadvantage and neglect and a significant, negative interaction between sexual abuse and neighbourhood concentrated disadvantage. There was no significant interaction between physical abuse and concentrated disadvantage. No other neighbourhood measure (residential stability, ethnic heterogeneity, or concentrated advantage) had a significant interaction with any type of abuse/neglect. |

| | | | | | | |
|---|---|---|---|---|---|---|
| Scott (2010)<br><br>New Zealand | **Purpose:** Descriptive<br><br>**Aims:** To estimate associations between prospectively-ascertained child maltreatment and a wide range of subsequently measured DSM-IV mental disorders and to show the influence of retrospectively -reported maltreatment in the comparison group on these associations<br><br>**Outcomes:** DSM-IV mental health disorders (incl. anxiety disorders, mood disorders, and substance abuse disorders) | 1. **Social services** (Child, Youth and Family agency (CPS) records); **study-specific** (New Zealand Mental Health Survey)<br>2. Yes<br>3. No<br>4. No<br>5. Maltreatment ascertained in CPS records from 0-17 years (retrospective recall as an adult, aged 16-27) | Retrospective, one-time linkage. Linkage technique (probabilistic vs. deterministic) ND. Linked on name, date of birth, and survey number. Written informed consent obtained for linkage. 5% of survey sample was not linked due to missing name (but missing at random). | N = 2144 respondents to the New Zealand Mental Health Survey (aged 16-27). 10.3% had prospectively-ascertained maltreatment (i.e. documented in CPS data); of these, 57.8% female; mean age at survey = 21 years; 49.2% Māori, 5.4% Pacific, 55.4% other ethnicity. 16.7% had retrospectively-ascertained maltreatment only (i.e. recalled in survey but did not have documented maltreatment in CPS data). | *As measured in survey:* physical abuse, child rape/sexual abuse, witnessing intimate partner violence; *as measured in CPS files:* screened-in reports of maltreatment [Exposure] | Compared with those who did not have a CPS-documented history of maltreatment (but including those who self-reported maltreatment without a CPS file), participants with CPS files had an increased 12-month prevalence of any mental health disorder (adj. OR = 2.32, 95% CI 1.39, 3.85), any mood disorder (adj. OR = 1.86, 95% CI 1.12, 3.08), any anxiety disorder (adj. OR = 2.41, 95% CI 1.47, 3.97), and any substance use disorder (adj. OR = 1.71, 95% CI 1.01, 2.88). This was also true for lifetime prevalence of any mental health disorder (adj. OR = 2.12, 95% CI 1.20, 3.75), any mood disorder (adj. OR = 1.80, 95% CI 1.21, 2.68), any anxiety disorder (adj. OR = 2.04, 95% CI 1.24, 3.33), or any substance use disorder (adj. OR = 2.38, 95% CI 1.55, 3.65). Associations increased in magnitude when those who self-reported maltreatment (but who did not have a CPS file) were removed from the comparison group. |

| | | | | | | |
|---|---|---|---|---|---|---|
| Spataro (2004)<br><br>Australia | **Purpose:** Descriptive<br><br>**Aims:** To examine the association between child sexual abuse in both boys and girls and subsequent treatment for mental disorder<br><br>**Outcomes:** Mental health disorder treatment | 1. **Health** (Victorian Psychiatric Case Register, Victorian Institute of Forensic Medicine); **other aggregate** (Australian Bureau of Statistics population data)<br>2. No<br>3. Yes<br>4. No<br>5. Sexual abuse 0-16 years; 9-year follow-up (mean age 27.1 years (SD 13.3)) | Retrospective one-time linkage using probabilistic linkage technique. Linked on full name and date of birth. Implied exemption from consent requirements. Manual linkage assessment showed >90% agreement between algorithm- and hand-matched linkages; independent raters favoured the algorithm linkages in all discrepant cases. | N = 3,141,357 Australian-born children 1950-1991 (N = 1612 children who were sexually abused and N = 3,139,745 controls). Sexual abuse cohort: 82.3% female. | Sexual abuse (identified on the basis of findings from the Office of Forensic Medicine case files) [Exposure] | 12.4% of the sexual abuse cohort vs. 3.6% of general population controls had contact with public mental health services for a mental health disorder (RR = 3.8, 95% CI 3.2, 4.4). For adult mental health disorders, the highest relative risks for sexually abused children compared with population controls were for personality disorders (RR = 4.7, 95% CI 2.3, 9.4) and anxiety and acute stress disorders (RR = 3.2, 95% CI 2.2, 4.5); for childhood mental health disorders, highest risks were for conduct disorders (RR = 7.2, 95% CI 3.4, 15.0) and other disorders (RR = 7.7, 95% CI 2.7, 13.4). Rates of service use without recorded diagnosis was higher for the sexual abuse cohort than population controls (RR = 5.4, 95% CI 4.3, 6.8). Rates of contact were significantly higher in both sexually abused males (RR = 7.2, 95% CI 5.5, 9.6) and females (RR = 3.3, 95% CI 2.8, 3.9) compared with controls and males were significantly more likely than females to have contact with public mental health services (22.8% vs. 10.2%, p<0.001). Males had significantly higher contact for childhood but not adulthood disorders. |

| | | | | | | |
|---|---|---|---|---|---|---|
| Valuri (2020)<br><br>Australia | **Purpose:** Descriptive<br><br>**Aims:** To examine the impact of substance use and other risk factors on conviction rates in people with a psychotic illness and other mental disorders compared to those with no mental illness<br><br>**Outcomes:** Conviction rates | 1. **Health** (births register, Western Australian Death Register, Western Australian Mental Health Information System); **justice** (Western Australian Department of Corrective Services); **social services** (Western Australian Department of Communities database (CPS records))<br>2. No<br>3. Yes<br>4. Yes<br>5. 0-26 years | Dataset from a larger database with near real-time linkage using probabilistic linkage technique. Accuracy of linkage is high (true matches estimated at 97-99%). | N = 184,147 children born in Western Australia 1980-2001 to mothers with and without a psychotic illness. 48.7% female; 6.1% Indigenous. 1.9% had a substantiated report of maltreatment. | Substantiated allegations of maltreatment (sexual abuse, physical abuse, emotional abuse, and neglect) [Exposure (covariate)] | 8.1% of those with ≥1 conviction and 1.5% of those without a conviction had a substantiated report of maltreatment. Those with a substantiated report were more likely than those without to have ≥1 conviction (adj. IRR = 1.71, 95% CI 1.57, 1.86). |
| Weiss (2001)<br><br>USA | **Purpose:** Descriptive<br><br>**Aims:** To investigate the impact of multiple health and caretaking risk factors on the school adjustment of first-grade students<br><br>**Outcomes:** School adjustment | 1. **Health** (State Health Department birth records, Department of Public Health lead screening records); **education** (school district outcome data); **social services** (Department of Human Services CPS records);<br>2. No<br>3. Yes<br>4. No<br>5. 0-1st grade (approx. 6-7 years) | Retrospective, one-time (per source) linkage using deterministic and probabilistic linkage techniques. Linked on child's first and last names, birth date, and sex. Data were de-identified. Manual review of unmatched records. | N = 9,088 first grade pupils. 49.8% female; mean age = 7.1 (SD 0.30); 69.0% African American, 28.1% White, 2.4% Asian, 0.3% other non-White. 5.3% had a substantiated maltreatment report in the 4 years prior to the study. | Substantiated child protection report [Exposure] | In bivariate analyses, experience of maltreatment increased the likelihood of poor outcomes in terms of academics (OR = 1.76, $p<0.0001$), behaviour (OR = 1.63, $p<0.0001$), retention (OR = 1.94, $p<0.0001$), and attendance (OR = 1.80, $p<0.0001$). There were no significant interaction effects involving maltreatment on any of the four outcomes. |

| | | | | | | |
|---|---|---|---|---|---|---|
| Wong (2017)<br><br>Australia | **Purpose:** Descriptive<br><br>**Aims:** To investigate the proportion of students who were in each of five participation categories (participated, absent, exempt, withdrawn, and not enrolled), out of the population of all eligible school students. To investigate the background risk factors associated with an increased risk of students being absent on the day of testing for enrolled students.<br><br>**Outcomes:** Test day category (participated, absent, exempt, withdrawn, and not enrolled) | 1. Western Australian Data Linkage System: **education** (Department of Education National Assessment Program—Literacy and Numeracy data); **health** (Midwives Notification System, Birth Register, Hospital Morbidity Data Collection, Mental Health Information System, Mortality Register, Intellectual Disability Exploring Answers data set); **social services** (Child Protection and Family Support (CPS) data set)<br>2. No<br>3. Yes<br>4. Yes<br>5. 0 to Year 9 (~14-15 years old) | Retrospective, one-time linkage using probabilistic linkage technique (from a larger 'living' research database using multiple/updated linkages). Linked on medical record number, first and last name, initial, date of birth, sex, and addresses. Data were de-identified. Linkage quality assessed through clerical review of a sub-sample of matches; very low (~0.1%) false positive and false negative matches. | N = 49,034 individuals born in Western Australia 1994 or 1995. 49.0% female; 6.2 % Aboriginal children. 9.5% of Aboriginal children and 1.3% of non-Aboriginal children had a substantiated maltreatment allegation. | Substantiated maltreatment allegations [Exposure] | Substantiated allegation of child maltreatment predicted absence on the day of the Year 9 exam for non-Aboriginal (adj. OR = 1.83, 95% CI 1.37, 2.45 and Aboriginal children (adj. OR = 1.65, 95% CI 1.13, 2.40). |
| **Intergenerational transmission** | | | | | | |

| | | | | | | |
|---|---|---|---|---|---|---|
| Eastman (2019a)<br><br>USA | **Purpose:** Descriptive<br><br>**Aims:** To identify children born to mothers in foster care and document CPS involvement among children<br><br>**Outcomes:** Maltreatment reports before age 3 | 1. **Health** (birth records); **social services** (CPS records)<br>2. No<br>3. Yes<br>4. Yes<br>5. Infants: 0-3 years; mothers under 21 years | Repeated one-time linkages using a probabilistic linkage technique. Linked on unique identifier assigned to each birth and mother and child variables. Match quality checks were conducted using birth spacing and dates of CPS reports compared with children's birth dates; additional case narrative review to confirm foster care and CPS involvement (45 randomly-selected records). | N = 2094 mother child dyads. 64.6% of mothers were minors at the time of giving birth; 51.6% Hispanic, 24.9% Black, 14.4% White, 9.0% other. 64.4% of mothers had a sexual abuse allegation. 5.62% of offspring had a maltreatment report in the first 3 years of life. | Infants: report for alleged abuse or neglect during first three years of life (also measured substantiations and removals) [Outcome].<br><br>Mothers: alleged sexual abuse from age 9 to giving birth [Exposure (covariate)] | 52.6% of children born to mothers in care had a maltreatment report in the first three years of life (90% of which were investigated), 25.8% of children had substantiated reports of maltreatment, and 18.8% were placed in foster care. Factors that significantly increased risk of report (in bivariate analyses) included younger age at birth ($p<0.001$), missing paternity ($p = 0.003$), maternal mental health condition ($p = 0.002$) runaway history ($p<0.001$), recent entrance into care ($p<0.001$), and maternal history of alleged sexual abuse ($p<0.001$). Latent class analysis identified three classes of mother-infant dyads with differential risk for reports: class 1 ('non-minor mothers with stable placements'; 30.1% of dyads), class 2 ('minor mothers with short placements'; 46.6% of dyads), and class 3 ('mothers with unstable placements and mental health conditions'; 23.3% of dyads). Class 1 had the lowest probability of maltreatment report (36%, 11.0% substantiated), then class 2 (55%, 19.6% substantiated), and class 3 (68%, 43.1% substantiated). |

| | | | | | | |
|---|---|---|---|---|---|---|
| Font (2020b)<br><br>USA | **Purpose:** Descriptive<br><br>**Aims:** To investigate the risk of multiple forms of parent or perpetrator CPS involvement (PP-CPS) by age 25, among those exposed to three forms of adversity in their late teens (at ages 14–17): alleged victim on a CPS investigation, out-of-home care (OHC), and poverty<br><br>**Outcomes:** Parent or perpetrator CPS involvement by age 25, defined as parent-perpetrator, resident parent non-perpetrator, nonresident parent non-perpetrator, and non-biological parent-perpetrator | 1. Wisconsin Administrative Data Core (WADC): **Health** (Medicaid); **social services** (Child Protective Services Records), Supplemental Nutrition Assistance Program (SNAP; food stamps), Out-of-Home Care records, child support, public assistance)<br>2. No<br>3. Yes<br>4. Yes<br>5. 14-25 years (maltreatment between 14 and 17 years) | Retrospective, one-time linkage using deterministic and probabilistic linkage techniques (from a larger research database that is routinely updated). Linked on child and parent identifiers. Implied exemption from consent requirements. Manual checks of false matches (described in detail on the WADC website). | N = 36,475 individuals born in 1990–1991 who were (1) in out-of-home care (OHC group, N = 3203), (2) had CPS involvement as a victim but no out-of-home care (CPSV group, N = 8254), or (3) received food assistance but did not fall into either of the other groups (SNAP group, N = 25,018) from age 14-17 years. 51.8% female. No further characteristics provided. | Substantiated and unsubstantiated CPS investigations (as a proxy for child maltreatment) [Exposure/ outcome] | 16.0% of females and 13.0% of males experienced PP-CPS involvement during young adulthood. In the OHC group, 25% of men and 33% of women had PP-CPS involvement. In the CPSV group, 17% of men and 23% of women had PP-CPS involvement. In the SNAP group, 10% of men and 10% of women had PP-CPS involvement. Women had higher rates of parent perpetrator and resident parent non-perpetrator involvement; men had higher rates of non-biological parent perpetrator and nonresident parent non-perpetrator involvement.<br><br>Males in CPSV (adj. OR = 1.95, $p<0.001$) and OHC (adj. OR = 3.30, $p<0.001$) groups more likely than those in SNAP group to experience any PP-CPS involvement before age 25. Those in both groups had higher odds of all types of PP-CPS. OHC males were more likely than CPSV males to have nonresident parent non-perpetrator and non-biological child perpetrator CPS involvement.<br><br>Females in CPSV (adj. OR = 2.58, $p<0.001$) and OHC (adj. OR = 4.56, $p<0.001$) groups more likely than those in SNAP group to experience any PP-CPS involvement before age 25. Those in both groups had higher odds of all types of PP-CPS. OHC females had sig. higher odds for each type of PP-CPS involvement than those with CPSV. |

| | | | | | | |
|---|---|---|---|---|---|---|
| Galos (2017)<br><br>USA | Study 1:<br><br>**Purpose:** Descriptive<br><br>**Aims:** To estimate the proportion of victims of child maltreatment who become offenders in adulthood To identify risk factors for and demographic correlates of intergenerational maltreatment.<br><br>**Outcomes:** intergenerational transmission of maltreatment<br><br>Study 2:<br>**Purpose:** Descriptive<br><br>**Aims:** To examine the association of intergenerational child maltreatment with three dimensions of academic achievement: standardised testing, attendance, and mobility.<br><br>**Outcomes:** academic achievement (standardised testing, attendance, mobility)<br><br>Study 3:<br>**Purpose:** Methodological | Study 1:<br>1. **Education; social services** (CPS records)<br>2. No<br>3. Yes<br>4. No ('transmission' of maltreatment measured by offender status in adults)<br>5. 0-32 years<br><br>Study 2:<br>1. **Education; social services** (CPS records)<br>2. No<br>3. Yes<br>4. No (maltreatment for caregivers defined based on their self-report during CPS response to their child's maltreatment report)<br>5. 3rd-8th graders (approx. 8-13 years old)<br><br>Study 3:<br>1. **Social services** (CPS records, out-of-home placement records, risk assessment records)<br>2. No<br>3. Yes<br>4. No<br>5. ND | All studies:<br>Retrospective linkage using probabilistic linkage technique; one-time linkage (multiple one-time linkages for different sources; 1 per study). Linked on names and birth dates (Studies 1 and 2) or offender/work group identifier (Study 3). Data were provided to researchers in de-identified format (identifiable data used in linkage). Manual review of uncertain matches. | Study 1: N = 8701 Minnesota residents with maltreatment reports before the age of 13. 59.4% female; 55.7% White, 25.3% Black, 3.7% Asian/Pacific Islander, 9.3% American Indian/Alaska Native, 6.1% Latino/Hispanic. 24.4% had experienced neglect, 13.6% physical abuse, 6.0% sexual abuse, and 10.6% multiple types of maltreatment.<br><br>Study 2: N = 7006 children who had accepted reports of maltreatment (N = 3144 with and N=3862 without intergenerational maltreatment). 48.3% female; 48% non-Hispanic White, 31.9% Black/African American, 12.0% American Indian/Alaska Native, 8.3% Hispanic/Latino, 2.5% Asian/Pacific Islander.<br><br>Study 3: N = 8400 individuals (N = 253 with prior CPS contact and N = 8147 without prior contact). CPS group: 58.1% female; 45.24% had experienced neglect, 20.63% physical abuse, 9.92% sexual abuse, 24.21% multiple types of maltreatment. | All studies: accepted maltreatment report (physical/sexual abuse and neglect where neglect was comprised of medical neglect, nonmedical neglect and mental injury) & additionally self-reported maltreatment for Study 3 [Studies 1/3: exposure and outcome; Study 2: exposure] | Study 1: Maltreatment transmission probability was 11.26% across all groups but varied by type of maltreatment: the highest probability was among those who experienced multiple forms of maltreatment (6.0%), followed by physical abuse (4.6%), neglect (4.4%), and sexual abuse (4.1%). Males were less likely than females to have contact as potential offenders in adulthood (adj. RR = 0.28, 95% CI: 0.24-0.33). Compared with White individuals, Asian/Pacific Islander individuals were less likely to be potential offenders (adj. RR = 0.47, 95% CI 0.29, 0.77), while Native American/American Indian (adj. RR = 1.66, 95% CI 1.40, 1.99) and Black/African American (adj. RR = 1.49, 95% CI 1.30, 1.70) individuals were more likely to be. Out-of-home placement was also associated with higher probability of transmission (adj. RR = 1.36, 95% CI 1.20, 1.53).<br><br>Study 2: Compared with children enrolled in public schools, children involved with CPS had lower Minnesota Comprehensive Assessments scores, higher rates of mobility, and lower attendance. In adjusted models, intergenerational transmission of maltreatment (i.e. children whose parents also had a history of maltreatment) was not significantly associated with any of the academic outcomes in comparison with children with CPS contact but whose parents did not have a history of maltreatment. Associations varied by type of maltreatment.<br><br>Study 3: A significantly ($p<0.01$) smaller |

| | | | | | | |
|---|---|---|---|---|---|---|
| | advancement/quality assessment<br><br>**Aims:** To quantify the amount and direction of misclassification of self-reported history of child maltreatment. To test if misclassification is differential with respect to maltreatment-related risk factors. To test if misclassification is differential across types of maltreatment. To identify whether any demographic groups are more or less likely to underreport.<br><br>**Outcomes:** accuracy of adult recall of child maltreatment | | | | | proportion of caregivers with prior CPS contact (54.55%) reported never being abused than caregivers without prior contact (73.43%). Underreporting was significantly less likely among those with out-of-home care placements, younger people, females, and those with a parent/guardian relationship. |
| Putnam-Hornstein (2015)<br><br>USA | **Purpose:** Descriptive<br><br>**Aims:** To investigate whether maternal maltreatment was associated with heightened rates of reported and/or substantiated offspring abuse and neglect<br><br>**Outcomes:** Reported and/or substantiated maltreatment reports before age 5 | 1. **Health** (California Department of Public Health vital statistics birth records); **social services** (CPS records)<br>2. No<br>3. Yes<br>4. Yes<br>5. Teenaged mothers (age 15-19 years at birth; child maltreatment measured after age 10 and before giving birth only) and their | Retrospective, one-time linkage for mothers and repeated one-time/near real-time linkage for infants using probabilistic linkage technique. Mothers' records linked on personally identifiable maternal data from the birth record; infants' data linked on personally identifiable data. Identifiable data. | N = 85,084 infants born to teenaged (15-19 year old) mothers. Maternal ethnicity: 15.1% White, 8.6% Black, 72.9% Hispanic, 2.9% Asian/Pacific Islander, 0.6% Native American. 28.3% of infants born to mothers with child maltreatment reports (16.6% unsubstantiated, 11.8% substantiated). 23.6% of infants had CPS reports before age 5 (7.8% had substantiated | All reports of child maltreatment (regardless of investigation or substantiation) [Maternal maltreatment = exposure; infant maltreatment before age 5 = outcome] | Maternal history of CPS reports was significantly associated with any offspring CPS report (adj. HR = 2.07, 95% CI 2.00, 2.14 for unsubstantiated maternal reports; 2.62, 95% CI 2.53, 2.72 for substantiated maternal reports) as well as substantiated offspring CPS reports (adj. HR = 2.19, 95% CI 2.06, 2.33 for unsubstantiated maternal reports; 3.19, 95% CI 3.00, 3.39 for substantiated maternal reports). |

| | | | | | | |
|---|---|---|---|---|---|---|
| | | children aged 0-5 years | Linkage validation/quality assessment ND. | reports). | | |
| **Case identification** | | | | | | |
| Green (2019b)<br><br>Australia | **Purpose:** Predictive modelling<br><br>**Aims:** To determine the minimum set of cross-agency indicators that could accurately classify placement in out-of-home care before age 13–14 years<br><br>**Outcomes:** Out-of-home placement before age 13-14 | 1. New South Wales - Child Development Study (NSW-CDS): **education** (Australian Government Department of Education Australian Early Development Census (AEDC)); **health** (NSW Registry of Births, Deaths and Marriages Birth Registrations, Death Registrations, NSW Ministry of Health's Perinatal Data Collection, Admitted Patient Data Collection, Emergency Department Data Collection, Mental Health Ambulatory data collection); **justice** (NSW Bureau of Crime Statistics and Research Reoffending data); **social services** (SW Family and Community Services Child Protection Case Management System – Key Information Directory System (CPS) records)<br>2. No | Repeated one-time linkages using probabilistic linkage technique (data from data linkage centre/linked data resource) -- study used dataset created with a one-time retrospective linkage. Linked on name, date of birth, residential addresses, and sex. Data were de-identified. No consent requirements. Low rates of false-positive linkages for children's data (0.3%) and for linkage of parents to children (0.5%). | N = 72,079 children enrolled in the New South Wales Child Development Study. 48.6% female; mean age = 13.16 (SD 0.37). 21.3% had at least one child protection report but no out-of-home care placement (reported issues were 19.4% carer mental health, 10.7% carer other issues, 12.8% child/young person risk behaviours, 56.0% domestic violence, 19.5% drug/alcohol use by carer, 24.4% emotional abuse, 29.2% neglect, 38.6% physical abuse, 22.5% sexual abuse, 0.4% prenatal report). 1.7% had an out-of-home care placement (and at least one child protection report; reported issues were 56.7% carer mental health, 36.8% carer other issues, 35.4% child/young person risk behaviours, 79.5% domestic violence, 69.0% drug/alcohol use by carer, 65.3% emotional abuse, 80.8% neglect, 81.5% physical abuse, 48.1% sexual abuse, 2.8% prenatal report). | CPS reports had the following categories: carer mental health, carer other issues, child/young person risk behaviours, domestic violence, drug/alcohol use by carer, emotional abuse, neglect, physical abuse, sexual abuse, prenatal report, and other issue [Exposure] | Compared with children with no child protection reports, a single item on the AEDC indicating teachers' knowledge of problems in the home was most highly associated with out-of-home care placements (adj. OR = 7.86, 95% CI 6.06, 10.20). Other significant risk factors included maternal mental health disorder (adj. OR = 6.52, 95% CI 5.54, 7.66), maternal criminal charges (adj. OR = 4.08, 95% CI 3.41, 4.87), mother in custody (adj. OR = 4.48, 95% CI 3.08, 6.51), father in custody (adj. OR=4.64, 95% CI 3.77, 5.70).<br><br>Compared with children with at least one child protection report, parental death was most highly associated with out-of-home care placements (adj. OR = 5.48, 95% CI 3.65, 8.24). Other significant risk factors included maternal mental health disorder (adj. OR = 4.77, 95% CI 4.13, 5.50), mother criminal charges (adj. OR = 3.03, 95% CI 2.58, 3.55), mother in custody (adj. OR = 3.48, 95% CI 2.66, 4.54), and father in custody (adj. OR = 3.75, 95% CI 3.16, 4.45).<br><br>In the final predictive model, a combination of six risk factors (AEDC home environment problems, young maternal age, maternal smoking during pregnancy, maternal mental disorder, mother criminal charge, father in custody) predicted out-of-home care placements with approx. 95% accuracy compared with children with no child |

| | | | | | | |
|---|---|---|---|---|---|---|
| | | 3. Yes<br>4. Yes<br>5. 0-14 years | | | | protection reports. Children with ≥4 of these factors are allocated with a specificity of 99.6% and sensitivity of 45.4%; false positive rate 0.3%, positive predictive value 74.0%) |
| Parrish (2010)<br><br>USA | **Purpose:** Descriptive & predictive modelling<br><br>**Aims:** To determine the additional cases of maltreatment-related infant mortality identifiable through multi-source extraction when compared to vital records alone. To evaluate the usefulness of birth certificate variables for identifying children at increased risk of maltreatment-related infant mortality.<br><br>**Outcomes:** Maltreatment-related mortality before age 1 | 1. Alaska Division of Public Health's Surveillance of Child Abuse and Neglect program (SCAN): **health** (birth and death vital records, community health centres records, Public Health Nursing, Medicaid services, Alaska Maternal-Infant Mortality Review Program); **justice** (Department of Justice, law enforcement); **social services** (CPS records)<br>2. No<br>3. Yes<br>4. Yes<br>5. 0-1 year | Retrospective, one-time linkage. Linkage technique (probabilistic vs. deterministic) ND. Manual review to address data entry errors and incomplete data. | N = 143,025 live births in Alaska. 48.8% female; 24.3% Alaska Native mother, 75.6% non-Native. N = 133 maltreatment-related infant deaths: 45.1% female; 43.5% Alaska Native mother, 56.5% non-Native. | Confirmed/probabl e and possible maltreatment-related mortality before age 1 [Outcome] | N = 133 maltreatment-related infant deaths occurred (61% confirmed/probable' and 39% 'possible'). Only 27% of confirmed/probable cases were identified in vital statistics records (17% when also including possible cases). Maltreatment-specific infant mortality rates were 0.6 per 1000 live births for confirmed/probable cases and 0.9 per 1000 live births for possible cases and were higher for children of unmarried women with missing paternity on the birth certificate (6.7 per 1000 live births) or women with prenatal substance abuse (5.2 per 1000 live births) and infants born with low birth weight (5.4 per 1000 live births). The final model included the following variables (followed by population attributable risk percents): unmarried without a father's name on birth certificate (33.83%), unmarried with a father's name on the birth certificate (32.25%), maternal substance use during pregnancy (25.15%), inadequate prenatal care (12.13%), ≥1 children currently living (34.80%), and low birth rate (12.43%). 97% of maltreatment-related deaths were to families with ≥1 of the following risk factors: family with additional children, unmarried mother, maternal prenatal substance abuse. |

| | | | | | | |
|---|---|---|---|---|---|---|
| Vaithianathan (2013)<br><br>New Zealand | **Purpose:** predictive modelling<br><br>**Aims:** To estimate the likelihood of substantiated maltreatment among children enrolled in New Zealand's public benefit system<br><br>**Outcomes:** Substantiated child maltreatment reports before age 5 | 1. **Social services** (CPS records, New Zealand's public benefit system)<br>2. No<br>3. Yes<br>4. No<br>5. 0-5 years | Retrospective, one-time linkage using probabilistic linkage technique. Linked on personal identifiers. Data were de-identified. Linkage validation/quality assessment ND. | N = 57,986 children born in New Zealand 2003-2006 with a public benefit spell before age 2. 15.0% had a substantiated maltreatment report (6.4% had substantiated neglect, 10.6% emotional abuse, 1.9% physical or sexual abuse). | Substantiated maltreatment reports (incl. neglect, physical abuse, sexual abuse, or emotional abuse) [Outcome] | The final predictive model included 132 variables and had an area under the receiver operating characteristic curve of 76% (95% CI 75.7, 77.1). 47.8% of children in the top decile of risk according to the model had a substantiated maltreatment report by age 5. (vs. 1.7% in the lowest decile). 83% of all children with substantiated maltreatment reports had a spell on public benefit before age 2. |

| | | | | | | |
|---|---|---|---|---|---|---|
| Wilson (2015)<br><br>New Zealand | **Purpose:** Predictive modelling<br><br>**Aims:** To examine technical feasibility and predictive validity of predictive risk models to identify and assess children at risk of abuse or neglect as part of a preventive early intervention strategy<br><br>**Outcomes:** Substantiated child maltreatment before age 5 | 1. **Health** (Births, Deaths and Marriages Register, Ministry of Health data); **justice** (Department of Corrections sentencing data); **social services** (Child, Youth and Family (CPS) records, Ministry of Social Development benefit data)<br>2. No<br>3. Yes<br>4. Yes<br>5. 0-5 years | Retrospective, one-time linkage. Linkage technique (probabilistic vs. deterministic) ND (linked on full name and date of birth). Clerical review of matches with aim of reducing missed matches (at the expense of more false positive linkages). | All live births for New Zealand in 2010 (model development, internal validation) and 2007 (external model validation); approx. 60,000 children in each cohort. | Children: substantiated maltreatment (emotional abuse, physical abuse, sexual abuse, or neglect) [Outcome]<br><br>Parents: parent/caregiver care and protection contact during childhood; police notifications/contact for family violence [Exposures] | The final predictive risk model included 13 of the original 15 variables. The top three predictors were having siblings with contact with care and protection services; length of time on benefits in the previous 5 years; and having a parent/caregiver with a history of care and protection services contact. Other predictor variables included Child, Youth and Family site; benefit caregiver is not birth registration parent; mental health history in last 5 years; police family violence notifications/contact; caregiver's age; corrections history in the last 5 years; single parent; benefits address changed in past year; behavioural or relational difficulties as a child; parenting demands (gender and low birth weight/preterm birth were not included in the final model). The model generally performed well (except for physical abuse): of the top 5% highest scores in the 2007 model: 30.5% would have had a substantiated finding of maltreatment by age 5, 31.6% of all children who had substantiated maltreatment would have been accounted for, and 96.4% of children without eventual substantiations would not have been identified as having risk. Results were similar for Māori children (33.2%, 36.3%, and 91.8%, respectively). Positive predictive value increased with age. |
| **Intervention/service evaluation** | | | | | | |

| | | | | | | |
|---|---|---|---|---|---|---|
| Bruns (2012)<br><br>USA | **Purpose:** Service evaluation<br><br>**Aims:** To examine the outcomes of participants in a family drug treatment court in a large city in the western United States, as compared to participants in the same jurisdiction's regular dependency court<br><br>**Outcomes:** Parental substance use treatment and child welfare outcomes | 1. **Health** (Washington State Division of Behavioral Health and Recovery); **justice** (Family Treatment Drug Court records; County's Superior Court data); **social services** (Washington State Department of Social and Health Services Children's Administration (CPS) records)<br>2. No<br>3. No<br>4. Yes<br>5. ND | Retrospective, one-time linkage. Linkage technique (probabilistic vs. deterministic) ND (linked on indirect identifiers). Active consent obtained. Linkage validation/quality assessment ND. | N = 152 parents (N = 76 family treatment drug court and N = 76 propensity-matched comparisons). Parents: 58% White, 18% African American, 15% American Indian/Alaska Native, 7% Hispanic, 1% Native Hawaiian/Other Pacific Islander, 1% Asian. Children: 57% female; 43% White, 25% African American, 22% American Indian/Alaska Native, 9% Hispanic, 2% unknown. Index petition cause was 85% for neglect, 27% for abuse, and 5% for prenatal injury. | Investigations and substantiations of child maltreatment [Outcome] | Children in the family drug treatment court group spent significantly less time in out-of-home care (median days in care = 476 vs. 689 for comparisons, p=0.03), left the child welfare system earlier (61% left by the end of the study period vs. 43%, p = 0.03), and were more likely to be returned to their guardian after placement (27% vs. 11%, p = 0.016). There were no significant differences in subsequent child welfare investigations, substantiations, or out-of-home placements. |
| Green (2017)<br><br>USA | **Purpose:** Intervention/service evaluation (Healthy Family Oregon home visiting programme)<br><br>**Aims:** To examine the 2-year outcomes of a large-scale randomised study of the impact of the Healthy Families Oregon home visiting programme<br><br>**Outcomes:** Child welfare system involvement (substantiated maltreatment reports, unsubstantiated maltreatment reports, out-of-home | 1. **Health** (Healthy Families Oregon records); **social services** (Oregon Department of Human Services child welfare (CPS) records, self-sufficiency services, substance abuse treatment records); **study-specific** (incl. New Baby Questionnaire)<br>2. Yes<br>3. No<br>4. Yes<br>5. 0-2 years | Repeated one-time linkages using probabilistic linkage technique supplemented by manual linkage. Linked on child and mothers' names, dates of birth, gender, and race/ethnicity. Identifiable data. Parents gave consent for linkage. Linkage validation/quality assessment ND. | N = 2727 children eligible for home visiting (defined as first-time parents of newborn infants with 2+ risk factors such as being teenaged parents, having depressive symptomatology, being unemployed, or having less than a high school education)<br><br>N = 1438 in intervention group: 57.3% White, 27.0% Hispanic/Latino/a, 15.7% other race/ethnicity; 6.3% had at least one substantiated child maltreatment report at 2 years (0.5% had a substantiated physical/sexual abuse | Substantiated and unsubstantiated reports of child maltreatment (including neglect (including emotional abuse, threat of harm, failure to protect, physical/other neglect) and physical/sexual abuse) [Outcome] | No significant differences between groups in the likelihood of having at least one maltreatment report (substantiated or unsubstantiated), having a substantiated report of maltreatment or neglect, having an out-of-home placement, or family reunification at the end of the child welfare case. |

| | | | | | | |
|---|---|---|---|---|---|---|
| | placements); enrolment and utilisation of publicly-funded self-sufficiency and family support services | | | report)<br><br>N = 1289 in control group: 60.4% White, 24.2% Hispanic/Latino/a, 15.4% other race/ethnicity; 6.0% had at least one substantiated maltreatment report at 2 years (0.8% had a substantiated physical/sexual abuse report) | | |
| Hong (2012)<br><br>USA | **Purpose:** Service evaluation (family supportive housing services)<br><br>**Aims:** To investigate the impact of family supportive housing service receipt on children's well-being, including child protection involvement and the academic functioning of homeless children<br><br>**Outcomes:** School attendance rates, school mobility, academic achievement, rates of Individualised Education Plans, and child protection involvement | 1. Minn-LInK project: **education** (Minnesota Department of Education exams data, Minnesota Automated Reporting Student System); **social services** (Social Services Information System (CPS) records, supportive housing records)<br>2. No<br>3. No<br>4. No<br>5. Grades 3-8 (approx. 8-14 years) | Retrospective, one-time linkage using probabilistic linkage technique (from a larger research database performing multiple/updated linkages). Implied exemption from consent requirements. 90% of all children receiving supportive housing services were matched to educational records. | N = 412 (N = 70 children receiving supportive housing services in grades 3-6 at study commencement; N = 342 homeless children not receiving supportive housing services). Supportive housing group: 48.6% female; 60% Black, 21.4% White, 11.4% American Indian, 4.3% Asian/Pacific Islander, 2.9% Hispanic. Comparison group: 50.9% female; 64.3% Black, 19.3% White, 8.5% American Indian, 4.7% Asian/Pacific Islander, 3.2% Hispanic. | Alleged and accepted reports of child maltreatment [Outcome] | A greater proportion of children in the supportive housing group had an accepted maltreatment report in the study period than did those in the comparison group (10% vs. 8%). Yearly rates of child protection involvement decreased over time for the supportive housing group (from 9% to 1%) but not the comparison group (fluctuated between 2-3%). CPS involvement, reports, and substantiations generally decreased over time for the supportive housing group. |

| | | | | | | |
|---|---|---|---|---|---|---|
| Lanier (2014a)<br><br>USA | **Purpose:** Service evaluation (Nurses for Newborns)<br><br>**Aims:** To compare demographic characteristics, risk status, service use, and child maltreatment outcomes in primiparous and multiparous women in a nurse home visiting prevention programme (Nurses for Newborns)<br><br>**Outcomes:** Demographic characteristics, risk status, service use, and child maltreatment | 1. **Health** (Nurses for Newborns service data); **social services** (CPS records)<br>2. No<br>3. No<br>4. Yes<br>5. Prenatal-infancy (up to 47 months after receiving Nurses for Newborns services) | Could not categorise due to insufficient description of linkage techniques. Records linked with patients' consent. | N = 3260 families receiving Nurses for Newborns services. Infants were 48.4% female; 46.0% Black, 3.7% Hispanic (no further information on ethnicity provided). | Infants: child maltreatment reports (regardless of substantiation) [Outcome]<br><br>Mothers: self-reported maltreatment history (rape/sexual abuse and physical abuse/neglect); current partner violence [Exposure] | A significantly greater proportion of multiparous mothers had relevant risk factors than primiparous mothers: 7.5% of multiparous and 3.2% of primiparous mothers experienced current partner violence; 4.5% of multiparous and 3.1% of primiparous mothers reported a history of rape/sexual abuse; 5.3% of multiparous mothers reported a history of physical abuse/neglect vs. 3.8% of primiparous mothers; and 3.0% of children born to multiparous mothers had a prior abuse/neglect report vs. 1.2% of primiparous mothers. Infants of multiparous mothers had a higher risk of later reports of child maltreatment (adj. HR = 1.49, 95% CI 1.16, 1.91). There was a significant trend for more children, greater caregiver stress, maternal depression, and child maltreatment. |
| Lanier (2014b)<br><br>USA | **Purpose:** Intervention evaluation (Parent-Child Interaction Therapy)<br><br>**Aims:** To examine rates of child abuse and neglect reports following a community implementation of Parent–Child Interaction Therapy (PCIT), an evidence-supported intervention for the prevention of maltreatment<br><br>**Outcomes:** Maltreatment reports | 1. **Social services** (CPS records); **study-specific** (Community agency clinical case records)<br>2. Yes<br>3. No<br>4. Yes<br>5. CPS records from birth; intervention participation age range 2-13 years; follow-up ranged from 13 to 40 months | Retrospective, one-time linkage. Linkage technique (probabilistic vs. deterministic) ND (linked on standard state identification number, first name, last name, date of birth). Linkage validation/quality assessment ND. | N = 120 families at-risk for future maltreatment. Children: mean age at first session 6.6 years (SD 2.8). Caregivers: 90% female; mean age = 36.0 years (SD 11.0); 51% White, 42% African American, 7% other (incl. Asian, Indian, American Indian, biracial). 8.3% of parents were maltreatment victims; 22.5% had prior report of maltreatment perpetration (7.5% had substantiated reports). After intervention enrolment, 12.5% of parents had CPS reports of maltreatment perpetration. | CPS reports of victimisation and perpetration of child abuse and neglect [Exposure (past reports) & Outcome (reports since intervention)] | After the first intervention session, 12.5% of caregivers had at least 1 maltreatment report as perpetrators (26.7% of these had ≥2 reports; 20.0% physical abuse and neglect, 46.7% neglect only, 33.3% physical neglect only, no sexual abuse). For those with previous reports, recidivism rates were 37%. Factors predicting a later report included prior victimisation (adj. HR = 38.8, p<0.001) or perpetration of maltreatment (adj. HR = 16.9, p<0.001), and increased parenting stress at baseline (adj. HR = 1.07, p<0.01), but not dosage of intervention or clinical measures. |

| | | | | | | |
|---|---|---|---|---|---|---|
| Larson (2010)<br><br>USA | **Purpose:** service evaluation<br><br>**Aims:** To describe school attendance patterns for children in treatment foster care and whether these vary by age. To determine what improvements to data quality might be employed at the agency level to enhance the ability to facilitate future cross-system matches.<br><br>**Outcomes:** School attendance | 1. Minn-LInK project: **education**; **social services** (Treatment Foster Care records, CPS records)<br>2. No<br>3. No<br>4. No<br>5. 0-18 years | Retrospective, one-time linkage using probabilistic linkage technique. 30.6% match rate for child protection, 78.5% for public education. | N = 673 children receiving Treatment Foster Care. 47.7% female; 50.5% White/Caucasian, 11.7% Native American, 8.2% Black/African American, 4.2% Hispanic 1.2% Asian/Pacific Islander, 8.6% more than one race, 15.6% unknown/missing. | Reports and substantiations of maltreatment [Exposure (covariate)] | Reports and substantiations of maltreatment varied by diagnosis, although differences were not significant. |
| Lee (2017)<br><br>USA | **Purpose:** Descriptive & service evaluation (cash benefits)<br><br>**Aims:** To examine the causal role of income from cash benefits and earnings on reunification<br><br>**Outcomes:** Reunification after out-of-home placement | 1. **Social services** (CPS records, Washington State Department of Social and Health Services economic services, employment services)<br>2. No<br>3. Yes<br>4. No<br>5. 0-21 years for young people, 18-64 years for caregivers | Retrospective, one-time linkage using probabilistic linkage technique (from a larger research database performing multiple/updated linkages). Linked on first and last name, Social Security Number, date of birth, administrative identifiers. 97.6% match rate of primary caregivers. | N = 14,649 cases. 5.4% of children were removed due to sexual abuse, 17.3% to physical abuse, and 59.2% to neglect | Sexual abuse, physical abuse, or neglect resulting in removal from home [Exposure (covariate)] | Modest and inconsistent results suggesting higher earnings are associated with lower likelihood of reunification. |
| Maguire-Jack (2019)<br><br>USA | **Purpose:** Descriptive & service evaluation<br><br>**Aims:** To examine the context in which maltreatment substantiation and out- | 1. **Social services** (National Child Abuse and Neglect Data System (NCANDS), Adoption and Foster Care Analysis and Reporting System | Retrospective, one-time linkage using deterministic linkage technique (linked on AFCARS identifier). Implied exemption from consent | Substantiation: N = 4,110,519 child investigations; 50.0% female; mean age = 8.0 (SD 5.1) years; 45.3% White, 22.5% Black, 1.1% American Indian/Alaskan | Substantiation of investigations for child maltreatment (neglect, physical abuse, sexual abuse, emotional abuse, multiple | Compared with White/non-Hispanic children, Black (adj. OR = 1.03, 95% CI 1.02, 1.04), American Indian/Alaskan Native (adj. OR = 1.20, 95% CI 1.17, 1.24), Native Hawaiian/Pacific Islander (adj. OR = 1.12, 95% CI 1.03, 1.16), multiracial (adj. OR = 1.16, 95% CI 1.14, |

| | | | | | | |
|---|---|---|---|---|---|---|
| | of-home placement occur in the child welfare system, with a specific focus on the extent to which a child's race/ethnicity and the county factors in which the child lives impacts these outcomes<br><br>**Outcomes:** Substantiation of maltreatment and out-of-home placement | (AFCARS)); **other aggregate data** (county-level census data)<br>2. No<br>3. Yes (final sample is a random subset of full population sample)<br>4. No<br>5. 0-18 years | requirements. Linkage improved case identification but details of linkage validation /quality assessment ND. | Native, 0.9% Asian, 0.2% Hawaiian/Pacific Islander, 4.1% multiracial, 22.7% Hispanic. 21.8% of investigations were substantiated; 51.1% of investigations were for neglect, 12.2% for physical abuse, 4.4% for sexual abuse, 2.6% for emotional abuse, 15.6% for multiple maltreatment types, and 5.3% for other maltreatment types.<br><br>Out-of-home care placement: N = 3,619,387 child investigations; 50.1% female; mean age = 8.0 (SD 5.0) years; 48.8% White, 21.3% Black, 1.1% American Indian/Alaskan Native, 0.9% Asian, 0.2% Hawaiian/Pacific Islander, 4.2% multiracial, 23.4% Hispanic. 7.9% of investigations resulted in an out-of-home care placement; 47.2% of investigations were for neglect, 13.5% for physical abuse, 4.8% for sexual abuse, 2.8% for emotional abuse, 16.3% for multiple maltreatment types, and 5.8% for other maltreatment types.<br><br>(*N.B.* individual children could have multiple investigations) | types of abuse, other) and out-of-home care placement [Outcome] | 1.17), and Hispanic (adj. OR = 1.09, 95% CI 1.08, 1.09) children all had a greater likelihood of substantiation and Asian children had a lower likelihood (adj. OR = 0.95, 95% CI 1.03, 1.16). County-level factors that increased the odds of substantiation were higher percentage of single-headed households (standardised) (adj. OR = 1.04, 95% CI 1.03, 1.05) and rural locale (adj. OR = 1.10, 95% CI 1.08, 1.12). Factors that decreased odds of substantiation were high child poverty rate (adj. OR = 0.95, 95% CI 0.91, 0.98), greater percentage of Black residents (standardised) (adj. OR = 0.96, 95% CI 0.95, 0.98), and higher juvenile arrest rate (standardised) (adj. OR = 0.99, 95% CI 0.97, 1.00).<br><br>Compared with White/non-Hispanic children, Black (adj. OR = 1.15, 95% CI 1.14, 1.16), American Indian/Alaskan Native (adj. OR = 1.23, 95% CI 1.19, 1.28), multiracial (adj. OR = 1.43, 95% CI 1.40, 1.46) children all had a greater likelihood of out-of-home care placement and Asian (adj. OR = 0.66, 95% CI 0.63, 0.69) and Hispanic (adj. OR = 0.97, 95% CI 0.96. 0.99) children had a lower likelihood (no sig. difference for Hawaiian/Pacific Islander). A higher percentage of single-headed households (standardised) (adj. OR = 1.09, 95% CI 1.07, 1.11) was the only county-level factor that increased the odds of out-of-home care placement. Factors that decreased odds of out-of-home care placement were high child poverty rate (adj. OR = 0.90, 95% CI 0.85, 0.95), greater percentage of Black residents (standardised) (adj. OR = 0.90, 95% CI |

| | | | | | | |
|---|---|---|---|---|---|---|
| | | | | | | 0.89, 0.92), higher juvenile arrest rate (standardised) (adj. OR = 0.97, 95% CI 0.96, 0.99), and rural local (adj. OR = 0.97, 95% CI 0.94, 0.99).<br><br>Additional results/interaction effects can be found in the main paper. |
| Murphey (2000)<br><br>USA | **Purpose:** Descriptive & service evaluation (home visiting)<br><br>**Aims:** To test the feasibility of linking administrative datasets to provide broad information on programme effectiveness and client characteristics. To examine family characteristics, at the time of birth, of abuse and non-abused children to identify correlates of child maltreatment.<br><br>**Outcomes:** Programme effectiveness and client characteristics; factors associated with child maltreatment | 1. **Health** (vital statistics, Vermont Department of Health home visiting records)<br>2. No<br>3. Yes<br>3. Yes<br>4. 0-3 years | Retrospective, one-time linkage. Linkage technique (probabilistic vs. deterministic) ND. Linked on mother's first and last name, child's first and last name, child's date of birth, child's Social Security Number. 90.5% match rate for children receiving home visiting, 70.9% match rate for mothers receiving home visiting. Manual review of matched records showed 1.2% were matched incorrectly and 1.6% had minor inconsistences (97.2% maintained match status); manual review of unmatched records resulted in 1.9% matched through visual examination. | All births from 1992-1995 in Vermont, USA (N = 29,354; N = 9290 in home visiting programme, of whom N = 8403 were matched with birth records. N = 385 children (1.3%) experienced substantiated abuse/neglect during reporting period (N = 266 (3.1%) who received home visits and N = 119 (0.6%) who did not receive home visits). | Substantiated child abuse and neglect (could be by someone other than parents/caregivers) [Outcome] | Children receiving home visits were more likely than those not receiving home visits to have a substantiated maltreatment report (3.1% vs. 0.6%; OR = 2.4). The risk factors associated with increased likelihood of substantiated maltreatment were maternal education less than high school (adj. OR = 4.8), no prenatal care (adj. OR = 3.6), unmarried mother (adj. OR = 2.2), multiple birth (adj. OR = 2.1), maternal smoking, late prenatal care, and parity > 1 (precise ORs not given for final three factors; approx. ORs = 1.5) |
| **Multi-sectoral involvement** | | | | | | |

| | | | | | | |
|---|---|---|---|---|---|---|
| Aalders (2012)<br><br>Australia | **Purpose:** Descriptive<br><br>**Aims:** To examine links between child maltreatment, homelessness, and juvenile justice<br><br>**Outcomes:** Pathways between child protection, homelessness, and criminal justice | 1. **Justice** (Juvenile Justice National Minimum Data Set); **social services** (CPS records, Supported Accommodation Assistance Program)<br>2. No<br>3. Yes<br>4. No<br>5. ND | Retrospective, one-time linkage using deterministic linkage technique. Linked on a statistical linkage key comprised of 5 letters of name, date of birth, and sex). This method identified 99.7% of links using name-based linkage with clerical review ('true links'). Clients provided consent for use of their records. | ND | Substantiated child protection notifications [Exposure/ outcome, depending on analysis] | Young people involved in one of the three sectors (justice, housing support, child protection) were more likely than the general population to have involvement in ≥1 other sector. 6% of young people with a maltreatment substantiation had received housing support in the year preceding the substantiation and 9% received support in the year following the substantiation (4% and 6%, respectively, for child protection notifications more generally). 8% of young people with a child protection notification were also under juvenile justice supervision; these young people entered supervision earlier than their peers (21% entered between age 10-13, compared with 6% for those without notifications). |

| | | | | | | |
|---|---|---|---|---|---|---|
| Eastman (2019b)<br><br>USA | **Purpose:** Descriptive<br><br>**Aims:** To document the full child protective service (CPS) histories of arrested youth and young adults<br><br>**Outcomes:** Criminal justice contact before age 24 | 1. **Justice** (California Department of Justice's Automated Criminal History System (felonies and misdemeanours)); **social services** (CPS records)<br>2. No<br>3. Yes<br>4. No<br>5. 0-24 years | Retrospective, one-time linkage using probabilistic linkage technique. Linked on a combination of unique (social security number) and non-unique (first name, middle name, last name, date of birth) identifiers. Permission to access records agreed between university and data-holding organisations (no consent from individuals required). Linkage validation/quality assessment ND. | N = 394,377 individuals aged ≤24 arrested and booked for misdemeanours/felonies. 25.4% female; mean age at arrest = 20.5 years (SD 3.0); 50.1% Hispanic, 24.9% non-Hispanic White, 16.1% non-Hispanic Black, 8.9% non-Hispanic other/missing; 43.4% had a history of CPS involvement. | Maltreatment reports (neglect, physical abuse, sexual abuse). Four hierarchical groupings of maltreatment: (1) no history of CPS involvement, (2) 1+ unsubstantiated reports of maltreatment and no substantiated reports/foster care experience, (3) 1+ reports of substantiated maltreatment reports but no foster care experience, (4) foster care placement due to maltreatment. [Exposure] | 43.4% of all arrested youth had had CPS involvement (24.5% unsubstantiated reports, 9.6% substantiated reports with no foster care placement, 9.4% foster care placement due to maltreatment). 51.6% of youth with CPS involvement had reports of alleged physical abuse, 9.9% of sexual abuse, and 26.7% of multiple types of maltreatment. 59.6% had their first reports before age 10. Factors that were significantly associated with CPS involvement were female sex, non-Hispanic Black ethnicity, greater number of arrests, and felony charges. 60.2% of youth with uncensored data (birth in or after 1998) had contact vs. 41.3% with censored data (birth before 1998). 31.0% of youth born in or after 1998 had unsubstantiated reports, 14.1% had substantiated reports with no foster care placement, and 15.1% had foster care placement due to maltreatment (vs. 23.7%, 9.0%, and 8.7%, respectively, for those born before 1998). |

| | | | | | | |
|---|---|---|---|---|---|---|
| Herz (2019)<br><br>USA | **Purpose:** Descriptive<br><br>**Aims:** To determine incidence rates for dual system youth overall and by pathway to determine whether dual system youth contact differs in type and in timing. To test whether the characteristics and system experiences vary significantly (a) between dual system youth compared to their single system counterparts and (b) across dual system pathways.<br><br>**Outcomes:** Involvement in the child welfare and juvenile justice systems ('dual system') | 1. **Justice** (juvenile justice, police, court services (see paper for details on sources for each of the three study sites)); **social services** (CPS records)<br>2. No<br>3. Yes<br>4. No<br>5. 10-18 years | Retrospective, one-time linkage using probabilistic linkage technique. Linked on 'personally-identifying information'. Data were de-identified. Consent for analysis not required because of de-identified nature of data. Linkage validation/quality assessment ND. | N = 15,076 youth with a juvenile delinquency court petition and child welfare involvement and comparison group of N = 499,467 with child welfare involvement only in three locations (New York City and two counties in Illinois and Ohio). Dual system youth: 22.0-35.5% female across three counties; 71.3-79.4% African American. Child welfare only youth: 48.7-50.1%; 38.7-60.1% African American. | Child maltreatment reports for abuse, neglect (regardless of substantiation), and receipt of child welfare services [Outcome] | Dual system contact of any type among youth with a juvenile justice court petition ranged from 44.8-70.4% across the three sites. Dual system 'pathways' varied greatly: non-concurrent system contact was more prevalent than concurrent contact (49.2-72.6% vs. 27.4-46.5%). The most common pathway by far was the 'child welfare pathway', i.e. child welfare before juvenile justice (48.3-70.8%). Characteristics and system experiences varied within and between different pathways. Compared with youth with child welfare contact only, dual system youth had a significantly higher proportion of males, proportion of African American youth, average age at first and last welfare investigations, average number of investigations, length of time spent in child welfare, proportion ever placed in out-of-home care, and average number of placements. |

| | | | | | | |
|---|---|---|---|---|---|---|
| Hurren (2017)<br><br>Australia | **Purpose:** Descriptive<br><br>**Aims:** To examine the consistency of life-course child maltreatment trajectories and youth offending links across birth cohorts<br><br>**Outcomes:** Youth offending (binary presence/absence of ≥1 'guilty' verdict) | 1. Queensland Linkage Project (QLP): **justice** (juvenile justice); **social services** (CPS records)<br>2. No<br>3. Yes<br>4. No<br>5. 0-18 years | Retrospective, one-time linkage using probabilistic linkage technique. Data were shared with researchers in de-identified format. Linkage validation/quality assessment ND. | N = 4511 children born in 1990 with at least one substantiated maltreatment report. 54% female; 15% Indigenous. | CPS involvement (including, but not limited to physical abuse, emotional abuse, sexual abuse or exploitation, or neglect). Only one type of maltreatment could be recorded per report (the 'most serious' type); multi-type maltreatment therefore required change across multiple reports [Exposure] | 15.7% of children with ≥1 substantiated maltreatment report had received a guilty verdict by age 18. Six groups of maltreatment trajectories were identified: (1) 'adolescent peak - chronic victimisation (AP-CV)'; (2) 'adolescent limited - low victimisation (AL-LV)'; (3) 'primary school transition - chronic victimisation (PST-CV)'; (4) 'middle childhood limited - low victimisation (MCL-LV)'; (5) 'early childhood limited - low victimisation (ECL-LV)'; and (6) secondary school transition - chronic victimisation (SST-CV)' (see paper for additional details on trajectory characteristics). Proportion of each trajectory group with a guilty verdict ranged from 8.9 (ECL-LV) to 45.3% (AP-CV). Groups with chronic maltreatment (AP-CV, SST-CV, PST-CV) had the largest proportion of young offenders (30.3-45.3%). Results were largely consistent with findings from a previous study of the 1983-4 cohort. |
| Rodriguez (2016)<br><br>USA | **Purpose:** Descriptive<br><br>**Aims:** To examine the patterns and sequence of families' involvement with homeless shelters and CPS and whether involvement in each system predicts involvement in the other<br><br>**Outcomes:** CPS involvement, shelter system involvement | 1. **Social services** (Alameda County Social Services CPS records, public assistance data, Homeless Management Information System); **study-specific** (incl. a psychosocial challenges index)<br>2. Yes<br>3. No<br>4. Yes<br>5. ND | Could not categorise due to insufficient description of linkage techniques. Families gave consent at enrolment. Linkage validation/quality assessment ND. | N = 258 families in emergency shelters with at least one child aged 15 or younger. Surveyed parents: 95.7% female; mean age = 32.0 (SD 8.9); 57.0% Black, 19.0% Hispanic, 11.6% White, 4.7% Asian/Pacific Islander, 7.7% other. 57.8% of families had ≥1 referral for abuse/neglect, 48.5% had ≥1 investigated referral, and 18.6% had ≥1 substantiated referral. | CPS referrals for abuse/neglect, regardless of investigation or substantiation [Exposure/outcome, dependent on research questions] | 57.8% of families had ≥1 referral for abuse/neglect, 48.5% had ≥1 investigated referral, and 18.6% had ≥1 substantiated referral. Un-investigated/unfounded reports increased in the months before shelter entry and spiked immediately after entry; substantiated reports increased after shelter entry. Shelter use before study entry was associated with any subsequent CPS referrals (adj. OR = 2.84, 95%CI 1.30, 6.19) but not investigations or substantiations. Ethnicity was not associated with CPS referrals after study entry in multivariable models. Additional shelter episodes after study entry were not |

| | | | | | | |
|---|---|---|---|---|---|---|
| | | | | | | predicted by CPS referral. |
| **Methodological considerations and advancements** | | | | | | |

| | | | | | | |
|---|---|---|---|---|---|---|
| Parrish (2017)<br><br>USA | **Purpose:** Methodological advancement/quality assessment<br><br>**Aims:** To evaluate and quantify the impact of non-linkage misspecification and single source maltreatment ascertainment use on reported maltreatment risk and effect estimates in a linked dataset<br><br>**Outcomes:** Child maltreatment reports and deaths | 1. Alaska Longitudinal Child Abuse and Neglect Linkage (ALCANLink): **health** (Pregnancy Risk Assessment Monitoring System, vital records, Alaska Child Death Review, Maternal Child Death Review); **justice** (Anchorage Police Department); **social services** (CPS records, Child Advocacy Center agency reports); **other** (Alaska Permanent Fund Dividend)<br>2. Yes<br>3. Yes<br>4. Yes<br>5. 0-5 years | Multiple one-time linkages using deterministic and probabilistic (iterative) techniques. Linked on first, last, and alias names, date of birth and sex. Data were provided to researchers in de-identified format. Examination of match rates showed 94.1% of all PRAMS births could be linked to records from the Department of Revenue records (the most universal of the datasets). See Parrish (2017) for additional detail on quality assessment and match rates. | N = 1162 PRAMS-eligible births in Alaska, USA 2009 (further details not given). Weighted prevalence estimates for CPS reports among all PRAMS births were 2.7% (95% CI 1.7, 6.5) for sexual abuse allegations, 5.1% (95% CI 3.6, 6.5) for physical abuse allegations, 9.1% (95% CI 7.2, 11.0) for mental injury allegations, and 21.0% (95% CI 18.4, 23.6) for neglect allegations. | Child maltreatment reports (sexual abuse, physical abuse, mental injury, neglect) and maltreatment-related mortality [Outcome] | N = 327 (24.2%) children had ≥1 multi-source maltreatment report in the study period. Before age 1 was the most common time for reports (39.1%). Neglect was the most common reason for CPS reports (88.7% of all reports), followed by mental injury (38.5%), physical abuse (21.4%), sexual abuse (11.6%). CPS captured 98%, Child Advocacy Centre 13%, Anchorage Police Department 10%, Child Death Review N = 5 fatalities (2% of all maltreatment reports). Overall (weighted) incidence proportion estimate for the cohort of a multi-source report before age 6 was 28.3% (95% CI 23.6, 33.0); failure to account for out-of-state emigration biased this proportion by 12% (from 28.3% to 25.2%) and HRs by up to 33%. Restrictive linkage parameters (deterministic linkage matches only) further biased the incidence proportion and risk factor HRs downward. Multi-source linkages did not vastly improve estimate quality versus using CPS reports alone. |

| | | | | | | |
|---|---|---|---|---|---|---|
| Putnam-Hornstein (2011c)<br><br>USA | **Purpose:** Descriptive & methodological advancement (surveillance)<br><br>**Aims:** To provide a population-level view of children reported for maltreatment during the first five years of life in California<br><br>**Outcomes:** Maltreatment allegation before age 5 | 1. **Health** (vital birth records); **social services** (CPS records)<br>2. No<br>3. Yes<br>4. No<br>5. 0-5 years | Retrospective linkage using probabilistic linkage technique. Linked on child's first name, middle name, last name, date of birth, ethnicity, gender, maternal and paternal names and Social Security Numbers. Match rate was 84% with differences by substantiation (substantiated files more likely to be linked) but not type of allegation. | All live births in California 1999-2002 (N = 2,112,277), focusing on those who had a maltreatment report before age 5 (293,441; 13.9%). 7.4% had an allegation of neglect, 2.5% of risk/other, 1.8% of physical abuse, 1.5% emotional abuse, 0.7% sexual abuse. | Allegations of maltreatment (incl. physical abuse, sexual abuse, emotional abuse, neglect, and risk of maltreatment/other), regardless of investigation or substantiation [Outcome] | 13.9% of children were reported for possible maltreatment by age 5. (7.4% had an allegation of neglect, 2.5% of risk/other, 1.8% of physical abuse, 1.5% emotional abuse, 0.7% sexual abuse); 5.2% had a substantiated report. Higher rates of reports were observed for children with a health risk at birth (17.9% had allegations vs. 13.4% of children without health risk), birth payment with public insurance (21.6% vs. 8.5% of those with other types of payment), children of Black mothers (29.7% vs. 5.8-14.1% for other ethnicities), children with missing paternity on their birth records (33.7% vs. 11.8% with established paternity), and children second or higher in the birth order (16.0% vs. 10.6% of first-borns). Dose response relationships were also seen for maternal age (higher rates for younger ages) and education (higher rates for less education). |

| | | | | | | |
|---|---|---|---|---|---|---|
| Raghavan (2015)<br><br>USA | **Purpose:** Methodological advancement/quality assessment<br><br>**Aims:** To assess the validity of Medicaid (ICD-9) codes in identifying maltreatment<br><br>**Outcomes:** Maltreatment (agreement between caseworker ascertainment and ICD-9 codes) | 1. **Health** (Medicaid Claims Files); **study-specific** (National Survey of Child and Adolescent Wellbeing (longitudinal study of children with CPS contact))<br>2. Yes<br>3. No<br>4. No<br>5. ND | Retrospective, one-time linkage using deterministic linkage technique. Linked on child's Social Security Number. Consent for linkage sought from young people and caregivers. Linkage validation/quality assessment ND. | N = 2136 youth from the National Survey of Child and Adolescent Wellbeing (youth with a history of CPS involvement) | Maltreatment (physical abuse, sexual abuse, neglect, abandonment) as determined by caseworkers (gold standard) and ICD-9 codes [Outcome] | 15.2% of children identified by caseworkers as having a history of maltreatment had an ICD-9 code in their Medicaid files (over 4 years). Rates were similar across types of maltreatment (~15%) though sexual abuse was coded in Medicaid records at a slightly higher rate (24.6% had an ICD-9 code). Of children identified by caseworkers, boys were less likely than girls to have an ICD-9 code (OR = 0.7, SE 0.1, p<0.01); children aged 3-5 (OR = 2.1, SE 0.5, p<0.01) and 6-11 years (OR = 2.1 ,SE 0.4, p<0.05) were more likely than those aged 0-2 to have an ICD-9 code; African American children were less likely than White children to have an ICD-9(OR = 0.6, SE 0.1, p<0.05); children in out-of-home care were more likely than those in in-home care to have an ICD-9 code (OR = 2.0, SE 0.3, p<0.001); and those with any hospital (OR = 1.9, SE 0.4, p<0.01) or emergency department visit (OR = 1.4, SE 0.3, p<0.05) were more likely than those with none to have an ICD-9 code. Over-ascertainment (ICD-9 code but no caseworker determination) was more common for children resident in rural communities compared with urban ones (OR = 3.9, 85% CI 1.4, 10.5) and children in out-of-home care rather than in-home care (OR = 2.4, 95% CI 1.01, 5.8). |

| | | | | | | |
|---|---|---|---|---|---|---|
| Schnitzer (2008)<br><br>USA | **Purpose:** Descriptive & methodological advancement (surveillance)<br><br>**Aims:** To describe approaches to surveillance of fatal child maltreatment and to identify options for improving case ascertainment<br><br>**Outcomes:** Maltreatment-related deaths | 1. **Health** (death review data, death certificates, medical examiner records (Rhode Island only)); **justice** (homicide reports made to the FBI Uniform Crime Report system); **social services** (child welfare records, Child Abuse Central Index (California only)).<br>2. No<br>3. Yes<br>4. No<br>5. 0-10 years | Retrospective, one-time linkage. Linkage technique (probabilistic vs. deterministic) ND. Linkage validation/quality assessment ND. | All children 0-10 years old in California, Michigan, and Rhode Island, USA. Among N = 510 children who died due to maltreatment: 48.2% female; 47.6% White, 29.8% African American, 19.6% Hispanic, 3.1% Asian/Pacific Islander, 0.8% American Indian/Alaska Native, 18.6% other/unknown. | Child maltreatment fatalities [Outcome] | There were 258 maltreatment deaths in California (2.5 per 100,000 (95% CI 2.1, 2.9)), 192 in Michigan (6.8 per 100,000 (95% CI 5.4, 8.2)), and 60 in Rhode Island (8.8 per 100,000 (95% CI 3.7, 13.9)). Most deaths in California were identified by the FBI Uniform Crime Reporting system (56%) or child death review team (55%); in Rhode Island by the child death review team (98%); in Michigan by the child welfare agency (44%). Death certificates identified <20% of deaths in all three states; the FBI system identified <20% in Michigan and Rhode Island. 90% of the deaths could be identified by linking only 2/4 sources in each state (though sources differed by state). |

| | | | | | | |
|---|---|---|---|---|---|---|
| Scott (2012)<br><br>New Zealand | **Purpose:** Descriptive & methodological advancement/quality assessment<br><br>**Aims:** To investigate associations of psychopathology with prospective versus retrospective maltreatment ascertainment<br><br>**Outcomes:** Mental health disorders (major depression, anxiety disorders, drug use disorder, and alcohol use disorder) | 1. **Social services** (Child, Youth and Family agency (CPS) records); **study-specific** (New Zealand Mental Health Survey)<br>2. Yes<br>3. No<br>4. No<br>5. Maltreatment ascertained in CPS records from 0-17 years (retrospective recall as an adult, aged 16-27) | Retrospective, one-time linkage. Linkage technique (probabilistic vs. deterministic) ND (linked on name, date of birth, and survey number). Written informed consent obtained for linkage. 5% of survey sample was not linked due to missing name (but missing at random). | N = 2144 respondents to the New Zealand Mental Health Survey (aged 16-27). 10.3% had prospectively-ascertained maltreatment (i.e. documented in CPS data); 16.7% had retrospectively-ascertained maltreatment only (i.e. recalled in survey but did not have documented maltreatment in CPS data). | *As measured in survey:* physical abuse, child rape/sexual abuse, witnessing intimate partner violence; *as measured in CPS files:* screened-in reports of maltreatment [Exposure] | 10.3% of survey respondents had prospectively-ascertained maltreatment (i.e. documented in CPS data, regardless of recall); 16.7% had retrospectively-ascertained maltreatment (i.e. recalled in survey but did not have documented maltreatment in CPS data). Compared with those without a history of maltreatment, those with prospectively- or retrospectively-ascertained maltreatment had an increased likelihood of 12-month and lifetime major depressive disorder (adj. ORs = 2.37-2.51), anxiety disorders (adj. ORs = 2.05-3.10), and drug abuse/dependence (adj. ORs = 3.25-4.12) and lifetime (but not 12-month) alcohol abuse/dependence (adj. ORs = 2.98-3.36). There was no difference in strength of association between the prospectively- and retrospectively-ascertained maltreatment groups. Those with prospectively-ascertained maltreatment had unfavourable depression courses including early onset (median age = 14 years, IQR 12-17), number of lifetime episodes (median = 3.5, IQR 1.5-10), and impairment. |

## References


Aalders, R. & Australian Institute of Health and Welfare. (2012). *Children and young people at risk of social exclusion: Links between homelessness, child protection and juvenile justice*. Australian Institute of Health and Welfare. http://www.aihw.gov.au/publication-detail/?id=60129542237

Abajobir, A. A., Kisely, S., Williams, G., Strathearn, L., & Najman, J. M. (2017b). Childhood maltreatment and high dietary fat intake behaviors in adulthood: A birth cohort study. *Child Abuse & Neglect*, *72*, 147–153. https://doi.org/10.1016/j.chiabu.2017.08.002

Abajobir, A. A., Kisely, S., Williams, G., Strathearn, L., Clavarino, A., & Najman, J. M. (2017a). Does substantiated childhood maltreatment lead to poor quality of life in young adulthood? Evidence from an Australian birth cohort study. *Quality of Life Research*, *26*(7), 1697–1702. https://doi.org/10.1007/s11136-017-1517-5

Austin, A. E., Parrish, J. W., & Shanahan, M. E. (2018). Using time-to-event analysis to identify preconception and prenatal predictors of child protective services contact. *Child Abuse & Neglect*, *82*, 83–91. https://doi.org/10.1016/j.chiabu.2018.05.025

Boyd, M., Kisely, S., Najman, J., & Mills, R. (2019). Child maltreatment and attentional problems: A longitudinal birth cohort study. *Child Abuse & Neglect*, *98*, 104170. https://doi.org/10.1016/j.chiabu.2019.104170

Bruns, E. J., Pullmann, M. D., Weathers, E. S., Wirschem, M. L., & Murphy, J. K. (2012). Effects of a Multidisciplinary Family Treatment Drug Court on Child and Family Outcomes: Results of a Quasi-Experimental Study. *Child Maltreatment*, *17*(3), 218–230. https://doi.org/10.1177/1077559512454216

Cant, R. L., O'Donnell, M., Sims, S., & Harries, M. (2019). Overcrowded housing: One of a constellation of vulnerabilities for child sexual abuse. *Child Abuse & Neglect*, *93*, 239–248. https://doi.org/10.1016/j.chiabu.2019.05.010

Cederbaum, J. A., Putnam-Hornstein, E., King, B., Gilbert, K., & Needell, B. (2013). Infant Birth Weight and Maltreatment of Adolescent Mothers. *American Journal of Preventive Medicine*, *45*(2), 197–201. https://doi.org/10.1016/j.amepre.2013.03.016

Coulton, C. J., Richter, F., Kim, S.-J., Fischer, R., & Cho, Y. (2016). Temporal effects of distressed housing on early childhood risk factors and kindergarten readiness. *Children and Youth Services Review*, *68*, 59–72. https://doi.org/10.1016/j.childyouth.2016.06.017

Cram, F., Gulliver, P., Ota, R., & Wilson, M. (2015). Understanding Overrepresentation of Indigenous Children in Child Welfare Data: An Application of the Drake Risk and Bias Models. *Child Maltreatment*, *20*(3), 170–182. https://doi.org/10.1177/1077559515580392

Cutajar, M. C., Mullen, P. E., Ogloff, J. R. P., Thomas, S. D., Wells, D. L., & Spataro, J. (2010a). Psychopathology in a large cohort of sexually abused children followed up to 43 years. *Child Abuse & Neglect*, *34*(11), 813–822. https://doi.org/10.1016/j.chiabu.2010.04.004

Cutajar, M. C., Mullen, P. E., Ogloff, J. R. P., Thomas, S. D., Wells, D. L., & Spataro, J. (2010b). Schizophrenia and Other Psychotic Disorders in a Cohort of Sexually Abused Children. *Archives of General Psychiatry*, *67*(11), 1114–1119. https://doi.org/10.1001/archgenpsychiatry.2010.147

Dahl, S. K., Larsen, J. T., Petersen, L., Ubbesen, M. B., Mortensen, P. B., Munk-Olsen, T., & Musliner, K. L. (2017). Early adversity and risk for moderate to severe unipolar depressive disorder in adolescence and adulthood: A register-based study of 978,647 individuals. *Journal of Affective Disorders*, *214*, 122–129. https://doi.org/10.1016/j.jad.2017.03.014

Eastman, A. L., & Putnam-Hornstein, E. (2019a). An examination of child protective service involvement among children born to mothers in foster care. *Child Abuse & Neglect*, *88*, 317–325. https://doi.org/10.1016/j.chiabu.2018.11.002

Eastman, A. L., Foust, R., Prindle, J., Palmer, L., Erlich, J., Giannella, E., & Putnam-Hornstein, E. (2019b). A Descriptive Analysis of the Child Protection Histories of Youth and Young Adults Arrested in California. *Child Maltreatment*, *24*(3), 324–329. https://doi.org/10.1177/1077559519837667

Eastman, A. L., Mitchell, M. N., & Putnam-Hornstein, E. (2016). Risk of re-report: A latent class analysis of infants reported for maltreatment. *Child Abuse & Neglect*, *55*, 22–31. https://doi.org/10.1016/j.chiabu.2016.03.002

Ekéus, C., Christensson, K., & Hjern, A. (2004). Unintentional and violent injuries among pre-school children of teenage mothers in Sweden: A national cohort study. *Journal of Epidemiology & Community Health*, *58*(8), 680–685. https://doi.org/10.1136/jech.2003.015255

Finno-Velasquez, M., Palmer, L., Prindle, J., Tam, C. C., & Putnam-Hornstein, E. (2017). A birth cohort study of Asian and Pacific Islander children reported for abuse or neglect by maternal nativity and ethnic origin. *Child Abuse & Neglect*, *72*, 54–65. https://doi.org/10.1016/j.chiabu.2017.07.009

Fisher, M. H., Epstein, R. A., Urbano, R. C., Vehorn, A., Cull, M. J., & Warren, Z. (2019). A population-based examination of maltreatment referrals and substantiation for children with autism spectrum disorder. *Autism: The International Journal of Research and Practice*, *23*(5), 1335–1340. https://doi.org/10.1177/1362361318813998

Font, S. A., Cancian, M., & Berger, L. M. (2019). Prevalence and Risk Factors for Early Motherhood Among Low-Income, Maltreated, and Foster Youth. *Demography*, *56*(1), 261–284. https://doi.org/10.1007/s13524-018-0744-x

Font, S. A., & Maguire-Jack, K. (2020a). It's not "Just poverty": Educational, social, and economic functioning among young adults exposed to childhood neglect, abuse, and poverty. *Child abuse & neglect*, *101*, 104356.

Font, S., Cancian, M., Berger, L. M., & DiGiovanni, A. (2020b). Patterns of intergenerational child protective services involvement. *Child abuse & neglect*, *99*, 104247.

Galos, D. L. (2017). An Epidemiologic Analysis of Intergenerational Child Maltreatment [Doctoral dissertation]. Minnesota, USA; University of Minnesota.

Gessner, B. D., Moore, M., Hamilton, B., & Muth, P. T. (2004). The incidence of infant physical abuse in Alaska. *Child Abuse & Neglect*, *28*(1), 9–23. https://doi.org/10.1016/j.chiabu.2003.03.002

Gilbert, R., Fluke, J., O'Donnell, M., Gonzalez-Izquierdo, A., Brownell, M., Gulliver, P., Janson, S., & Sidebotham, P. (2012). Child maltreatment: Variation in trends and policies in six developed countries. *The Lancet*, *379*(9817), 758–772. https://doi.org/10.1016/S0140-6736(11)61087-8

Green, B. L., Sanders, M. B., & Tarte, J. (2017). Using administrative data to evaluate the effectiveness of the Healthy Families Oregon home visiting program: 2-year impacts on child maltreatment & service utilization. *Children and Youth Services Review*, *75*, 77–86. https://doi.org/10.1016/j.childyouth.2017.02.019

Green, M. J., Kariuki, M., Chilvers, M., Butler, M., Katz, I., Burke, S., Tzoumakis, S., Laurens, K. R., Harris, F., & Carr, V. J. (2019b). Inter-agency indicators of out-of-home-care placement by

age 13–14 years: A population record linkage study. *Child Abuse & Neglect*, *93*, 91–102. https://doi.org/10.1016/j.chiabu.2019.04.013

Green, M. J., Tzoumakis, S., Laurens, K. R., Dean, K., Kariuki, M., Harris, F., Brinkman, S. A., & Carr, V. J. (2019a). Early developmental risk for subsequent childhood mental disorders in an Australian population cohort. *Australian & New Zealand Journal of Psychiatry*, *53*(4), 304–315. https://doi.org/10.1177/0004867418814943

Green, M. J., Tzoumakis, S., Laurens, K. R., Dean, K., Kariuki, M., Harris, F., O'Reilly, N., Chilvers, M., Brinkman, S. A., & Carr, V. J. (2018a). Latent profiles of early developmental vulnerabilities in a New South Wales child population at age 5 years. *Australian & New Zealand Journal of Psychiatry*, *52*(6), 530–541. https://doi.org/10.1177/0004867417740208

Green, M. J., Tzoumakis, S., McIntyre, B., Kariuki, M., Laurens, K. R., Dean, K., Chilvers, M., Harris, F., Butler, M., Brinkman, S. A., & Carr, V. J. (2018b). Childhood Maltreatment and Early Developmental Vulnerabilities at Age 5 Years. *Child Development*, *89*(5), 1599–1612. https://doi.org/10.1111/cdev.12928

Gwaltney, A. Y. (2018) Use of psychotropic medication in foster care and related outcomes [Doctoral dissertation]. North Carolina, USA. University of North Carolina at Chapel Hill.

Hafekost, K., Lawrence, D., O'Leary, C., Bower, C., O'Donnell, M., Semmens, J., & Zubrick, S. R. (2017a). Maternal alcohol use disorder and subsequent child protection contact: A record-linkage population cohort study. *Child Abuse & Neglect*, *72*, 206–214. https://doi.org/10.1016/j.chiabu.2017.08.010

Hafekost, K., Lawrence, D., O'Leary, C., Bower, C., Semmens, J., & Zubrick, S. R. (2017b). Maternal Alcohol Use Disorder and Risk of Child Contact with the Justice System in Western Australia: A Population Cohort Record Linkage Study. *Alcoholism: Clinical and Experimental Research*, *41*(8), 1452–1460. https://doi.org/10.1111/acer.13426

Herz, D. C., Dierkhising, C. B., Raithel, J., Schretzman, M., Guiltinan, S., Goerge, R. M., Cho, Y., Coulton, C., & Abbott, S. (2019). Dual System Youth and their Pathways: A Comparison of Incidence, Characteristics and System Experiences using Linked Administrative Data. *Journal of Youth and Adolescence*, *48*(12), 2432–2450. https://doi.org/10.1007/s10964-019-01090-3

Högberg, U., Andersson, J., Squier, W., Högberg, G., Fellman, V., Thiblin, I., & Wester, K. (2018). Epidemiology of subdural haemorrhage during infancy: A population-based register study. *PLOS ONE*, *13*(10), e0206340. https://doi.org/10.1371/journal.pone.0206340

Högberg, U., Lampa, E., Högberg, G., Aspelin, P., Serenius, F., & Thiblin, I. (2018a). Infant abuse diagnosis associated with abusive head trauma criteria: Incidence increase due to overdiagnosis? *European Journal of Public Health*, *28*(4), 641–646. https://doi.org/10.1093/eurpub/cky062

Högberg, U., Sennerstam, R., Wester, K., Högberg, G., Andersson, J., & Thiblin, I. (2019). Medical diagnoses among infants at entry in out-of-home care: A Swedish population-register study. *Health Science Reports*, *2*(8), e133. https://doi.org/10.1002/hsr2.133

Hong, S., & Piescher, K. (2012). The role of supportive housing in homeless children's well-being: An investigation of child welfare and educational outcomes. *Children and Youth Services Review*, *34*(8), 1440–1447. https://doi.org/10.1016/j.childyouth.2012.03.025

Hook, J. L., Romich, J. L., Lee, J. S., Marcenko, M. O., & Kang, J. Y. (2016). Trajectories of Economic Disconnection among Families in the Child Welfare System. *Social Problems*, *63*(2), 161–179. https://doi.org/10.1093/socpro/spw006

Horn, T. L., Piescher, K., Shannon, P. J., Hong, S., & Benton, A. (2017). Experiences of Somali and Oromo youth in the child protection system. *Children and Youth Services Review*, *79*, 10–19. https://doi.org/10.1016/j.childyouth.2017.05.022

Hu, N., Taylor, C. L., Li, J., & Glauert, R. A. (2017). The impact of child maltreatment on the risk of deliberate self-harm among adolescents: A population-wide cohort study using linked administrative records. *Child Abuse & Neglect*, *67*, 322–337. https://doi.org/10.1016/j.chiabu.2017.03.012

Hurren, E., Stewart, A., & Dennison, S. (2017). Transitions and turning points revisited: A replication to explore child maltreatment and youth offending links within and across Australian cohorts. *Child Abuse & Neglect*, *65*, 24–36. https://doi.org/10.1016/j.chiabu.2017.01.002

Jackisch, J., Brännström, L., & Almquist, Y. B. (2019). Troubled childhoods cast long shadows: Childhood adversity and premature all-cause mortality in a Swedish cohort. *SSM - Population Health*, *9*, 100506. https://doi.org/10.1016/j.ssmph.2019.100506

Jenkins, A. (2011) Early Engagement of Parents Involved in Child Welfare [Doctoral dissertation]. California, USA; Loma Linda University.

Johnson-Motoyama, M., Putnam-Hornstein, E., Dettlaff, A. J., Zhao, K., Finno-Velasquez, M., & Needell, B. (2015). Disparities in Reported and Substantiated Infant Maltreatment by Maternal Hispanic Origin and Nativity: A Birth Cohort Study. *Maternal and Child Health Journal*, *19*(5), 958–968. https://doi.org/10.1007/s10995-014-1594-9

Kalland, M., Sinkkonen, J., Gissler, M., Meriläinen, J., & Siimes, M. A. (2006). Maternal smoking behavior, background and neonatal health in Finnish children subsequently placed in foster care. *Child Abuse & Neglect*, *30*(9), 1037–1047. https://doi.org/10.1016/j.chiabu.2006.03.005

King, B. (2017). First Births to Maltreated Adolescent Girls: Differences Associated With Spending Time in Foster Care. *Child Maltreatment*, *22*(2), 145–157. https://doi.org/10.1177/1077559517701856

King, B., Lawson, J., & Putnam-Hornstein, E. (2013). Examining the Evidence: Reporter Identity, Allegation Type, and Sociodemographic Characteristics as Predictors of Maltreatment Substantiation. *Child Maltreatment*, *18*(4), 232–244. https://doi.org/10.1177/1077559513508001

King, B., Putnam-Hornstein, E., Cederbaum, J. A., & Needell, B. (2014). A cross-sectional examination of birth rates among adolescent girls in foster care. *Children and Youth Services Review*, *36*, 179–186. https://doi.org/10.1016/j.childyouth.2013.11.007

Kisely, S., Abajobir, A. A., Mills, R., Strathearn, L., Clavarino, A., Gartner, C., & Najman, J. M. (2020a). Child Maltreatment and Persistent Smoking From Adolescence Into Adulthood: A Birth Cohort Study. *Nicotine & Tobacco Research*, *22*(1), 66–73. https://doi.org/10.1093/ntr/ntz039

Kisely, S., Abajobir, A. A., Mills, R., Strathearn, L., Clavarino, A., & Najman, J. M. (2018). Child maltreatment and mental health problems in adulthood: Birth cohort study. *The British Journal of Psychiatry*, *213*(6), 698–703. https://doi.org/10.1192/bjp.2018.207

Kisely, S., Mills, R., Strathearn, L., & Najman, J. M. (2020b). Does child maltreatment predict alcohol use disorders in young adulthood? A cohort study of linked notifications and survey data. *Addiction*, *115*(1), 61–68. https://doi.org/10.1111/add.14794

Kjelsberg, E. (1999a). A long-term follow-up study of adolescent psychiatric in-patients. Part IV. Predictors of a non-negative outcome. *Acta Psychiatrica Scandinavica*, *99*(4), 247–251. https://doi.org/10.1111/j.1600-0447.1999.tb07220.x

Kjelsberg, E., & Dahl, A. A. (1999b). A long-term follow-up study of adolescent psychiatric in-patients. Part II. Predictors of delinquency. *Acta Psychiatrica Scandinavica*, *99*(4), 237–242. https://doi.org/10.1111/j.1600-0447.1999.tb07218.x

Kjelsberg, E., Sandvik, L., & Dahl, A. A. (1999c). A long-term follow-up study of adolescent psychiatric in-patients. Part I. Predictors of early death. *Acta Psychiatrica Scandinavica*, *99*(4), 231–236. https://doi.org/10.1111/j.1600-0447.1999.tb07217.x

Lanier, P., & Jonson-Reid, M. (2014a). Comparing Primiparous and Multiparous Mothers in a Nurse Home Visiting Prevention Program. *Birth*, *41*(4), 344–352. https://doi.org/10.1111/birt.12120

Lanier, P., & Rose, R. A. (2017). Admission to Psychiatric Residential Treatment Facilities among Youth from Families Investigated for Maltreatment. *Journal of Child and Family Studies*, *26*(1), 306–316. https://doi.org/10.1007/s10826-016-0541-3

Lanier, P., Jonson-Reid, M., Stahlschmidt, M. J., Drake, B., & Constantino, J. (2010). Child Maltreatment and Pediatric Health Outcomes: A Longitudinal Study of Low-income Children. *Journal of Pediatric Psychology*, *35*(5), 511–522. https://doi.org/10.1093/jpepsy/jsp086

Lanier, P., Kohl, P. L., Benz, J., Swinger, D., & Drake, B. (2014b). Preventing Maltreatment with a Community-Based Implementation of Parent–Child Interaction Therapy. *Journal of Child and Family Studies*, *23*(2), 449–460. https://doi.org/10.1007/s10826-012-9708-8

Larson, A. M. (2010). Cross system comparisons of children in treatment foster care: Using agency data to study cross-system child outcomes. *Children and Youth Services Review*, *32*(1), 89–97. https://doi.org/10.1016/j.childyouth.2009.07.014

Laurens, K. R., Islam, F., Kariuki, M., Harris, F., Chilvers, M., Butler, M., Schofield, J., Essery, C., Brinkman, S. A., Carr, V. J., & Green, M. J. (2020). Reading and numeracy attainment of children reported to child protection services: A population record linkage study controlling for other adversities. *Child Abuse & Neglect*, *101*, 104326. https://doi.org/10.1016/j.chiabu.2019.104326

Lee, J. S., Romich, J. L., Kang, J. Y., Hook, J. L., & Marcenko, M. O. (2017). The impact of income on reunification among families with children in out-of-home care. *Children and Youth Services Review*, *72*(C), 91–99.

Lee, M. Y., & Jonson-Reid, M. (2009). Needs and outcomes for low income youth in special education: Variations by emotional disturbance diagnosis and child welfare contact. *Children and Youth Services Review*, *31*(7), 722–731. https://doi.org/10.1016/j.childyouth.2009.01.005

Leslie, L. K., Landsverk, J., Ezzet-Lofstrom, R., Tschann, J. M., Slymen, D. J., & Garland, A. F. (2000). Children in foster care: Factors influencing outpatient mental health service use. *Child Abuse & Neglect*, *24*(4), 465–476. https://doi.org/10.1016/S0145-2134(00)00116-2

Lewis, T. L., Kotch, J., Wiley, T. R. A., Litrownik, A. J., English, D. J., Thompson, R., Zolotor, A. J., Block, S. D., & Dubowitz, H. (2011). Internalizing Problems: A Potential Pathway From Childhood Maltreatment to Adolescent Smoking. *Journal of Adolescent Health*, *48*(3), 247–252. https://doi.org/10.1016/j.jadohealth.2010.07.004

Maclean, M. J., Sims, S., Bower, C., Leonard, H., Stanley, F. J., & O'Donnell, M. (2017a). Maltreatment Risk Among Children With Disabilities. *Pediatrics*, *139*(4). https://doi.org/10.1542/peds.2016-1817

Maclean, M. J., Taylor, C. L., & O'Donnell, M. (2016). Pre-existing adversity, level of child protection involvement, and school attendance predict educational outcomes in a longitudinal study. *Child Abuse & Neglect*, *51*, 120–131. https://doi.org/10.1016/j.chiabu.2015.10.026

Maclean, M. J., Taylor, C. L., & O'Donnell, M. (2017b). Relationship between out-of-home care placement history characteristics and educational achievement: A population level linked

data study. *Child Abuse & Neglect*, *70*, 146–159. https://doi.org/10.1016/j.chiabu.2017.05.013

Maclean, M. J., Taylor, C. L., & O'Donnell, M. (2018). Out-of-Home Care and the Educational Achievement, Attendance, and Suspensions of Maltreated Children: A Propensity-Matched Study. *The Journal of Pediatrics*, *198*, 287-293.e2. https://doi.org/10.1016/j.jpeds.2018.03.027

Maguire-Jack, K., Font, S. A., & Dillard, R. (2019). Child protective services decision-making: The role of children's race and county factors. *American journal of orthopsychiatry*, *90*(1), 48.

Maloney, T., Jiang, N., Putnam-Hornstein, E., Dalton, E., & Vaithianathan, R. (2017). Black–White Differences in Child Maltreatment Reports and Foster Care Placements: A Statistical Decomposition Using Linked Administrative Data. *Maternal and Child Health Journal*, *21*(3), 414–420. https://doi.org/10.1007/s10995-016-2242-3

Matheson, S. L., Kariuki, M., Green, M. J., Dean, K., Harris, F., Tzoumakis, S., Tarren-Sweeney, M., Brinkman, S., Chilvers, M., Sprague, T., Carr, V. J., & Laurens, K. R. (2017). Effects of maltreatment and parental schizophrenia spectrum disorders on early childhood social-emotional functioning: A population record linkage study. *Epidemiology and Psychiatric Sciences*, *26*(6), 612–623. https://doi.org/10.1017/S204579601600055X

McDonnell, C. G., Boan, A. D., Bradley, C. C., Seay, K. D., Charles, J. M., & Carpenter, L. A. (2019). Child maltreatment in autism spectrum disorder and intellectual disability: Results from a population-based sample. *Journal of Child Psychology and Psychiatry*, *60*(5), 576–584. https://doi.org/10.1111/jcpp.12993

Meuleners, L. B., Lee, A. H., & Hendrie, D. (2010). Interpersonal violence hospitalisations for adolescents: A population-based study. *Journal of Paediatrics and Child Health*, *46*(11), 686–690. https://doi.org/10.1111/j.1440-1754.2010.01818.x

Morgan, V. A., Di Prinzio, P., Valuri, G., Croft, M., McNeil, T., & Jablensky, A. (2019). Are familial liability for schizophrenia and obstetric complications independently associated with risk of psychotic illness, after adjusting for other environmental stressors in childhood? *Australian & New Zealand Journal of Psychiatry*, *53*(11), 1105–1115. https://doi.org/10.1177/0004867419864427

Murphey, D. A., & Braner, M. (2000). Linking child maltreatment retrospectively to birth and home visit records: An initial examination. *Child Welfare*, *79*(6), 711–728.

Murphy, S., Shevlin, M., Elklit, A., McElroy, E., Murphy, J., Hyland, P., & Christoffersen, M. (2018). Validating Childhood Maltreatment Typologies Using Data Linkage. *Journal of Loss and Trauma*, *23*(2), 99–112. https://doi.org/10.1080/15325024.2017.1404204

Needell, B., Cuccaro-Alamin, S., Brookhart, A., Jackman, W., & Shlonsky, A. (2002). *Youth Emancipating from Foster Care in California: Findings Using Linked Administrative Data*. Center for Social Services Research, University of California at Berkeley, School of Social Welfare, 120 Haviland Hall, Berkeley, CA 94720-7400. https://eric.ed.gov/?id=ED477793

O'Donnell, M., Maclean, M. J., Sims, S., Morgan, V. A., Leonard, H., & Stanley, F. J. (2015). Maternal mental health and risk of child protection involvement: Mental health diagnoses associated with increased risk. *J Epidemiol Community Health*, *69*(12), 1175–1183. https://doi.org/10.1136/jech-2014-205240

O'Donnell, M., Nassar, N., Jacoby, P., & Stanley, F. (2012). Western Australian emergency department presentations related to child maltreatment and intentional injury: Population level study utilising linked health and child protection data. *Journal of Paediatrics and Child Health*, *48*(1), 57–65. https://doi.org/10.1111/j.1440-1754.2011.02189.x

O'Donnell, M., Nassar, N., Leonard, H. M., Mathews, R. P., Patterson, Y. G., & Stanley, F. J. (2010a). The use of cross-jurisdictional population data to investigate health indicators of

child maltreatment. *Medical Journal of Australia*, *193*(3), 142–145. https://doi.org/10.5694/j.1326-5377.2010.tb03832.x

O'Donnell, M., Nassar, N., Leonard, H., Jacoby, P., Mathews, R., Patterson, Y., & Stanley, F. (2010b). Rates and types of hospitalisations for children who have subsequent contact with the child protection system: A population based case-control study. *Journal of Epidemiology and Community Health*, *64*(9), 784–788. https://doi.org/10.1136/jech.2009.093393

Orr, C., Fisher, C., Sims, S., Preen, D., Glauert, R., & O'Donnell, M. (2019). Hospitalisations for maternal assault are associated with increased risk of child protection involvement. *Child Abuse & Neglect*, *95*, 104014. https://doi.org/10.1016/j.chiabu.2019.05.007

Papalia, N. L., Luebbers, S., Ogloff, J. R. P., Cutajar, M., Mullen, P. E., & Mann, E. (2017). Further victimization of child sexual abuse victims: A latent class typology of re-victimization trajectories. *Child Abuse & Neglect*, *66*, 112–129. https://doi.org/10.1016/j.chiabu.2017.02.040

Parrish, J. W., & Gessner, B. D. (2010). Infant Maltreatment-Related Mortality in Alaska: Correcting the Count and Using Birth Certificates to Predict Mortality. *Child Abuse & Neglect: The International Journal*, *34*(12), 951–958. https://doi.org/10.1016/j.chiabu.2010.07.002

Parrish, J. W., Fleckman, J. M., Prindle, J. J., Eastman, A. L., & Weil, L. E. G. (2020). Measuring the Incidence of Child Maltreatment Using Linked Data: A Two-State Comparison. *American Journal of Preventive Medicine*, *58*(4), e133–e140. https://doi.org/10.1016/j.amepre.2019.11.007

Parrish, J. W., Lanier, P., Newby-Kew, A., Arvidson, J., & Shanahan, M. (2016). Maternal Intimate Partner Violence Victimization Before and During Pregnancy and Postbirth Child Welfare

Contact: A Population-Based Assessment. *Child Maltreatment*, *21*(1), 26–36. https://doi.org/10.1177/1077559515616704

Parrish, J. W., Shanahan, M. E., Schnitzer, P. G., Lanier, P., Daniels, J. L., & Marshall, S. W. (2017). Quantifying sources of bias in longitudinal data linkage studies of child abuse and neglect: Measuring impact of outcome specification, linkage error, and partial cohort follow-up. *Injury Epidemiology*, *4*(1), 23. https://doi.org/10.1186/s40621-017-0119-6

Parrish, J. W., Young, M. B., Perham-Hester, K. A., & Gessner, B. D. (2011). Identifying Risk Factors for Child Maltreatment in Alaska: A Population-Based Approach. *American Journal of Preventive Medicine*, *40*(6), 666–673. https://doi.org/10.1016/j.amepre.2011.02.022

Patton, D. A., Liu, Q., Adelson, J. D., & Lucenko, B. A. (2019). Assessing the social determinants of health care costs for Medicaid-enrolled adolescents in Washington State using administrative data. *Health Services Research*, *54*(1), 52–63. https://doi.org/10.1111/1475-6773.13052

Prince, D. M., Vidal, S., Okpych, N., & Connell, C. M. (2019). Effects of individual risk and state housing factors on adverse outcomes in a national sample of youth transitioning out of foster care. *Journal of Adolescence*, *74*, 33–44. https://doi.org/10.1016/j.adolescence.2019.05.004

Putnam-Hornstein, E. (2011b). Report of Maltreatment as a Risk Factor for Injury Death: A Prospective Birth Cohort Study. *Child Maltreatment*, *16*(3), 163–174. https://doi.org/10.1177/1077559511411179

Putnam-Hornstein, E., & King, B. (2014). Cumulative teen birth rates among girls in foster care at age 17: An analysis of linked birth and child protection records from California. *Child Abuse & Neglect*, *38*(4), 698–705. https://doi.org/10.1016/j.chiabu.2013.10.021

Putnam-Hornstein, E., & Needell, B. (2011a). Predictors of child protective service contact between birth and age five: An examination of California's 2002 birth cohort. *Children and Youth Services Review*, *33*(8), 1337–1344. https://doi.org/10.1016/j.childyouth.2011.04.006

Putnam-Hornstein, E., Cederbaum, J. A., King, B., Cleveland, J., & Needell, B. (2013a). A population-based examination of maltreatment history among adolescent mothers in California. *The Journal of Adolescent Health: Official Publication of the Society for Adolescent Medicine*, *53*(6), 794–797. https://doi.org/10.1016/j.jadohealth.2013.08.004

Putnam-Hornstein, E., Cederbaum, J. A., King, B., Eastman, A. L., & Trickett, P. K. (2015). A Population-Level and Longitudinal Study of Adolescent Mothers and Intergenerational Maltreatment. *American Journal of Epidemiology*, *181*(7), 496–503. https://doi.org/10.1093/aje/kwu321

Putnam-Hornstein, E., Cleves, M. A., Licht, R., & Needell, B. (2013c). Risk of Fatal Injury in Young Children Following Abuse Allegations: Evidence From a Prospective, Population-Based Study. *American Journal of Public Health*, *103*(10), e39–e44. https://doi.org/10.2105/AJPH.2013.301516

Putnam-Hornstein, E., Needell, B., King, B., & Johnson-Motoyama, M. (2013b). Racial and ethnic disparities: A population-based examination of risk factors for involvement with child protective services. *Child Abuse & Neglect*, *37*(1), 33–46. https://doi.org/10.1016/j.chiabu.2012.08.005

Putnam-Hornstein, E., Lery, B., Hoonhout, J., & Curry, S. (2017). A Retrospective Examination of Child Protection Involvement Among Young Adults Accessing Homelessness Services. *American Journal of Community Psychology*, *60*(1–2), 44–54. https://doi.org/10.1002/ajcp.12172

Putnam-Hornstein, E., Webster, D., Needell, B., & Magruder, J. (2011c). A Public Health Approach to Child Maltreatment Surveillance: Evidence from a Data Linkage Project in the United States. *Child Abuse Review*, *20*(4), 256–273. https://doi.org/10.1002/car.1191

Raghavan, R., Brown, D. S., Allaire, B. T., Garfield, L. D., Ross, R. E., & Hedeker, D. (2015). Challenges in Using Medicaid Claims to Ascertain Child Maltreatment. *Child Maltreatment*, *20*(2), 83–91. https://doi.org/10.1177/1077559514548316

Rhodes, A. E., Boyle, M. H., Bethell, J., Wekerle, C., Goodman, D., Tonmyr, L., Leslie, B., Lam, K., & Manion, I. (2012). Child maltreatment and onset of emergency department presentations for suicide-related behaviors. *Child Abuse & Neglect*, *36*(6), 542–551. https://doi.org/10.1016/j.chiabu.2012.04.006

Rhodes, A. E., Boyle, M. H., Bethell, J., Wekerle, C., Tonmyr, L., Goodman, D., Leslie, B., Lam, K., & Manion, I. (2013). Child maltreatment and repeat presentations to the emergency department for suicide-related behaviors. *Child Abuse & Neglect*, *37*(2), 139–149. https://doi.org/10.1016/j.chiabu.2012.07.009

Rodriguez, J. M., & Shinn, M. (2016). Intersections of family homelessness, CPS involvement, and race in Alameda County, California. *Child Abuse & Neglect*, *57*, 41–52. https://doi.org/10.1016/j.chiabu.2016.06.004

Rose, R. A., & Lanier, P. (2017). A Longitudinal Study of Child Maltreatment and Mental Health Predictors of Admission to Psychiatric Residential Treatment Facilities. *International Journal of Environmental Research and Public Health*, *14*(10), 1141. https://doi.org/10.3390/ijerph14101141

Rossen, L., Tzoumakis, S., Kariuki, M., Laurens, K. R., Butler, M., Chilvers, M., Harris, F., Carr, V. J., & Green, M. J. (2019). Timing of the first report and highest level of child protection

response in association with early developmental vulnerabilities in an Australian population cohort. *Child Abuse & Neglect*, *93*, 1–12. https://doi.org/10.1016/j.chiabu.2019.04.007

Rouland, B., & Vaithianathan, R. (2018). Cumulative Prevalence of Maltreatment Among New Zealand Children, 1998–2015. *American Journal of Public Health*, *108*(4), 511–513. https://doi.org/10.2105/AJPH.2017.304258

Ryan, J. P., Jacob, B. A., Gross, M., Perron, B. E., Moore, A., & Ferguson, S. (2018). Early exposure to child maltreatment and academic outcomes. *Child maltreatment*, *23*(4), 365-375.

Schnitzer, P. G., Covington, T. M., Wirtz, S. J., Verhoek-Oftedahl, W., & Palusci, V. J. (2008). Public Health Surveillance of Fatal Child Maltreatment: Analysis of 3 State Programs. *American Journal of Public Health*, *98*(2), 296–303. https://doi.org/10.2105/AJPH.2006.087783

Schuck, A. M., & Widom, C. S. (2005). Understanding the Role of Neighborhood Context in the Long-Term Criminal Consequences of Child Maltreatment. *American Journal of Community Psychology*, *36*(3–4), 207–222. https://doi.org/10.1007/s10464-005-8615-1

Scott, K. M., McLaughlin, K. A., Smith, D. A. R., & Ellis, P. M. (2012). Childhood maltreatment and DSM-IV adult mental disorders: Comparison of prospective and retrospective findings. *The British Journal of Psychiatry*, *200*(6), 469–475. https://doi.org/10.1192/bjp.bp.111.103267

Scott, K. M., Smith, D. R., & Ellis, P. M. (2010). Prospectively Ascertained Child Maltreatment and Its Association With DSM-IV Mental Disorders in Young Adults. *Archives of General Psychiatry*, *67*(7), 712–719. https://doi.org/10.1001/archgenpsychiatry.2010.71

Segal, L., Nguyen, H., Mansor, M. M., Gnanamanickam, E., Doidge, J. C., Preen, D. B., Brown, D. S., Pearson, O., & Armfield, J. M. (2019). Lifetime risk of child protection system involvement in South Australia for Aboriginal and non-Aboriginal children, 1986–2017 using linked administrative data. *Child Abuse & Neglect*, *97*, 104145. https://doi.org/10.1016/j.chiabu.2019.104145

Spataro, J., Mullen, P. E., Burgess, P., Wells, D. L., & Moss, S. A. (2004). Impact of child sexual abuse on mental health: Prospective study in males and females. *British Journal of Psychiatry*, *184*(5), 416–421.

Ubbesen, M.-B., Gilbert, R., & Thoburn, J. (2015). Cumulative incidence of entry into out-of-home care: Changes over time in Denmark and England. *Child Abuse & Neglect*, *42*, 63–71. https://doi.org/10.1016/j.chiabu.2014.10.006

Vaithianathan, R., Maloney, T., Putnam-Hornstein, E., & Jiang, N. (2013). Children in the Public Benefit System at Risk of Maltreatment: Identification Via Predictive Modeling. *American Journal of Preventive Medicine*, *45*(3), 354–359. https://doi.org/10.1016/j.amepre.2013.04.022

Valuri, G. M., Morgan, F., Jablensky, A., Ambrosi, T. L., & Morgan, V. A. (2020). Impact of substance use and other risk factor exposures on conviction rates by people with a psychotic illness and other mental disorders. *Social Psychiatry and Psychiatric Epidemiology*, *55*(4), 517–525. https://doi.org/10.1007/s00127-019-01751-5

Van Horne, B. S., Caughy, M. O., Canfield, M., Case, A. P., Greeley, C. S., Morgan, R., & Mitchell, L. E. (2018). First-time maltreatment in children ages 2–10 with and without specific birth defects: A population–based study. *Child Abuse & Neglect*, *84*, 53–63. https://doi.org/10.1016/j.chiabu.2018.07.003

Van Horne, B. S., Moffitt, K. B., Canfield, M. A., Case, A. P., Greeley, C. S., Morgan, R., & Mitchell, L. E. (2015). Maltreatment of Children Under Age 2 With Specific Birth Defects: A Population-Based Study. *Pediatrics*, *136*(6), e1504–e1512. https://doi.org/10.1542/peds.2015-1274

Ven, E. van der, Dalman, C., Wicks, S., Allebeck, P., Magnusson, C., os, J. van, & Selten, J. (2015). Testing Ødegaard's selective migration hypothesis: A longitudinal cohort study of risk

factors for non-affective psychotic disorders among prospective emigrants. *Psychological Medicine*. https://doi.org/10.1017/S0033291714001780

Weiss, A. D. G., & Fantuzzo, J. W. (2001). Multivariate impact of health and caretaking risk factors on the school adjustment of first graders. *Journal of Community Psychology*, *29*(2), 141–160. https://doi.org/10.1002/1520-6629(200103)29:2<141::AID-JCOP1010>3.0.CO;2-6

Wilson, M. L., Tumen, S., Ota, R., & Simmers, A. G. (2015). Predictive Modeling: Potential Application in Prevention Services. *American Journal of Preventive Medicine*, *48*(5), 509–519. https://doi.org/10.1016/j.amepre.2014.12.003

Wong, J. W. Y., O'Donnell, M., Bayliss, D., Fletcher, J., & Glauert, R. (2017). Patterns of participation in year 9 academic testing and factors predicting absence on the day of test. *Educational Research for Policy and Practice*, *16*(2), 109–127. https://doi.org/10.1007/s10671-016-9195-6
